# Candidate Proteins, Metabolites and Transcripts in the Biomarkers for Spinal Muscular Atrophy (BforSMA) Clinical Study

**DOI:** 10.1371/journal.pone.0035462

**Published:** 2012-04-27

**Authors:** Richard S. Finkel, Thomas O. Crawford, Kathryn J. Swoboda, Petra Kaufmann, Peter Juhasz, Xiaohong Li, Yu Guo, Rebecca H. Li, Felicia Trachtenberg, Suzanne J. Forrest, Dione T. Kobayashi, Karen S. Chen, Cynthia L. Joyce, Thomas Plasterer, on behalf of the Pilot Study of Biomarkers for Spinal Muscular Atrophy (BforSMA) Trial Group

**Affiliations:** 1 Departments of Neurology and Pediatrics, The Children's Hospital of Philadelphia and The University of Pennsylvania School of Medicine, Philadelphia, Pennsylvania, United States of America; 2 Departments of Neurology and Pediatrics, The Johns Hopkins University, Baltimore, Maryland, United States of America; 3 Departments of Neurology and Pediatrics, University of Utah School of Medicine, Salt lake City, Utah, United States of America; 4 Department of Neurology, Columbia University, New York, New York, United States of America; 5 BG Medicine, Inc., Waltham, Massachusetts, United States of America; 6 New England Research Institutes, Inc., Watertown, Massachusetts, United States of America; 7 Spinal Muscular Atrophy Foundation, New York, New York, United States of America; 8 Department of Chemistry and Chemical Biology, Northeastern University, Boston, Massachusetts, United States of America; National University of Singapore, Singapore

## Abstract

**Background:**

Spinal Muscular Atrophy (SMA) is a neurodegenerative motor neuron disorder resulting from a homozygous mutation of the survival of motor neuron 1 (SMN1) gene. The gene product, SMN protein, functions in RNA biosynthesis in all tissues. In humans, a nearly identical gene, SMN2, rescues an otherwise lethal phenotype by producing a small amount of full-length SMN protein. SMN2 copy number inversely correlates with disease severity. Identifying other novel biomarkers could inform clinical trial design and identify novel therapeutic targets.

**Objective:**

To identify novel candidate biomarkers associated with disease severity in SMA using unbiased proteomic, metabolomic and transcriptomic approaches.

**Materials and Methods:**

A cross-sectional single evaluation was performed in 108 children with genetically confirmed SMA, aged 2–12 years, manifesting a broad range of disease severity and selected to distinguish factors associated with SMA type and present functional ability independent of age. Blood and urine specimens from these and 22 age-matched healthy controls were interrogated using proteomic, metabolomic and transcriptomic discovery platforms. Analyte associations were evaluated against a primary measure of disease severity, the Modified Hammersmith Functional Motor Scale (MHFMS) and to a number of secondary clinical measures.

**Results:**

A total of 200 candidate biomarkers correlate with MHFMS scores: 97 plasma proteins, 59 plasma metabolites (9 amino acids, 10 free fatty acids, 12 lipids and 28 GC/MS metabolites) and 44 urine metabolites. No transcripts correlated with MHFMS.

**Discussion:**

In this cross-sectional study, “BforSMA” (Biomarkers for SMA), candidate protein and metabolite markers were identified. No transcript biomarker candidates were identified. Additional mining of this rich dataset may yield important insights into relevant SMA-related pathophysiology and biological network associations. Additional prospective studies are needed to confirm these findings, demonstrate sensitivity to change with disease progression, and assess potential impact on clinical trial design.

**Trial Registry:**

Clinicaltrials.gov NCT00756821.

## Introduction

Spinal Muscular Atrophy (SMA) is a monogenic, recessively inherited neuromuscular disorder caused by loss-of-function mutation of the Survival Motor Neuron 1 (SMN1) gene [1]. A neighboring nearly-identical copy of this gene, SMN2, is invariably present in individuals with SMA. SMN2 produces transcripts that are predominantly spliced to a truncated form in which exon 7 is deleted. However, some full-length transcript is generated that produces normal full-length SMN protein. SMN2 partially ameliorates disease in SMA, as the number of SMN2 copies inversely correlates with phenotypic severity [2]. Much attention has thus been directed to potential therapies that increase synthesis of full-length protein from the SMN2 gene [3]–[10]. Of the clinical trials to date performed using available FDA-approved pharmaceuticals with ability to increase SMN expression *in vitro,* none have demonstrated apparent efficacy against placebo [11]–[14]. These medications, sodium phenylbutyrate, valproic acid and hydroxyurea, increase full length SMN transcript and protein but apparently not to a sufficient degree to demonstrate clinical benefit in clinical trials to date. These medications were not specifically designed for treatment of SMA. Potential limitations of these studies also include not knowing the optimal dosage or duration of the study, and whether the putative increase in SMN protein will help rescue a pool of non-functioning but alive motor neurons or stabilize the existing pool of functioning motor neurons and limit the rate of further clinical decline. Several new SMA therapeutics that are expressly designed to upregulate SMN are in different stages of development and biomarkers are thus urgently needed to assist in their advancement [15].

Identification, validation and qualification of biomarkers that correlate with disease severity will have a substantial impact on SMA basic and clinical science in multiple ways. The clinical course of SMA poses challenges to clinical trial design [16]–[18] **.** Across the range of disease severity and onset age, most individuals with SMA manifest three broad phases over the course of their disorder. An initial presymptomatic phase is followed by an early symptomatic phase characterized by either frank regression in strength or function or a failure to achieve normally expected developmental gross motor milestones. This is then followed by a chronic plateau phase characterized by a variable period of stability or much slower decline in function over a period of several months to years. This pattern of evolution of symptoms, and the potentially variable points along their course at which individuals with SMA may enter trials, makes it challenging to decide which endpoints, cohorts and trial duration will be most effective in determining whether or not a given therapeutic intervention is of value. SMA biomarkers could substantially improve the performance of clinical trials by (1) expanding the population of potential study subjects by reducing “floor” and “ceiling” effects of current measures, (2) permitting “early look” assessment of treatment futility or predicting clinical response in otherwise prolonged duration trials, (3) easing the burden of travel and outcome assessment in medically fragile patients, and (4) enabling simpler, more cost effective and efficient clinical trial design.

In addition, biomarkers found in an unbiased manner can be used to develop hypotheses about cellular pathophysiology, or validate hypotheses derived from basic science studies of SMN biology. The cellular basis of specific motor neuron vulnerability to low SMN protein levels remains puzzling as SMN is expressed in all tissues, and is essential to eukaryotic mRNA splicing [19]–[22]. Hints of special neuronal vulnerability to defects in RNA handling are emerging with the identification of other putative RNA-related genes [23]–[25] as the cause of motor neuron disorders [26]. Identification of biomarkers (or a correlated biomarker network) that associate with SMA disease severity could identify abnormal signaling pathways, constrained essential substrates, or toxic products that have pathophysiologic importance. Biomarkers identified by an unbiased approach may also lead to important clues that would be missed by other approaches through identification of novel factors affecting or associated with disease severity.

While SMN transcript or protein levels are obvious biomarkers for therapeutics targeting SMN production (see companion paper, Crawford et al. [61]), there are compelling reasons why SMA biomarker discovery should be expanded beyond SMN. Measures of SMN expression in blood may not reflect the critical level of expression of the protein in the CNS. A wide variability or fluctuations of transcript and protein analysis of SMN levels in blood may undermine its value as a clinical tool. Moreover, SMN expression as an outcome biomarker identifies factors acting only at that aspect of pathogenesis, missing those factors downstream of SMN abundance that might influence phenotype. An unbiased approach to biomarker identification includes the prospect of identifying potential targets of therapy downstream of SMN production.

Recent advances in bioanalytical technology and informatics have enabled systems level biomarker discovery efforts based on metabolomics, transcriptomics, and proteomics [27] (hereafter referred to as “-omics”). The present “BforSMA” (Biomarkers for SMA) project is a comprehensive -omics investigation of unprecedented scale and scope. Traditionally, previous studies of neurodegenerative disorders have tended to be hypothesis-based efforts focused upon markers representing preselected pathophysiologies [28]–[30]. Or, in prior studies that were unbiased to disease mechanism, evaluations were performed on cohorts with significant potential confounders [31], [32]. Often, these studies have been conducted in a manner that was insensitive to markers of disease severity, rate of progression, or disease stage at time of sample acquisition [32]–[35] or were restricted to evaluation of either small-molecule metabolites, proteins, transcript expression profiles [30]–[32], [34]–[37], or a subset of these [37].

The BforSMA project combines three elements designed to maximize the potential to identify scientifically and clinically relevant novel biomarkers. The first of these is an unbiased metabolomics, proteomics, and transcriptomics analysis of samples obtained at a single time point. The second includes recruitment of a well-characterized disease cohort, free of potential confounding variables, encompassing a relatively broad range of disease phenotype to account for the singular characteristics of SMA variability and clinical course. The third is the inclusion of an age-matched normal control cohort for secondary class-specific comparisons [38]. The resulting BforSMA data set is intended to be hypothesis-generating, and thus may include false positive “hits” in order not to exclude potentially important biomarkers. The cross-platform dataset derived from this project is expected to provide a rich resource to spur future investigations.

This unbiased discovery project for biomarkers of SMA function is one of two biomarker projects performed on the same cohort in parallel. The other, specifically targeting measures of SMN expression in blood, is presented separately {see companion paper, Crawford et al. [61]). In this report we describe the overall project design, characteristics of the recruited cohort, and provide a first level analysis of non-SMN biomarkers correlated with multiple functional outcome measures.

## Materials and Methods

### Planning and Objectives

The primary objective was to identify biomarkers associated with gross motor function as measured by the Modified Hammersmith Functional Motor Scale (MHFMS) [39] across a broad range of clinical phenotypic severity in children with SMA types I, II, and III. Secondary objectives were to identify (1) biomarkers associated with the other clinical characteristics identified in Table 1 and Table S1, (2) class biomarkers that distinguish SMA from normal age-matched controls, and (3) networks of biomarker associations that suggest specific biochemical or signaling pathways of potential import to SMA pathogenesis. SMN-related measurements and correlations are detailed in the accompanying manuscript (see companion paper, Crawford et al. [61]). All study protocols were done in accordance with GCP, IRB and local and federal regulations.

**Table 1:** Clinical data of SMA and Control Subjects.

	SMA Type I	SMA Type II	SMA Type III	Control	p-value*	p-value*
	N = 17	N = 49	N = 42	N = 22	I vs IIvs III	SMA vs Control
**Age**						
mean (SD)	5.70 (3.54),	6.55 (3.40),	7.51 (3.11),	6.95 (3.29),	0.14	0.84
median [range]	4.03 [2.4–12.7]	6.49 [2.2–13.0]	7.42 [2.4–13.0]	6.02 [2.2–13.0]		
**Sex,** n (%)					0.73	0.64
Male	10 (59%)	26 (53%)	20 (48%)	10 (46%)		
2–5 years old	6	15	7	5		
6–12 years old	4	11	13	5		
Female	7 (41%)	23 (47%)	22 (52%)	12 (54%)		
2–5 years old	5	9	8	6		
6–12 years old	2	14	14	6		
**MHFMS,**						
mean (SD)	0 (0)	14.02 (10.5)	34.1 (10.0)	39.8 (0.7)	<0.001	<0.001
median [range]	0 [0–0]	11 [0–36]	40 [2]–[40]	40 [37]–[40]		
**Current Motor Function**, n (%)					<0.001	<0.001
Sit	0 (0%)	42 (85.7%)	41 (97.6%)	22 (100%)		
Walk	0 (0%)	0 (0%)	32 (76.2%)	22 (100%)		
**Feeding Method**					<0.001	0.07
solid food	1 (5.9%)	44 (89.8%)	41 (97.6%)	22 (100%)		
modified oral intake	2 (11.8%)	2 (4.1%)	1 (2.4%)	0 (0%)		
G-tube fed	14 (82.4%)	3 (6.1%)	0 (0%)	0 (0%)		
**BMI z-Score**					0.04	0.17
mean (SD)	−2.54 (3.98)	−0.90 (4.13)	−0.09 (1.65)	0.16 (1.02)		
median [range]	−1.34 [−9.7 to 3.4]	−0.12 [−19.7 to 3.6]	−0.05 [−4.2 to 3.2]	0.34 [−1.8 to 1.9]		
**FVC% Predicted**					<0.001	0.08
mean (SD)	N/A	70.04 (30.10)	104.1 (18.9)	103.3 (10.6)		
median [range]	N/A	66.5 [26–135]	105 [61–153]	103 [87–125]		
**10 meter walk**						
mean (SD)	NA	NA	10.1 (6.1)	3.5 (1.5)	NA	<0.001
median [range]	NA	NA	9 [3.4–34]	3 [2.2–8.8]		
**Age of Disease Onset**				NA	<0.001	NA
0–6 months	17 (100%)	10 (20.4%)	1 (2.4%)			
7–17 months	0 (0%)	35 (71.4%)	20 (47.6%)			
18+months	0 (0%)	4 (8.2%)	21 (50.0%)			
**Respiratory support**					<0.001	0.001
BIPAP or tracheostomy	15 (88%)	8 (16%)	0 (0%)	0 (0%)		
Cough assist	1 (6%)	14 (29%)	1 (3%)	0 (0%)		

* ANOVA for continuous variables; Fisher exact test for categorical variables.

There were no significant differences in age or gender across the recruitment cohorts. A key goal of this study was to minimize the confounding correlation between present age and functional status. This goal was largely achieved, both overall and within SMA groups Type II and III, and to a partial extent, SMA Type I through a competitive recruitment plan managed through the data coordinating center at the New England Research Institutes (NERI). The Modified Hammersmith Motor Function Scale differentiated between SMA subjects and controls and between Type I, II and III subjects, as did respiratory support, reflecting current level of function. FVC and the nutritional assessment score significantly distinguished between SMA type; however, BMI proved to be far less discriminatory.

### Ethics Statement

Institutional review board (IRB) approval for the protocol was obtained from each BforSMA clinical site before enrollment at that site and from a central Institutional Review Board, New England Research Institutes. Written informed consent for participation was obtained from the legal guardians of all subjects and assent for participation was obtained directly from subjects whenever applicable. This trial was registered with ClinicalTrials.gov with identifier NCT00756821.

### Study Design, Sample Size Determination and Enrollment

A cross-sectional, single visit, multi-center, exploratory study design was employed. Blood and urine samples were collected for biomarker analysis and DNA samples were collected for *SMN2* copy number determination. No therapeutic intervention occurred. The full protocol is available in the Appendix S1. Key inclusion and exclusion criteria for this study were:

Inclusion Criteria:

For all groups:

- Age 2 to 12 years, inclusive.
- In good health in the judgment of the clinical investigator at the time of assessment.

Specific group requirements – SMA children:

- Documented homozygous deletion of SMN1 (exon 7).
- SMA Type I defined as the inability ever to sit unaided in the judgment of the clinical investigator.
- SMA Type II defined as the ability to ever sit unaided for >30 seconds on a flat surface in the judgment of the clinical investigator.
- SMA Type III defined by the ability to ever stand unaided for 30 seconds and walk unaided for >30 ft.

Specific group requirements – Control children:

- Otherwise healthy children that may be either genetically-related siblings of SMA children (confirmed non-carriers) or unrelated children.

Exclusion Criteria:

For all groups:

- Systemic or specific-organ illness, including renal, hepatic, cardiac, pulmonary, significant gastrointestinal illness, hematologic or rheumatic disorders requiring ongoing treatment or chronic medication use.
- Any known genetic condition other than Spinal Muscular Atrophy requiring pharmaceutical treatment.
- Use of any putative SMN-enhancing medications or treatments for 14 days prior to enrollment including, valproic acid, phenylbuterate, and hydroxyurea.
- Use of carnitine, creatine, oral albuterol and riluzole for 14 days prior to enrollment.
- Use of any oral prescription medications for 14 days prior to enrollment with the exception of the following medications, which are allowed: anti-reflux medications (e.g. rantidine), constipation or stool softening medications (e.g. polyethylene glycol 3350), stool bulking agents, and inhaled bronchodilator medications and nebulizers (e.g. albuterol).
- Any illness requiring treatment with antibiotics or anti-inflammatory medication within the past 14 days.
- Participation in a clinical trial (except observational studies) within the previous 14 days.

The number and type of subjects to be enrolled was based upon clinical and statistical considerations. An age range of 2 to 12 years was chosen in an attempt to limit confounding factors associated with developmental maturation or inability to reliably perform the outcome measures in the youngest children, and metabolic changes associated with puberty at the older age ranges. Subjects were required to have a documented homozygous deletion of exon 7 in the SMN1 gene. There was special emphasis on enrolling children as subjects across the range of phenotype severity that fall within the eligibility criteria.

Clinical definitions of SMA type were based upon maximum achieved gross motor function (see inclusion criteria, above). This is revised from the classification initially proposed by Munsat et al. [40], and further modified by Zerres et al. [41].

Individuals were excluded from enrollment if they had any illness or were on any the categories of medications considered possible confounders to interpretation of the data within 2 weeks of their participation (see exclusion criteria, above). Subjects were eligible for enrollment if those medications or supplements had been discontinued at least 2 weeks prior to the study visit.

An unbalanced number of subjects from the three types of SMA was targeted (17 Type I, 45 Type II, and 40 Type III SMA subjects) in part out of concern that recruitment of eligible subjects with SMA Type I would be difficult because of their high level of inter-current illness and mortality in childhood and the lower age limit specified. Given the floor effects of the MHFMS in this population, all subjects with Type I, regardless of severity, would receive a score of 0. The sample size for the SMA subjects was selected to achieve 83% power for the primary outcome of MHFMS to detect a biomarker set associated with motor function using multivariate elastic net regression analysis [42], assuming a 0.75 correlation between the observed and theoretical outcomes. The power estimate is based on an average of 100 simulated datasets using data available from the original Hammersmith Scale [43]. The sample size of 20 control subjects was selected to achieve at least 90% power to detect a univariate biomarker with a mean-fold change (MFC) of 1.5 when the false discovery rate is controlled at 0.05 [44]. In both power calculations it was assumed that 10% of profiled analytes are true biomarkers, and that variance of analytes is equal to 0.2.

### Sample Processing and Storage

Sample collection, handling and chain of custody procedures were designed to ensure the best quality specimens for biomarker analysis. We targeted collection of up to 10 mL of blood and at least 4 mL of urine from each subject. Six milliliters of whole blood was transferred into an EDTA tube upon collection and the remaining blood sample was poured into a CPT tube. Four milliliters of urine was aliquoted equally into two different cryovials, for a total of 2 mL per cryovial. Excess urine of up to 4 mL was aliquoted into a third cryovial. EDTA tubes with 6 mL of blood were inverted five times. Two 0.75 mL aliquots of whole blood from the EDTA tube were put in separate cryovials to make the DNA and RNA samples. CPT tubes with 4 mL of blood were gently inverted 8–10 times and centrifuged at 3,000RPM for 25 minutes at room temperature (18–25°C). The CPT tubes were gently inverted again for 5–10 times. The PBMC layer was poured into a 5 mL PBMC vial. Samples were shipped from study sites to the central lab, PPD, Inc. (Kentucky). DNA, RNA, and urine samples were transported on dry ice and stored in a −70°C/-80°C freezer. PBMC samples were stored at room temperature. Protein and small molecule metabolites from plasma and urine samples were analyzed by BG Medicine, Inc. (Waltham, MA), and mRNA exons and transcripts from blood by Expression Analysis, Inc. (Durham, NC). Statistical analyses were performed by BG Medicine and NERI.

### Experimental Platforms

#### Proteomics

The quantitative discovery proteomics workflow was based on multi-dimensional liquid chromatography – MS/MS analyses of peptides combined with 8-plex iTRAQ labeling [45]. To achieve sufficient dynamic range of plasma analysis and long-term reproducibility, a two-stage protein depletion method was optimized [46]. In the first depletion stage, 14 abundant plasma proteins are depleted by an IgY14 antibody column (Sigma-Aldrich, St. Louis, MO, USA) (serum albumin, IgG, fibrinogen, transferrin, IgA, IgM, haptoglobin, α-2-macroglobulin, α-1-acid glycoprotein, α-1-antitrypsin, Apo A-I, Apo A-II, complement C3, and Apo B-100) The flow-through was further depleted by a Supermix column (Sigma-Aldrich), which retains moderately abundant proteins with a broad specificity. Proteins in the Supermix flow-through are recovered on a reversed-phase column.

Protein samples were reduced with TCEP, alkylated with iodoacetamide, and digested with trypsin. Following digestion each sample is labeled with a discrete iTRAQ reagent. Six of the eight channels were utilized for primary samples and two were used for reference samples created by combining aliquots from each primary sample in the study. Labeled samples were combined into an iTRAQ mix and fractionated by strong cation exchange into six fractions. Each of the fractions were further fractionated by HPLC and spotted on MALDI plates for MSMS analysis using an AB/SCIEX 4800 TOF/TOF mass spectrometer (MDS SCIEX, Concord, ON, Canada). Acquisition of LC-MSMS data was optimized by in-house developed algorithms to select and measure consistent sets of peptides from experiment to experiment.

Relative quantification of peptides was carried out by determining relative intensities of reporter ions between the sample specific channels (*m/z* 114, 115, 116, 118, 119, 121) and reference sample channels (*m/z* 113, 117). The average ratios relative to the two reference channels were used in most experiments.

Identification of peptides from the MS/MS spectra was achieved using the Mascot database searching tool [47] and a BGM-developed validation protocol to distinguish true and false positive peptide matches. Once data collection was completed for every study sample, peptides were assigned to a minimum non-redundant protein set. Relative quantification of proteins was achieved by assigning the median ratio from peptides mapped to the given protein. Normalization of protein expression data was carried out using a procedure described by Vandersompele et al. [48].

#### Transcriptomics

RNA was isolated using the Ambion RiboPure™ blood kit (Austin, TX, USA) according to manufacturer’s instructions. Two amplification steps were performed using the NuGEN Ovation© Pico WTA system protocol (San Carlos, CA, USA). cDNA purification columns were used after the SPIA and post Exon modules.

105 SMA and 21 control subject samples were measured for exon array analysis using the Affymetrix GeneChip Human Exon 1.0 ST Array (Fremont, CA, USA). Affymetrix probe sets were normalized using the RMA method [49].

#### Metabolomics

Metabolomics profiling was conducted on organic extracts of plasma samples using multiple analytical platforms. Plasma lipids were analyzed by LC/MS profiling on a QStar Elite Quadrupole Time-of-Flight instrument (MDS/SCIEX, Concord, ON, Canada). Amino acid analysis (AAA) was carried out as a targeted analysis of 42 species using Multiple Reaction Monitoring (MRM) on a 4000Qtrap instrument (MDS/SCIEX, Concord, ON, Canada). Free fatty acid (FFA) analysis targeted 57 free – unesterified – fatty acids by GCMS following the methylation of these compounds. GC/MS of plasma and urine samples was completed in a semi-targeted fashion: a list of analyte targets was created from a preliminary profiling experiment. The target peaks were then measured in all of the study samples. This platform used an Agilent single-quadrupole mass analyzer (Model 5975, Agilent, Santa Clara, CA). The lipid and GC/MS platforms used a set of universal internal standards (5–8 non-endogenous compounds) for quantification. The amino acid analysis (AAA) and free fatty acid (FFA) platforms relied on calibrated internal standards for each of the target compounds thereby facilitating absolute quantification.

To facilitate the generation of reproducible data, analytical runs were organized into batches. The batch size is defined by the number of samples that can be comfortably prepared for mass spectrometric analysis in less than a day. To be able to correct for batch-to-batch variations and to identify within-batch drifts in the analytical performance, technical replicates of a Quality Control (QC) sample are inserted at a regular interval into the run sequence. These QC samples were prepared from pooling an equal aliquot from each primary sample in the study. The QC samples are processed identically to the primary samples and results for the QC samples are used to monitor data quality. The order of acquisition of the primary samples was determined by a randomization scheme to minimize the occurrence and effect of systematic variations in/on workflow performance.

The FFA platforms uses isotope labeled fatty acid standards for absolute quantification. 41 out of the 57 targeted compounds reported by the method have their own internal standard and 16 additional fatty acids are determined against a calibration curve shared with another compound. In this platform fatty acids are converted into their form methyl esters and analyzed by GC/MS.

#### Lipid Profiling

Lipid measurements were performed on all 129 primary samples in the BforSMA study as well as 24 reference (QC) samples (153 samples in total). Analysis of the 153 samples was completed in three batches.

Plasma samples were extracted with a solvent of 25%:10%:65% dichloromethane:isopropanol:methanol. All samples (primary and QC) were spiked with five internal standards that were used to track platform performance and for data normalization: 14∶0 LPE, 17∶0 LPC, 24∶0 PC, 40∶0 PC, and 51∶0 TG.

LC/MS profiles of the samples were processed with a set of pipelined procedures for (LC) peak detection, peak alignment, and peak family clustering to consolidate multiple ionized forms of a lipid into a single component, normalization, and batch correction. Aligned components were quantified in terms of their processed peak intensities.

Identification of lipid components was completed by a combination accurate mass-retention time matching to known lipid species characterized previously at BGM and by LC-MS/MS analysis of the lipid extracts. Additionally, adduct pattern and nitrogen-rule were utilized to establish the unambiguous identity of the detected lipids.

#### Amino Acid Analysis

The AAA platform targeted 42 L-amino acids (including all essential amino acids). Methanol extracts of the plasma samples (10 µL) were labeled with an iTRAQ reagent (Applied Biosystems, Foster City, CA, USA) producing the *m/z* 115 reporter fragment. Known concentrations of amino acid standard labeled with the *m/z* 114 reagent were added to the sample and analyzed by LC-MRM. For each amino acid target to transitions were monitored: MH^+^−> 114 for the internal standard and MH^+^−> 115 for the unknown. The intensity ratio of the peaks, *m/z* 115/*m/z* 114, scaled with the known concentration of the standard yielded the amino acid concentrations in µM units.

Amino Acid Analysis (AAA) data were generated for all 129 primary samples as well as 24 reference samples (153 samples in total). Samples were processed in three batches. Peak integration was performed using MultiQuant (MDS/SCIEX) software. The concentration of each detected amino acid was calculated using the in-house data processing pipeline.

#### Gas Chromatography/Mass Spectrometry (GC/MS) Analysis

For GC/MS analysis plasma samples (50 µL) or dried urine samples (from 250 µL urine) were extracted with methanol. Dried extracts were trimethylsilylated (TMS of hydroxyl-, carboxyl-, and amino-functionalities), and oximated (oxo- functionalities), in order to make analytes volatile for GC separation. To improve analytical precision the injections were made in duplicate and the mean intensities were recorded. 128 primary samples and 54 QC samples were processed in 9 batches.

Due to the inherent complexity involved in analyte assignment to peaks identified in GC/MS, a target peak list, was carried through the analysis. Peak intensities were normalized to one of the 8 internal standards added to each sample. The most appropriate internal standard for each analyte was based on determining which internal standard exhibited the lowest variability in the QC samples for that given analyte. Peak normalization and batch correction were performed by pipelined procedures. Following statistical analysis of the data to determine marker status, identification of unknown analytes was carried out based on a priority list. Stronger markers (in terms of p-values) were assigned higher priorities than weaker markers or non-markers. Identification of unknown analytes was attempted by a combination of matching to GC/MS library spectra (in terms of retention time and fragment masses), de novo interpretation, and purchasing standard compounds and comparing fragmentation pattern. Since plasma analytes were better represented in our GC/MS library than urine analytes, a better identification rate could be achieved for plasma analytes (∼70% versus ∼50% in urine).

#### Free Fatty Acid Analysis

Free fatty acids – fatty acids not esterified to lipids – were measured by GC/MS of the corresponding fatty acyl methylesters in a format of isotope dilution measurements. 41 of the 57 target compounds were calibrated against their own isotope labeled internal standards and the remaining analytes were calibrated against one of the 48 standards.

Free fatty acid (FFA) analysis was obtained on 129 primary samples as well as 10 reference or QC samples. The project was completed in two batches. Because of the chemical stability of these compounds including the internal standards, batch correction was not necessary for this platform and the relatively small number of QC samples was utilized only for assessing the reproducibility of the measurements.

### Data Analysis

A summary of continuous and categorical outcome measures is provided in Table S1.

#### Univariate Continuous Outcomes

The set of seven (7) univariate continuous outcomes collected includes the Modified Hammersmith Functional Motor Scale (MHFMS), Age at Disease Onset (eight categories: 0–3 months, 4–6 months, 7–11 months, 12–17 months, 18–23 months, 24–35 months, 3-6 years, 6–12 years; treated as a continuous variable), BMI Z-score, Forced Vital Capacity (FVC), 10 Meter Timed Walk Test, SMN Protein Levels and SMN2 Copy Number. The predictor variables are analyte concentration (absolute concentration where available, or relative concentration otherwise) and potential confounders include age and gender. A univariate multiple linear regression model (LM) was fitted. Analytes with fewer than 20 complete measurements were excluded from this analysis. The linear regression model may be written as:


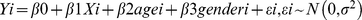
where i indicates the *i^th^* subject, *Y* is the outcome of interest; *X_i_* is the analyte intensity in natural log scale. The intercept *ß_0_*, coefficient for analyte amount ß_1_, coefficient for age and gender *ß_2_,ß_3_* and variance σ*^2^* of the error term *ɛ_i_* were estimated.

#### Univariate Categorical Outcomes

The set of eight (8) univariate categorical outcomes includes SMA type (two categories: disease and control; three categories: Type I, Type II, Type III; and four categories: Type I, Type II, Type III and Control), Current Level of Function (3 categories: non-sitters, sitters, walkers; and 5 categories: Type I, Type II non-rolling, Type II rolling, Type III non-stair climbing, Type III stair climbing), Age at Disease Onset (3 categories: 0–6 months, 7–17 months, ≥ 18 months), Nutritional Assessment (two categories: modified intake/G-tube fed and solid food), Respiratory Support (4 categories; none, cough assist, Bi-level Positive Airway Pressure, (BiPAP) <16 hrs, BiPAP >16). The goal was to test whether the analyte abundance is similar across all the categorical levels, adjusting for potential confounding factors of age and gender. If this test was rejected (*i.e.* a difference does exist), the divergent categories were identified. For this purpose, an analysis of covariance model (ANCOVA) was fitted to the data. Analytes that were measured in fewer than ten subjects in either category being compared were excluded from analysis. The ANCOVA model can be written as:


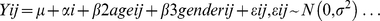
where *i* indicates the *i^th^* factor level, *j* indicates the *j^th^* subject in the *i^th^* factor level, *Y_ij_* is the analyte abundance for the *j^th^* subject in the *i^th^* factor level. α_i_ is the factor level effect subject to


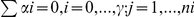
All analytes were first tested for difference of abundance across all categories.



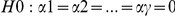




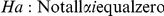
If the abundance of an analyte was found to differ across factor levels, pairwise comparisons of factor level means were subsequently performed to assess these differences (e.g. sign, and magnitude).



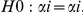





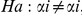



The analyte was declared a candidate marker if both the overall test and at least one of the pairwise comparisons were statistically significant.

#### Controlling the False Discovery Rate

In each analysis, tests of statistical significance with hundreds of analytes across all bioanalytical platforms were performed simultaneously. Therefore, some tests were likely to have significant results by chance alone, resulting in false discoveries. In order to control the false discovery rate (FDR) among all significant findings, we applied the method described in Benjamini and Hochberg [50], which transforms the individual p-values into FDR-corrected p-values. Analytes were classified as markers if the p-value was less than 0.05 and the FDR-adjusted p-value was less than 0.10.

## Results

### Characteristics of the Study Cohort

A total of 130 subjects enrolled from 18 sites in the United States and Canada included 17 Type I, 49 Type II, 42 Type III SMA, and 22 healthy control subjects. For consented subjects undergoing study screening procedures there were 46 screen failures. The two major reasons for screen failures were taking prohibited medicines and lack of documented homozygous deletion of SMN1. Enrollment figures slightly exceeded the original targets for each group due to protocol permitted subject replacement for insufficient quantity (n = 7) or quality (n = 3) of specimens. All 18 study sites contributed at least one subject over the course of 18 weeks concluding in March 2009. The number, age and gender distribution of those enrolled is found in Table 1. Although age of onset correlates with phenotype severity across the range of individuals with SMA, a key recruitment goal of this study was to minimize, within the ascertained cohort, the correlation between present age and present functional status – because such correlation might introduce an age bias into identified markers. This goal was achieved both overall and within SMA groups Type II and III, and to a partial extent, SMA Type I.

MHFMS scores were obtained for controls and all subjects with SMA Type II and III, as well as 10-meter walk time for controls and all Type III subjects able to walk (n = 34). Type I subjects are unable to perform any items on the MHFMS, and thus were assigned the minimum MHFMS score of zero. Forced vital capacity was available for all subjects over age 5 and/or children able to comply with the assessment (n = 69). All measures of function, present functional state, and the historical age of disease onset were spread over a wide range (Table 1). FVC, including many non-ambulatory subjects assessed by the MHFMS, correlated with the MHFMS, while the 10 meter walk, which assesses those above the range of this scale, did not. BMI- z-score did not correlate with MHFMS or age (Table 2). BMI z-score is a complex feature that can both affect MHFMS, when obesity limits mobility, or be affected by it, as very low muscle mass may itself reduce BMI z-score as well as impair adequate nutrition.

**Table 2:** Correlations of secondary measures with MHFMS and age for SMA subjects.

	MHFMS	Age
**Age**	0.07 (p = 0.45)	
**BMI z-score**	0.17 (p = 0.09)	0.12(p = 0.20)
**FVC % predicted**	0.70 (p<0.001)	−0.14(p = 0.31)
**10 meter walk**	−0.28 (p = 0.13)	−0.36(p = 0.045)

Of the secondary outcome measures, only forced vital capacity (FVC) significantly correlated with the primary outcome measure – the Modified Hammersmith Motor Function Scale. While BMI is thought to have a strong effect on function in individuals affected by SMA, the correlation did not reach significance here. Not surprisingly, FVC and walking were negatively correlated with age.

Subject cooperation was an important factor in data and sample collection in this study. In at least 14 subjects, collection of urine or blood samples was not within strict compliance with the protocol. Urine samples proved to be the more difficult to collect than blood. There were no concerns identified in sample handling, chain of custody and shipment procedures. Close attention was paid to data quality during data collection using real-time QC monitoring. Reproducibility of measurements was characterized by observing the coefficients of variance (expressed in %CV) obtained from the replicates of QC samples. Median %CVs were around 15% of the proteomics and lipid platforms, and below 10% for GC/MS, amino acid, and free fatty acid analyses (see Table S2).

Use of at least 12 different classes of medication (see Appendix S2) was reported in this fragile study population. Investigators were asked to use best clinical judgment when considering discontinuation of medication prior to enrollment. Two subjects were found to have been on prohibited medications post-enrollment (methylphenidate [n = 1] and glycopyrrolate [n = 1]). These samples were not excluded from the analysis, but subject to post-hoc review for unexpected outliers that might plausibly be associated with the medication.

### Summary of Primary Analysis

Of the 1184 analytes measured across the proteomics and metabolomics platforms, there were 490 analytes that were significantly associated with at least one of the 11 outcome measures or endpoints assessed in the study (Table 3). Of these, 200 analytes correlated with the MHFMS, the primary outcome measure of this study; many were also associated with secondary outcome measures and there were some hits seen in multiple platforms. Table 3 shows the results of the primary outcome analysis across the seven platforms utilized in this study. The complete results of the univariate analyses of the MHFMS outcome measure versus analytes across all platforms are reported in Tables S3, S4, S5. The BforSMA data is available publically upon publication of this report through the Neuroscience Information Framework (http://neuinfo.org/bforsma).

**Table 3:** Results from primary outcome analysis across all seven “omic” platforms.

Sample Type	Platform	Number of Assayed Analytes	Number of “Markers” that Associate with MHFMS
Plasma	LC-MALDI-MS/MS Proteomics	701	**97**
Plasma	Lipid LC/MS Metabolomics	71	**12**
Plasma	GC/MS Metabolomics	160	**28**
Plasma	Amino Acid Analysis	37	**9**
Plasma	Free Fatty Acid Analysis	47	**10**
Urine	GC/MS Metabolomics	168	**44**
Whole Blood	Affymetrix Exon Array	807,038	**0**

The overall number of hit markers identified that significantly associate with the MHFMS primary outcome measure is 16.9% when the exon array analysis is excluded.

To create a more manageable dataset for further evaluation and inspection, candidate markers were prioritized for each platform based on statistical significance of the Q-value and strength of association with the primary outcome measure in addition to correlations with secondary outcome measures. The top 20 markers across platforms are reported in the sections below.

### Plasma Proteomics

**Figure 1:**
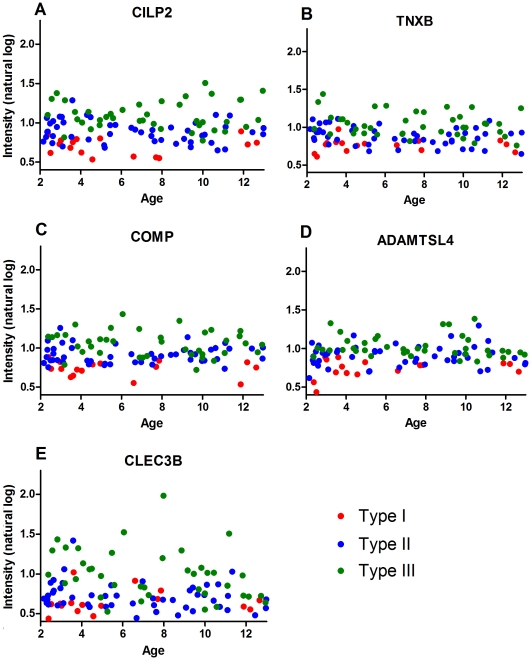
Illustration of the top 5 markers as candidate biomarkers by age. The natural log intensity of the protein abundance of CILP2, TNXB, COMP, ADAMTSL4 and CLEC3B are shown by age (Panels A-E) across Types. Panels A-E generally show a trend for type but not age.

**Figure 2:**
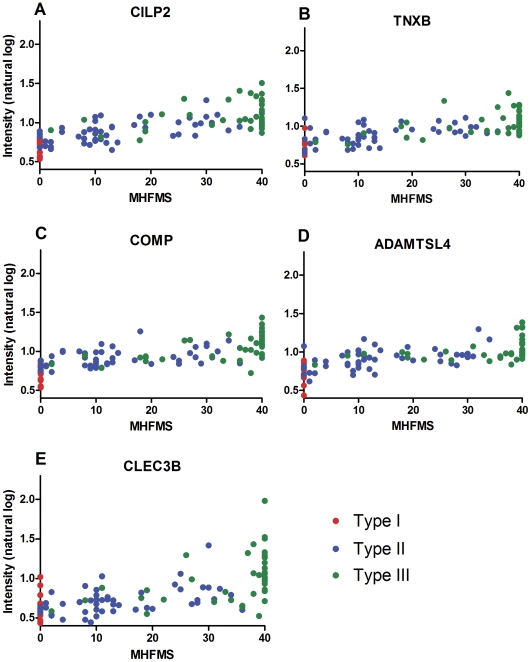
Illustration of the top 5 markers as candidate biomarkers by MFMHS. The natural log intensity of the protein abundance of CILP2, TNXB, COMP, ADAMTSL4 and CLEC3B are shown by MHFMS (Panels A-E) across Types. Panels A-E again show a trend for type and MHFMS. Panels F-J shows the box plot distribution by type.

**Figure 3:**
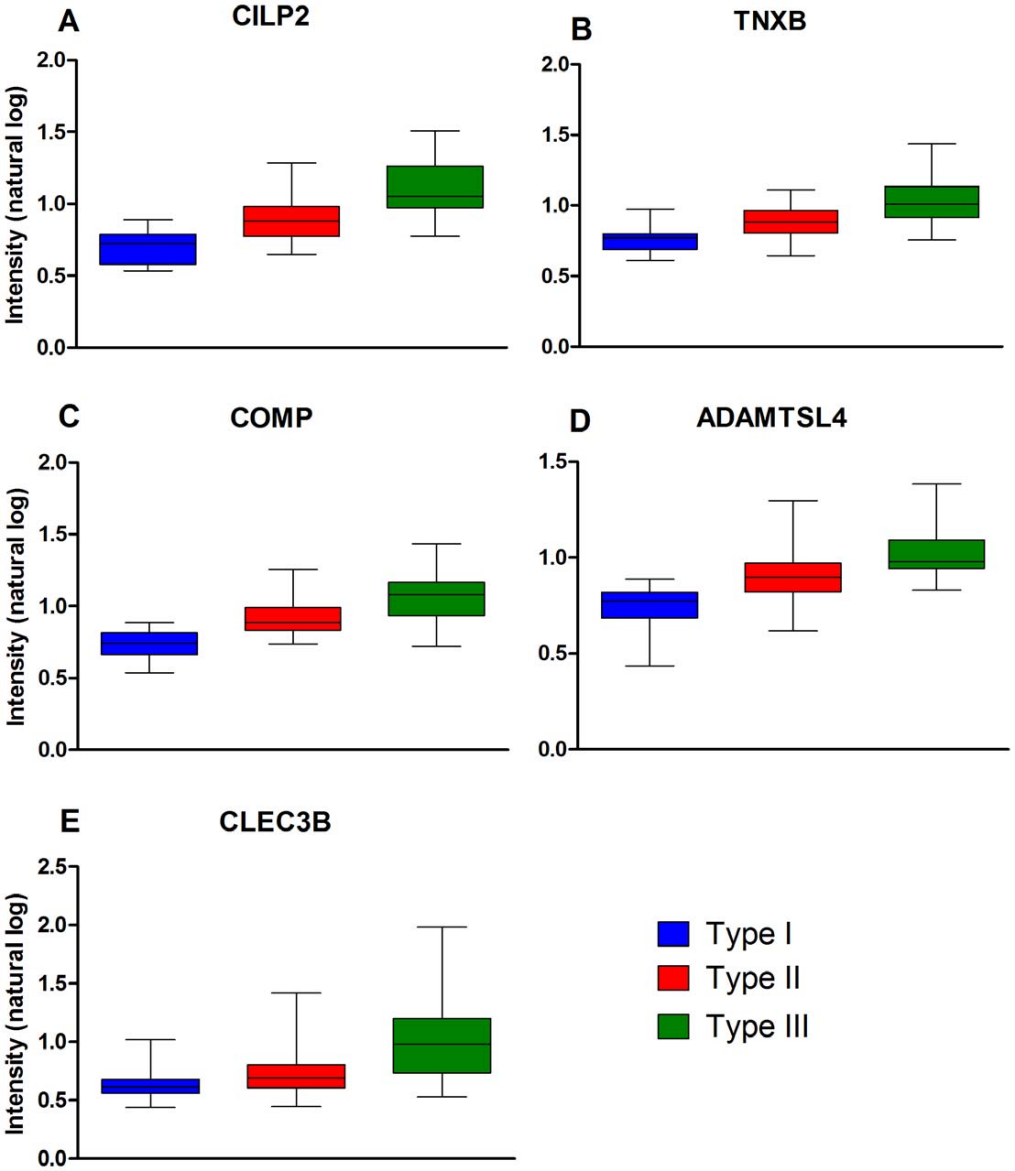
Illustration of the top 5 markers as candidate biomarkers by Type. The natural log intensity of the protein abundance of CILP2, TNXB, COMP, ADAMTSL4 and CLEC3B are shown (Panels A-E) by Type. Error bars are expressed as standard errors.

There were 701 proteins measured in plasma across 127 samples with MALDI iTRAQ platform (one sample was lost during processing). Of these, 151 were associated with at least one outcome measure. 97 plasma proteins regressed against the MHFMS. Among the top ranking candidate markers were plasma proteins that distinguished Type II from Type III subjects, Type III subjects from healthy controls, as well as Type I SMA subjects from each of the other groups. Figures 1, 2, 3 shows an example of the top 5 analytes, that discriminated between SMA types in this study. The top 20 plasma protein hits are listed in Table 4. Ninety-one percent (91%) of the proteins were measured with a coefficient of variation less than or equal to 0.2, demonstrating high measurement reproducibility. The complete list of plasma proteins that associated with the MHFMS is available through the publicly accessible database at http://neuinfo.org/bforsma.

**Table 4:** Top 20 univariate MHFMS markers across platforms.

Platform	Symbol	Description	Slope	STD	P-value	Q-value	UCL	LCL
Proteomics	CILP2	Cartilage intermediate layer protein 2	53.32	4.57	1.95E-20	1.07E-17	44.26	62.38
Proteomics	TNXB	Tenascin XB	62.91	6.09	1.66E-17	4.56E-15	50.83	74.99
Proteomics	COMP	Cartilage oligomeric matrix protein	60.78	6.04	7.02E-17	1.29E-14	48.80	72.76
Proteomics	ADAMTSL4	ADAMTSlike 4	33.91	3.87	4.84E-14	6.66E-12	26.24	41.58
Proteomics	CLEC3B	C-type lectin domain family 3, member B	56.47	6.55	9.31E-14	1.02E-11	43.49	69.46
AAA	Glu	L-Glutamic acid	−22.86	2.76	4.86E-13	9.73E-11	−28.34	−17.38
GC/MS	Glu	L-Glutamic acid	−21.10	2.57	6.29E-13	9.73E-11	−26.19	−16.01
Proteomics	TNXB	Tenascin XB	53.20	7.17	3.68E-11	3.37E-09	38.98	67.41
GC/MS	Asp	L-Aspartic-acid	−25.46	3.58	1.48E-10	1.53E-08	−32.56	−18.37
Proteomics	DPP4	Dipeptidylpeptidase 4	65.10	10.19	5.61E-09	4.41E-07	44.88	85.32
Proteomics	THBS4	Thrombospondin 4	51.46	8.28	1.15E-08	7.89E-07	35.04	67.88
GC/MS		C10∶0-fatty-acid	−7.47	1.34	2.16E-07	1.67E-05	−10.14	−4.81
AAA	Asp	L-Aspartic-acid	−17.76	3.27	3.82E-07	2.12E-05	−24.25	−11.27
Lipid		24∶1 Sphingomyelin	−7.88	1.46	4.60E-07	2.12E-05	−10.78	−4.98
FFA		C10∶0 (capric)	−6.47	1.20	4.80E-07	2.12E-05	−8.86	-4.08
Proteomics	CDH13	Cadherin 13	42.61	7.89	4.57E-07	2.79E-05	26.95	58.27
Proteomics	CRTAC1	Osteomodulin	46.61	8.80	6.95E-07	3.48E-05	29.15	64.07
Proteomics	OMD	Cartilage acidic protein 1	46.94	8.81	6.67E-07	3.48E-05	29.45	64.44
Proteomics	PEPD	Peptidase D	25.14	4.91	1.45E-06	6.63E-05	15.40	34.87
Proteomics	F13B	Coagulation factor 13 polypeptide B	41.57	8.18	1.70E-06	7.20E-05	25.35	57.79

Table is ordered by lowest to highest Q-values. Only plasma markers are represented, as these were the markers with the lowest Q-values. Unknown metabolites were removed from this summary table but the full plasma and urine metabolite and proteomic datasets are available in the Tables S3, S4, S5. AAA – Amino acid analysis; FFA – Free fatty acid; Q-VALUE – significance corrected for the effect of multiple comparisons; STD – Standard deviation; UCL – Upper 95% confidence limit; LCL – lower 95% confidence limit (on the value of slope). Slope values are given as positive and negative (-) values. *These are distinct isoforms detected by the LC/MS method.

### Plasma Metabolomics

There were 315 analytes measured across the four metabolomic platforms; 71 in the Lipid platform, 160 in the GC/MS platform, 37 in the AAA platform and 47 in the FFA platform (Table 3). Of these analytes, 169 were associated with at least one outcome measure. A total of 59 analytes regressed against the MHFMS. Table 4 shows the plasma metabolite hits in the top 20 hitlist across the four platforms, Table 5 summarizes the total number of statistically significant relationships these analytes have with all outcome measures and the complete results of plasma metabolites regression against the MHFMS is in Table S4. The complete list of plasma metabolites ordered by the number of outcomes with which they associate by univariate analysis is available through the publicly accessible database at http://neuinfo.org/bforsma.

**Table 5:** Top 20 univariate markers across all outcome measures.

Platform	Matrix	Symbol	Description	Total(36)	SMA Typestotal (12)	Disease onset total (5)	Current level of function total (17)	Respiratory support total (4)
Proteomics	Plasma	TNXB	Tenascin XB	35	12	5	11	3
Proteomics	Plasma	CILP2	Cartilage Intermediate Layer Protein 2	35	12	5	11	3
Proteomics	Plasma	COMP	Cartilage Oligomeric Protein	34	12	5	11	3
AAA	Plasma	Glu	L-glutamic acid	32	12	4	10	3
Proteomics	Plasma	CLEC3B	C-type lectin domain family 3, member B	32	11	5	9	3
Proteomics	Plasma	TNXB	Tenascin XB	32	11	5	9	3
Proteomics	Plasma	ADAMTSL4	ADAMTSlike 4	32	10	4	11	3
Proteomics	Plasma	THBS4	Thrombospondin 4	30	12	5	8	2
GC/MS	Urine		Pantothenic acid	30	11	4	8	4
Proteomics	Plasma	OMD	Osteomodullin	27	9	5	8	2
Proteomics	Plasma	LUM	Lumican	26	8	5	7	3
Proteomics	Plasma	DPP4	DPP4	26	9	4	8	2
Proteomics	Plasma	PEPD	PEPD	26	9	4	8	2
GC/MS	Plasma		C10∶0-fatty-acid	25	8	3	7	3
Proteomics	Plasma	CDH13	Cadherin 13	25	10	4	6	3
AAA	Plasma	Asp	L-aspartic acid	24	8	4	6	3
GC/MS	Urine		Inositol	24	7	4	7	3
GC/MS	Urine		Uric acid	23	8	2	7	3
AAA	Plasma	Hyp	Hypoxanthine	22	9	1	7	2
GC/MS	Plasma	Asp	L-Aspartic-acid	22	9	4	4	2

Each numeric entry indicates the number of statistical tests in which the described analyte was found to be a statistically significant biomarker when evaluated for regression against the given outcome measures or categorical characteristics: SMA Types, Disease Onset, Current Level of Function and Respiratory Support. Each outcome measure or characteristic inherently has a certain number of possible sub-categories available for pairwise statistical testing (in parentheses for each outcome or characteristic): 12 for SMA Types, 5 for Disease Onset, 15 for current level of function and 4 for Respiratory Support (see Table S1 for details). The Total denotes the sum of all outcomes for which the protein is a marker and indicates the overall strength of the relationship between the analyte and SMA values; the maximum possible number for Total is 36, as all the analytes above are also statistical significant regressors with the MHMS outcome. AAA – Amino acid analysis; FFA – Free fatty acid; Q-VALUE – significance corrected for the effect of multiple comparisons; STD – Standard deviation; UCL – Upper 95% confidence limit; LCL – lower 95% confidence limit (on the value of slope). Slope values listed in red are positive values and those in green negative values.*These are distinct isoforms detected by the LC/MS method.

### Urine Metabolomics

There were 168 metabolites measured across 123 samples with the urine GC/MS platform. Of these 95 were associated with at least one outcome measure. The full results of urine metabolites regression against the MHFMS is listed in Table S5. Univariate marker counts by outcome are listed in the publicly accessible database at http://neuinfo.org/bforsma. Some of the analytes declared markers using multiple outcomes, such as pantothenic acid, glucuronic acid (Vitamin B5) and malic acid were highest among the weaker children, whereas uric acid, glycolic acid, xanthine, hypoxanthine, inositol, allantoin and 3-methylhistidine were all decreased in abundance in the urine of weaker children. The changes in levels of metabolites could be a consequence, in part, of the specially formulated enriched diets of very weak children.

### Whole Blood Transcript Statistical Summary

Analysis of transcript data was done at both the gene expression and exon levels. A total of 22,011 transcripts and 807,038 exons were measured in the final data set, but no markers reached the threshold of significance in regression against the MHFMS. The top marker that regressed against SMA type was the NLR family apoptosis inhibitory protein NAIP. SMN exon 7 was not found to be significant against SMA type due to the relatively low abundance of SMN transcript in whole blood and limits of detection using the Affymetrix GeneChip Human Exon 1.0 ST Array. We found the mean of all probe sets to be 10 times higher than the mean of all SMN probe sets.

## Discussion

This study provides an unbiased evaluation of potential biomarkers in a well-characterized SMA cohort, carefully controlled to avoid confounding factors introduced by age and functional level. Preliminary analyses identified a rich set of more than 400 analyte markers that regressed significantly against one or more clinical outcome measures in the BforSMA study, 200 of which were against the primary outcome measure, the MHFMS. Candidate markers from this group included 97 plasma proteins, 59 plasma metabolites and 44 urine metabolites.

A key feature of this study is the combination of measures of historical (SMA type) and current function, in a cohort in which recruitment was targeted specifically to minimize the correlation between the two. SMA is unique among monogenic disorders for having its phenotype severity modified by production of a smaller quantity of an identical SMN protein produced from the highly homologous SMN2 gene present in all individuals with SMA. In monogenic neurodegenerative disorders the level of impairment can be modeled as a function of residual activity of the mutated gene and the effect of other (genetic and environmental) factors that modify its impact. Strong association of an identified analyte to MHFMS more than SMA type suggests its relationship to the factors involved in phenotype expression other than SMN, or to more downstream consequences of the debility associated with SMA type or MHFMS.

As there were no a priori assumptions as to what class of analyte (transcript, protein or metabolite) would likely generate biomarkers associated with the MHFMS, a broad set were selected to both maximize class distribution while minimizing experimental error. This approach has been used in biomarker discovery previously in models of atherosclerosis [51]–[53] and hepatotoxicity [38], [54].

### Cohort Recruitment in SMA Clinical Studies

A number of lessons were learned in the clinical phase of this project. Rapid enrollment was enhanced by a competitive process with weekly reports to participating sites that encouraged, and later constrained, recruitment to needed patient groups based upon age, SMA type and gender. Recruitment was further enhanced by the support of patient advocacy groups, and the assistance of International Spinal Muscular Atrophy Patient Registry at Indiana University to identify subjects interested in participating in the study. One important barrier to enrollment, particularly in weaker individuals with SMA Type I who were the most difficult to recruit, was the exclusion of patients taking prescription drugs. In a few subjects, blood or urine samples could not be obtained, in each case a function of difficult venous access or the limited cooperation possible from children. As expected, recruitment was slower in the winter months and around holidays. The possibility of biases against the population of individuals with SMA as a whole that were introduced by the inclusion and exclusion criteria cannot be excluded. As only children age 2 to 12 years were included, for reasons described previously, the more typical SMA Type I infants with severe weakness were largely excluded as they would not in many cases still be alive or meet the inclusion criteria. Thus the Type I population in this study is potentially biased towards the stronger and more stable subjects.

The study group identified several issues associated with sampling procedures in this study including an apparent lack of appropriate sample collection and storage materials for pediatric subjects, lack of reference standards for pediatric subjects and a paucity of data on sample handling and analytics for biomarker and pediatric studies. This is an untapped area of research that is of critical importance with increasing attention to therapeutics programs for children. For this study population the only systematic issue identified was that the smaller children were more often the ones in whom it was difficult to obtain samples. In future studies of this type using experts in venipuncture for small children may improve the rate of successful sample procurement.

### Issues Arising from Limited Range of Functional Measures

One limitation of this study is the use of a scale that may not differentiate among the lowest (“floor effect”) and highest (“ceiling effect”) functioning subject as the primary outcome measure of motor function for correlation to biomarker analyte values. The MHFMS is designed to assess function in children with Type II SMA. As a consequence, it cannot assess the variety of differences in motor function of those with SMA who are unable to sit (Type I infants, and those with SMA who once sat but have since lost this ability) or who are able to stand and walk (*i.e.* those with Type III SMA who retain this defining motor ability). Our study strategy was thus to use the MHFMS as the primary outcome measure for regression to analyte values, but to also evaluate analyte regressions to other measures of function (Table 1 and Table S1) that assess ability outside of the MHFMS range, or complement the MHFMS by assessing other motor functions not targeted by items in the MHFMS.

The restricted range of the MHFMS also introduces potential error in the strength of associations found. By design, our study cohort included subjects who scored the constrained maximum or minimum MHFMS value. Because ceiling and floor values will increase (positive) or decrease (negative) slopes of identified associations, and alter the correlation strength in an unknowable way, we conducted a *post hoc* test analysis of the primary outcome measure removing subjects having these border values. This post hoc approach, by decreasing the number of subjects, will necessarily decrease the power of the statistical models. Nonetheless, the strongest hits as ranked by Q-value were reproduced when the border-score subjects were removed from the analysis.

Given the restricted range of the MHFMS and some of the supplemental measures, it is notable that identified candidate makers that were found to discriminate between high or low functioning SMA subjects were generally consistent across all outcome measures employed in the study (MHFMS, Current Level of Function, Respiratory Support, and Feeding Method). The biological importance of these findings needs to be explored: these correlations may indicate distinct phases of disease pathogenesis, different pathological mechanisms, or even the temporal importance of SMN during early motor development. However, the very existence of markers that distinguish between Type I and Type II, Type II and Type III and between Type III and control subjects is highly encouraging of the potential to develop pharmacodynamics biomarkers from this list. Whether these candidate markers have any predictive value for different therapeutic mechanisms of action remains to be determined.

### Protein, Metabolite, and Transcript Findings

Plasma protein candidate markers were generally the most significant markers of the set in the univariate analysis against the MHFMS as well as across other outcome measures. While statistically significant metabolomics analytes were identified, candidate markers from this sample set are undergoing further post hoc evaluation to assess potential confounding effects of special diet and nutritional supplements provided to the majority of the Type I SMA subjects. If confirmed, these metabolomic associations to clinical markers of severe motor function impairment may undermine both their potential usefulness as candidate markers and enthusiasm for further developmental work of their validation.

Unexpectedly, there were no widespread changes in gene expression that correlated with disease severity or comparison of SMA to controls. We also did not observe a significant difference in SMN expression levels between SMA patients and control subjects. By using another method of transcript quantification, lower levels of SMN-FL transcripts were demonstrated for this cohort patients compared to controls (see companion paper, Crawford et al. [61]). This discrepancy is most likely due to a difference in the methodology used to quantify the levels of SMN transcripts. The absence of widespread changes in gene expression or splicing in blood suggests that the degree of reduction of the SMN protein levels in this tissue is not sufficient to cause dramatic changes on the level of gene expression or splicing, at least in peripheral blood mononuclear cells. This would be consistent with the fact that blood and other tissues of the body do not typically exhibit significant disturbances of cellular or organ function except in the Type I cohort. Limited SMN expression at the level of symptomatic SMA appears to predominantly affect motor neurons, and possibly some other neuronal types [55]. Pathologic changes have been observed in the context of extreme reduction of SMN production in every tissue that has been investigated, but the basis for the increased vulnerability of motor neurons is unknown [55]. The study did identify a decrease in the expression of the NLR family inhibitory protein (NAIP), which is consistent with the genomic deletion of SMN1 together with neighboring NAIP in SMA alleles associated with severe type [56], [57].

### The Path to Validation and Qualification and the Biological Utility of the Plasma Biomarkers

The BforSMA project is designed to be an unbiased approach to generating a dataset that can be used for biomarker identification and also post-hoc hypothesis testing. On one hand, an unbiased ascertainment of large data sets will, by design, generate false positives.

When one attempts to determine a role in disease for the statistically significant biomarkers discovered in plasma a few caveats must be emphasized. Any biological differences recorded in the plasma are likely to be downstream, and in some cases far downstream, from the original disease perturbation in SMA, at the motor neuron or neuromuscular junction. A significant perturbation from the norm, as related to a measure of motor function, can reflect any of a number of biological processes that affect bone and other connective tissues and not be indicative of a specific pathophysiological link at the motor neuron level or necessarily reflect reduced levels of SMN protein expression. From a biomechanical perspective changes in bone components are not surprising in SMA. Tendons transmit contractile muscle forces to bone. In a state of muscle weakness, such as in SMA, these forces to bone are less, especially in the long bones of the limbs in the non-ambulatory SMA types I and II. As such, bone remodeling processes, an especially important process in a growing child, are altered. Thus, a case can be made for the functional differences in bone growth remodeling between the weakest and strongest children. Cartilage intermediate layer protein 2 (CILP2), a marker with a strong association to the Hammersmith Functional Motor Score, may not have a direct relationship to motor neuron biology. However it is notable that CILP2, like other high-scoring plasma protein markers such as COMP, TNXB, THBS4, SPP1, COL2A1 to name a few, are associated with connective tissue development (cartilage matrix synthesis in the case of CILP2) and bone and joint disorders. SMA patients have been reported to have bone density losses that are correlated to age, frequent fractures, and in severe cases congenital fractures [58]. It is possible that the bone and joint protein signature present in the BforSMA study relates to secondary connective tissue sequelae in the disease. In addition, Shanmurgan et al. found that the SMN protein is a binding partner to osteoclast stimulating factor (OSF), a protein involved in osteoclast development and bone resorption which raises the possibility of a direct SMN-related perturbation in bone in SMA.

On the other hand, biomarkers like CILP2 identified in this study have the advantage of being independent of specific hypotheses about pathophysiology. While some biomarkers may initially attract attention based upon arguments arising from biological plausibility, the role of other potentially valuable biomarkers may not be immediately obvious. Much further work will be necessary to identify analytes on this list that can improve the understanding of SMN deficiency on cellular pathophysiology, or to develop a biomarker that can be valuable to the efficient performance of SMA treatment trials, whether of a specific SMN-enhancing agent or of a therapy targeting other steps of the disease cascade.

Several questions remain to be answered. Will a candidate biomarker be stable over time, and is measurement stability influenced by technical issues such as sample handling or short-term biologic factors such as diet or time of day? Will changes in candidate biomarkers track meaningful changes in impairment, such that it may be an early surrogate for the consequences of SMA as it is experienced by patients? Or will changes over time reflect other processes (*e.g.* sarcopenia) that are not related to motor function and therefore may not be informative in the domain of interest? By including a cohort of age and gender matched subjects with other motor impairments relating to localizable neurologic conditions (e.g. myopathy or cerebral palsy), a confirmatory study may help to clarify if changes in biomarkers are specific to SMA or instead relate better to downstream consequences of the disorder. The power of any one biomarker might be insufficient for use in treatment trials, but the contribution of a panel of qualified biomarkers that independently contribute to clinical assessment might be of value to explore. Experimental subjects in this cohort were all healthy at the time of enrollment and cared for at major academic centers where the care of children with SMA is a priority. Would a candidate biomarker perform as well in a larger population of children with SMA compared to those in the BforSMA study? Not all of the development need be in the clinical laboratory. Improvements in measurement of clinical function [59], better matching them to the underlying neuronal dysfunction, or extending their range so that a broader range of children with SMA can be assessed, may yield improvement in biomarker performance characteristics.

The path of a biomarker, from candidate identified by single-visit correlation to a clinical feature to becoming a qualified biomarker with well-characterized meaning, is necessarily multi-faceted and complicated. Confirmation of these observations will require that we reproduce findings in other prospectively collected samples from SMA cohorts that share features with the BforSMA population. Because the BforSMA study was a single-visit effort, to truly determine whether these candidate markers are prognostic and can change with SMA status, they must be tested over time in longitudinal assessments. Such projects are now in development with new analytic methods that can more readily be scaled to clinical research protocols.

Support and further validation of biomarkers can come from other areas, such as confirmation in SMA animal models. The assembly of biomarker networks of metabolites, proteins and transcripts based on statistical significance has the potential to be partially internally validating, as it may identify coordinated cellular or tissue physiologic strain of pathophysiologic importance not apparent from any single metabolite or platform of analysis. Further evaluation of identified candidates to understand their pathophysiologic relationship to SMN, or to the consequences of neuromuscular impairment, might be possible by further biomarker study with SMA patients in which muscle physiology outcome measures that conceptually describe the motor unit function and structure, or comparison to SMA animal models or to other disease control human populations. In particular, testing any hypothesis that a subset of these markers are primarily SMA-specific versus being consequences of secondary changes downstream of neuromuscular disease would be valuable for further study. However, it is important to emphasize that whether they are primary or secondary to SMA pathophysiology, any markers that strongly associate with SMA outcomes over time and replicate in different studies will be of great value in clinical trials and patient management.

Finally, once a subset of the markers is confirmed in other populations and prospective studies, efforts can be devised to determine if combining markers across platforms with SMA clinical characteristics could produce a multicomponent predictive model that has even stronger associations with SMA status or is possibly better able to predict outcomes or response to interventions. These types of markers have proven very powerful in the cancer field and with the emergence of new SMA drug trials, there is potential for developing information about multicomponent models for drug response and even stratify responder populations with some of these SMA candidate markers after they have been confirmed and shown to be responsive to therapy [15], [60].This BforSMA project has generated a resource of protein and metabolite candidate biomarkers for future study. The effort has taken advantage of recent technological developments that enhance our ability to measure a broad range of proteins, metabolites and transcripts from a single blood sample; a well-characterized set of SMA subjects in whom function and age are independent; and the advanced bioinformatics and biostatistical resources necessary to support the project. The data and samples generated by this effort will be an important resource for the field and future studies, with the additional value of serving as a single visit ‘test run’ for an industry-style multicenter trial for several SMA clinical sites. The full dataset from the study will be made available to all investigators in an accessible format to be used as a resource to address the many questions raised by our findings.

## Full List of Competing Interests

Dr. Finkel receives commercial research support from PTC Therapeutics, Santhera Pharmaceuticals, and Genzyme Corp. and non-profit research support from the SMA Foundation and support from the NIH/NIAMS and NIH/NINDS, accepted travel stipends as part of grants from PTC Therapeutics and the SMA Foundation, reviewed and prepared a report for Adibi legal proceeding, spends 50% of his professional time carrying out clinical studies, and serves on the medical advisory board of DuchenneConnect and Families of SMA and on the scientific advisory board of PTC Therapeutics. Dr. Crawford serves on the medical and scientific advisory boards of Families of SMA, the medical board of the Muscular Dystrophy Association, and has served as frequent ad hoc advisor to the Scientific Advisory Board of the SMA Foundation. He has received research support for clinical studies from Families of SMA, SMA Foundation, and the Ataxia Telangiectasia Children’s Project. Dr. Swoboda serves on the scientific advisory boards of Families of SMA, the Pediatric Neurotransmitter Disorders Foundation, California Stem Cell, Inc., and the Alternating Hemiplegia of Childhood Foundation (AHCF). She serves as an ad-hoc reviewer for the Muscular Dystrophy Association (MDA) and NIH. She has accepted research funds for consultation for Biomarin Pharmaceuticals and Shire, Inc. She receives or has received in the past year grant funding from Families of SMA, MDA, FightSMA, AHCF and NIH (R01-HD054599; ARRA 5-R01 HD054599-04, and 1-R01-HD69045 from NICHD). Dr. Kaufmann is an employee of the federal government and has no disclosures. Prior to August 2009, Dr. Kaufmann was an employee of Columbia University and received research support from the NIH, the SMA Foundation, PTC, Santhera Pennwest and the DoD. This report is based on Dr. Kaufmann’s work at Columbia University and is not related to the National Institutes of Health. Drs. Juhasz, X. Li, and Guo are employed by BG Medicine (BGM). BGM was paid by the sponsoring organization of this study for sample and statistical analysis. Drs. R Li and Trachtenberg are employed by New England Research Institutes (NERI). NERI was paid by the sponsoring organization of this study to coordinate this study and perform additional analyses. Ms. Forrest and Ms. Joyce were employed of the SMA Foundation during the time of the study. Dr. Chen and Dr. Kobayashi are paid employees of the SMA Foundation. Dr. Plasterer was employed by BG Medicine (BGM) from July 2001–March 2010. BGM was paid by the sponsoring organization of this study for sample and statistical analysis. The SMA Foundation has filed a patent application on aspects of this work, International Patent Application No. PCT/US2010/048675, and Ref: SMAF-005/01WO 304991-2019. A member of Dr. Finkel’s immediate family receives commercial research support from Merck Pharmaceuticals, has received license fee payments from Southern Biotechnology Associates, Upstate Pharmaceuticals, and Santa Cruz Biotechnology, receives research funding from the NIH (grant #s: AR058606, 1R21 AI078387, 1R21 AI078387-S1, 1R41 AI071927, R01 AI063623, 1U19AI082726, T32; pending grant #s: 1R21, AR059466, T32, AR059650), holds 6 patents or pending patents, contributes to other clinical research as a local co-investigator or PI in studies funded by the NIH and the UK, is the editor of Janeway Textbook of Immunology and Arthritis Research and Therapy, and devotes 33% of her professional time to clinical studies in her practice. This does not alter the authors' adherence to all the PLoS ONE policies on sharing data and materials.

## Supporting Information

Table S1 **Outcome measures and endpoints used in comparisons.** Outcome measures for this study were selected 1) to capture current functional status, 2) for anticipated use in therapeutic drug trials and 3) for use in the community. All evaluators participated in a one day training session; inter-rater reliability on the MHFMS primary outcome measure was excellent with an intra-class correlation for the total score of 0.995 (3∶1). Use of “Modified Hammersmith Motor Scale©” and instructional materials provided by Families of Spinal Muscular Atrophy.
(DOC)

Table S2 **The Coefficient of Variation (CV) for the categories of mass spectroscopy samples are listed by single and grouped deciles.**
(DOC)

Table S3 **Plasma proteomic univariate analysis against the MHFMS.** Q-VALUE – significance corrected for the effect of multiple comparisons; STD – Standard deviation; UCL – Upper 95% confidence limit; LCL – lower 95% confidence limit (on the value of slope); NA – Analytes could not be identified, no assessment made.
(DOC)

Table S4 **Plasma metabolite analysis against the MHFMS.** Analytes described as “unknown” indicate metabolites that could not be identified and there was low confidence of predicting the correct analyte given the acquired structural information; AAA – Amino acid analysis; FFA – Free fatty acid; Q-VALUE – significance corrected for the effect of multiple comparisons; STD – Standard deviation; UCL – Upper 95% confidence limit; LCL – lower 95% confidence limit (on the value of slope); NA – Analytes could not be identified, no assessment made.
(DOC)

Table S5 **Urine metabolite analysis against the MHFMS.** Analytes with XXXX_uk## formats indicate metabolites that could not be identified and there was low confidence of predicting the correct analyte given the acquired structural information; Q-VALUE – significance corrected for the effect of multiple comparisons; STD – Standard deviation; UCL – Upper 95% confidence limit; LCL – lower 95% confidence limit (on the value of slope); NA – Analytes could not be identified, no assessment made.
(DOC)

Appendix S1 **BforSMA Final Protocol NERI IRB-approved word version.**
(DOC)

Appendix S2 **Medication history for all subjects.**
(DOC)

List S1 **Members of the BforSMA Trial Group.**
(DOC)
