# Supplementary material for: Candidate Proteins, Metabolites and Transcripts in the Biomarkers for Spinal Muscular Atrophy (BforSMA) Clinical Study
Source: PLoS One. 2012 Apr 27;7(4):e35462. doi: 10.1371/journal.pone.0035462 (PMC3338723; doi:10.1371/journal.pone.0035462)
Supplement: Table S1 — Outcome measures and endpoints used in comparisons. Outcome measures for this study were selected 1) to capture current functional status, 2) for anticipated use in therapeutic drug trials and 3) for use in the community. All evaluators participated in a one day training session; inter-rater reliability on the MHFMS primary outcome measure was excellent with an intra-class correlation for the total score of 0.995 (3∶1). Use of “Modified Hammersmith Motor Scale©” and instructional materials provided by Families of Spinal Muscular Atrophy. (DOC) [file pone.0035462.s001.doc]

**Supplementary Table S1. Outcome measures and endpoints used in comparisons**

| Outcome Measure | Variable TYPE | Notes |
| --- | --- | --- |
| MHFMS* | Continuous | Ordinal scale of 20 test items |
| SMA type | Categorical | Divided into 2, 3, or 4 categories (Type I, Type II, Type III, Carrier) |
| Current Level of Function | Categorical | Parental report; 3 (non-sitter, sitter and/or stander, walker) or 5 categories (Type I, Type II non-rolling, Type II rolling, Type III non-stair climbing, Type III stair-climbing) |
| Age at Disease Onset | Continuous or categorical | Parental report; 3 (0-6 months, 7-17 months, >18 months) or 8 categories (0-3 months, 4-6 months, 7-11 months, 12-17 months, 18-23 months, 24-35 months, 3-5 years, 6-12 years) |
| Body Mass Index z-Score | Continuous | Nutritional Status |
| Feeding Method | Categorical | Functional questionnaire: 3 categories (modified intake/G-tube fed, solid food) |
| Respiratory Support | Categorical | Functional questionnaire: 2 categories (Cough assist, BiPAP, None) |
| Pulmonary Function | Continuous | Percent predicted FVC in those > 5 years of age |
| 10 Meter Walk Time | Continuous | Ambulatory subjects |
| SMN protein | Continuous | Measured in PBMCs by ELISA |
| SMN2 copy number | Continuous or categorical | Measured using 2 different techniques |
| Smn transcript levels | Continuous | Measured by absolute quantification methods |
