# Supplementary material for: Candidate Proteins, Metabolites and Transcripts in the Biomarkers for Spinal Muscular Atrophy (BforSMA) Clinical Study
Source: PLoS One. 2012 Apr 27;7(4):e35462. doi: 10.1371/journal.pone.0035462 (PMC3338723; doi:10.1371/journal.pone.0035462)
Supplement: Table S2 — The Coefficient of Variation (CV) for the categories of mass spectroscopy samples are listed by single and grouped deciles. (DOC) [file pone.0035462.s002.doc]

**Supplemental Table S2. The Coefficient of Variation (CV) for the categories of mass spectroscopy samples are listed by single and grouped deciles.**

| CV Range | % of measured analytes | | | | | |
| --- | --- | --- | --- | --- | --- | --- |
|  | Plasma Proteomics | Plasma lipid Profiling | Plasma Amino Acid Analysis | Plasma Free Fatty Acid Analysis | Plasma GC/MS | Urine GC/MS |
| 0%  to 10% | 20.1% | 5.6% | 81.1% | 68.3% | 52.5% | 85.7% |
| 10% to 20% | 71.2% | 46.5% | 16.2% | 22.0% | 30.0% | 11.3% |
| 20% to 30% | 7.8% | 29.6% | 0.0% | 7.3% | 11.3% | 2.4% |
| 30% to 50% | 0.7% | 18.3% | 0.0% | 2.4% | 5.0% | 0.6% |
| >50% | 0.2% | 0.0% | 2.7% | 0.0% | 1.3% | 0.0% |
