# Supplementary material for: Candidate Proteins, Metabolites and Transcripts in the Biomarkers for Spinal Muscular Atrophy (BforSMA) Clinical Study
Source: PLoS One. 2012 Apr 27;7(4):e35462. doi: 10.1371/journal.pone.0035462 (PMC3338723; doi:10.1371/journal.pone.0035462)
Supplement: Table S3 — Plasma proteomic univariate analysis against the MHFMS. Q-VALUE – significance corrected for the effect of multiple comparisons; STD – Standard deviation; UCL – Upper 95% confidence limit; LCL – lower 95% confidence limit (on the value of slope); NA – Analytes could not be identified, no assessment made. (DOC) [file pone.0035462.s003.doc]

**Supplementary Table S3. Plasma proteomic univariate analysis against the MHFMS**

| **Analyte Name** | **EGID** | **Slope** | **Std** | **LCL** | **UCL** | **P-value** | **Q-value** |
| --- | --- | --- | --- | --- | --- | --- | --- |
| CILP2 | 148113 | 53.32 | 4.57 | 44.26 | 62.38 | 1.95E-20 | 1.07E-17 |
| TNXB | 7148 | 62.91 | 6.09 | 50.83 | 74.99 | 1.66E-17 | 4.56E-15 |
| COMP | 1311 | 60.78 | 6.04 | 48.80 | 72.76 | 7.02E-17 | 1.29E-14 |
| ADAMTSL4 | 54507 | 33.91 | 3.87 | 26.24 | 41.58 | 4.84E-14 | 6.66E-12 |
| CLEC3B | 7123 | 56.47 | 6.55 | 43.49 | 69.46 | 9.31E-14 | 1.02E-11 |
| TNXB | 7148 | 53.20 | 7.17 | 38.98 | 67.41 | 3.68E-11 | 3.37E-09 |
| DPP4 | 1803 | 65.10 | 10.19 | 44.88 | 85.32 | 5.61E-09 | 4.41E-07 |
| THBS4 | 7060 | 51.46 | 8.28 | 35.04 | 67.88 | 1.15E-08 | 7.89E-07 |
| CDH13 | 1012 | 42.61 | 7.89 | 26.95 | 58.27 | 4.57E-07 | 2.79E-05 |
| OMD | 4958 | 46.94 | 8.81 | 29.45 | 64.44 | 6.67E-07 | 3.48E-05 |
| CRTAC1 | 55118 | 46.61 | 8.80 | 29.15 | 64.07 | 6.95E-07 | 3.48E-05 |
| PEPD | 5184 | 25.14 | 4.91 | 15.40 | 34.87 | 1.45E-06 | 6.63E-05 |
| F13B | 2165 | 41.57 | 8.18 | 25.35 | 57.79 | 1.70E-06 | 7.20E-05 |
| CD93 | 22918 | 44.94 | 9.59 | 25.92 | 63.96 | 8.82E-06 | 0.000347 |
| LUM | 4060 | 34.86 | 7.59 | 19.81 | 49.91 | 1.25E-05 | 0.000459 |
| APCS | 325 | -20.60 | 4.54 | -29.61 | -11.59 | 1.59E-05 | 0.000548 |
| COL2A1 | 1280 | 30.28 | 6.65 | 17.06 | 43.50 | 1.70E-05 | 0.000549 |
| NOV | 4856 | 26.76 | 5.88 | 15.05 | 38.47 | 1.93E-05 | 0.00059 |
|  |  | -54.59 | 12.25 | -78.88 | -30.30 | 2.15E-05 | 0.000622 |
| COL6A3 | 1293 | 39.01 | 9.02 | 21.11 | 56.90 | 3.62E-05 | 0.000995 |
| CRP | 1401 | -8.34 | 2.01 | -12.33 | -4.35 | 7.16E-05 | 0.001874 |
| VTN | 7448 | -27.84 | 6.77 | -41.28 | -14.41 | 8.03E-05 | 0.002007 |
| VTN | 7448 | -28.26 | 7.10 | -42.35 | -14.17 | 0.00013 | 0.003089 |
| GSN | 2934 | 37.02 | 9.33 | 18.50 | 55.54 | 0.000137 | 0.003089 |
| CFI | 3426 | -31.39 | 7.94 | -47.14 | -15.64 | 0.000143 | 0.003089 |
| HBB | 3043 | -11.39 | 2.89 | -17.12 | -5.67 | 0.000146 | 0.003089 |
| ALPL | 249 | 30.34 | 7.79 | 14.87 | 45.81 | 0.000185 | 0.003768 |
|  | 337 | 20.10 | 5.23 | 9.74 | 30.47 | 0.00021 | 0.004117 |
| NEO1 | 4756 | 46.08 | 12.12 | 22.03 | 70.13 | 0.000248 | 0.004366 |
| LRG1 | 116844 | -25.83 | 6.76 | -39.25 | -12.40 | 0.000239 | 0.004366 |
| FCGR3A | 2214 | 23.66 | 6.23 | 11.30 | 36.02 | 0.000254 | 0.004366 |
|  |  | -10.03 | 2.63 | -15.25 | -4.80 | 0.000245 | 0.004366 |
| F13A1 | 2162 | 34.95 | 9.25 | 16.59 | 53.30 | 0.000268 | 0.004469 |
| RPS27A | 6233 | -18.30 | 4.95 | -28.13 | -8.47 | 0.000368 | 0.005949 |
| CTSD | 1509 | -31.28 | 8.55 | -48.24 | -14.33 | 0.000403 | 0.006155 |
| C2 | 717 | -49.00 | 13.38 | -75.55 | -22.46 | 0.000401 | 0.006155 |
| IGFBP6 | 3489 | 40.27 | 11.20 | 18.05 | 62.49 | 0.000508 | 0.007546 |
| INHBC | 3626 | -26.18 | 7.35 | -40.76 | -11.60 | 0.000563 | 0.008151 |
| NCAM1 | 4684 | 62.52 | 17.97 | 26.87 | 98.17 | 0.000744 | 0.010496 |
| CP | 1356 | -14.28 | 4.14 | -22.49 | -6.07 | 0.000818 | 0.011249 |
| CKM | 1158 | 17.12 | 4.94 | 7.30 | 26.95 | 0.00084 | 0.011265 |
| AGA | 175 | -29.81 | 8.83 | -47.34 | -12.28 | 0.001062 | 0.013906 |
| C9 | 735 | -10.80 | 3.23 | -17.20 | -4.40 | 0.001152 | 0.014395 |
| LRG1 | 116844 | -20.08 | 6.00 | -31.99 | -8.18 | 0.001148 | 0.014395 |
| SPP1 | 6696 | 33.02 | 10.03 | 13.12 | 52.92 | 0.001373 | 0.016779 |
| HBA1 | 3039 | -10.18 | 3.10 | -16.33 | -4.03 | 0.001408 | 0.016838 |
| PRG4 | 10216 | -27.27 | 8.40 | -43.99 | -10.56 | 0.001688 | 0.019748 |
| MRC2 | 9902 | 25.58 | 7.99 | 9.73 | 41.43 | 0.001842 | 0.021104 |
| ISLR | 3671 | 30.09 | 9.41 | 11.40 | 48.79 | 0.001916 | 0.02151 |
| QSOX1 | 5768 | 54.96 | 17.68 | 19.89 | 90.04 | 0.002444 | 0.026885 |
| CA2 | 760 | -9.70 | 3.16 | -15.97 | -3.44 | 0.002743 | 0.029013 |
| CST6 | 1474 | -25.87 | 8.36 | -42.50 | -9.23 | 0.002721 | 0.029013 |
| PGK1 | 5230 | -22.44 | 7.27 | -37.13 | -7.75 | 0.003664 | 0.038025 |
| PROC | 5624 | -24.76 | 8.46 | -41.55 | -7.97 | 0.00424 | 0.043182 |
| SERPINA10 | 51156 | -22.30 | 7.72 | -37.61 | -6.99 | 0.004718 | 0.045527 |
| S100A4 | 6275 | -16.92 | 5.82 | -28.50 | -5.35 | 0.004687 | 0.045527 |
| PARK7 | 11315 | -17.84 | 6.13 | -30.04 | -5.65 | 0.004639 | 0.045527 |
| ENG | 2022 | 35.66 | 12.44 | 10.98 | 60.35 | 0.00507 | 0.04808 |
| ORM2 | 5005 | -8.08 | 2.83 | -13.71 | -2.46 | 0.005267 | 0.049103 |
| VCAM1 | 7412 | 35.56 | 12.94 | 9.89 | 61.23 | 0.0071 | 0.064015 |
| MET | 4233 | 30.97 | 11.20 | 8.67 | 53.27 | 0.007097 | 0.064015 |
| FAP | 2191 | 25.20 | 8.81 | 7.21 | 43.20 | 0.007626 | 0.067648 |
| CFL1 | 1072 | -12.16 | 4.46 | -21.04 | -3.28 | 0.007901 | 0.06898 |
| PGCP | 10404 | 22.96 | 8.51 | 6.07 | 39.86 | 0.00825 | 0.070249 |
| PEBP4 | 157310 | 27.34 | 9.88 | 7.42 | 47.26 | 0.008302 | 0.070249 |
| NID1 | 4811 | -25.56 | 9.50 | -44.51 | -6.61 | 0.008913 | 0.070831 |
| AFM | 173 | -22.46 | 8.48 | -39.28 | -5.63 | 0.009401 | 0.070831 |
| PF4 | 5196 | -10.99 | 4.13 | -19.21 | -2.77 | 0.009364 | 0.070831 |
| VNN1 | 8876 | -14.87 | 5.55 | -25.89 | -3.85 | 0.008676 | 0.070831 |
| HP | 3240 | -10.43 | 3.92 | -18.22 | -2.65 | 0.009079 | 0.070831 |
| PTPRG | 5793 | 24.56 | 9.25 | 6.22 | 42.91 | 0.009193 | 0.070831 |
| MB | 4151 | 21.29 | 7.91 | 5.54 | 37.04 | 0.008675 | 0.070831 |
| CNDP1 | 84735 | -13.01 | 4.86 | -22.66 | -3.35 | 0.008795 | 0.070831 |
| CA1 | 759 | -9.07 | 3.46 | -15.93 | -2.22 | 0.009991 | 0.074257 |
| SHBG | 6462 | 9.53 | 3.64 | 2.31 | 16.74 | 0.010164 | 0.074539 |
| RCTPI1 | 7167 | -9.18 | 3.52 | -16.17 | -2.18 | 0.010632 | 0.076939 |
| S100A8 | 6279 | -14.12 | 5.47 | -24.98 | -3.27 | 0.011327 | 0.080148 |
| GSS | 2937 | 25.61 | 9.71 | 6.06 | 45.15 | 0.011367 | 0.080148 |
| TAGLN2 | 8407 | -7.73 | 3.02 | -13.72 | -1.74 | 0.011928 | 0.082736 |
| DAG1 | 1605 | 38.88 | 14.83 | 8.98 | 68.78 | 0.012034 | 0.082736 |
| S100A9 | 6280 | -11.29 | 4.44 | -20.09 | -2.50 | 0.012387 | 0.084107 |
| GAPDH | 2597 | -9.18 | 3.63 | -16.38 | -1.99 | 0.012892 | 0.084409 |
| ACTN1 | 87 | -10.73 | 4.12 | -19.05 | -2.41 | 0.012707 | 0.084409 |
| CNTN4 | 152330 | 30.62 | 12.07 | 6.67 | 54.57 | 0.012745 | 0.084409 |
| AOC3 | 8639 | 27.40 | 10.90 | 5.77 | 49.03 | 0.013559 | 0.085767 |
| NP | 4860 | -11.30 | 4.50 | -20.24 | -2.37 | 0.013723 | 0.085767 |
| CACNA2D1 | 781 | 21.17 | 8.37 | 4.49 | 37.84 | 0.01359 | 0.085767 |
| TXN | 7295 | -17.46 | 6.90 | -31.20 | -3.71 | 0.013493 | 0.085767 |
| PROCR | 10544 | 14.32 | 5.72 | 2.96 | 25.68 | 0.013973 | 0.086349 |
| PRDX2 | 7001 | -8.93 | 3.59 | -16.05 | -1.81 | 0.014516 | 0.088711 |
| SOD1 | 6647 | -10.74 | 4.32 | -19.33 | -2.15 | 0.014815 | 0.08954 |
| CSF1R | 1436 | 27.98 | 11.34 | 5.48 | 50.47 | 0.015298 | 0.091152 |
| CHAD | 1101 | 37.05 | 14.65 | 7.45 | 66.64 | 0.015413 | 0.091152 |
| PRDX6 | 9588 | -8.52 | 3.48 | -15.43 | -1.61 | 0.0162 | 0.093791 |
| ORM1 | 5004 | -5.97 | 2.44 | -10.80 | -1.13 | 0.016071 | 0.093791 |
| MMRN2 | 79812 | 20.30 | 8.38 | 3.67 | 36.93 | 0.01721 | 0.098599 |
| SERPIND1 | 3053 | -20.11 | 8.32 | -36.62 | -3.60 | 0.017471 | 0.09906 |
| LCP1 | 3936 | 29.52 | 12.30 | 5.12 | 53.92 | 0.018209 | 0.102191 |
|  |  | 27.48 | 11.58 | 4.51 | 50.46 | 0.019553 | 0.108626 |
|  |  | -9.35 | 3.95 | -17.19 | -1.51 | 0.019961 | 0.109786 |
| THBS1 | 7057 | -15.21 | 6.47 | -28.05 | -2.37 | 0.020696 | 0.1127 |
| CAT | 847 | -11.61 | 4.95 | -21.42 | -1.79 | 0.021015 | 0.113314 |
| TLN1 | 7094 | -9.96 | 4.21 | -18.38 | -1.54 | 0.021241 | 0.113424 |
| PDIA3 | 2923 | -18.04 | 7.76 | -33.44 | -2.63 | 0.022201 | 0.114177 |
| SPINK1 | 6690 | -15.05 | 6.44 | -27.89 | -2.21 | 0.022213 | 0.114177 |
|  |  | -6.07 | 2.61 | -11.25 | -0.89 | 0.022074 | 0.114177 |
| IGKV4-1 | 28908 | -14.93 | 6.40 | -27.62 | -2.23 | 0.021644 | 0.114177 |
| COL6A1 | 1291 | 25.44 | 11.06 | 3.49 | 47.39 | 0.023527 | 0.119814 |
| SELENBP1 | 8991 | -11.13 | 4.86 | -20.79 | -1.48 | 0.024288 | 0.122555 |
| IGFBP7 | 3490 | 23.39 | 10.34 | 2.85 | 43.92 | 0.026039 | 0.130193 |
| CDH2 | 1000 | -21.94 | 9.71 | -41.24 | -2.64 | 0.026373 | 0.130679 |
| F9 | 2158 | -26.29 | 11.95 | -49.99 | -2.59 | 0.030054 | 0.147588 |
| HSPG2 | 3339 | 39.17 | 17.93 | 3.61 | 74.74 | 0.031213 | 0.15192 |
| PPIA | 5478 | -8.48 | 3.90 | -16.21 | -0.74 | 0.03201 | 0.153382 |
| FKBP1A | 2280 | -12.81 | 5.88 | -24.50 | -1.12 | 0.032071 | 0.153382 |
| PFN1 | 5216 | -7.05 | 3.25 | -13.49 | -0.60 | 0.032375 | 0.153501 |
| SELL | 6402 | 30.64 | 14.21 | 2.46 | 58.83 | 0.033391 | 0.155177 |
| ENO1 | 2023 | -9.96 | 4.62 | -19.12 | -0.79 | 0.033575 | 0.155177 |
| SERPINA6 | 866 | 22.72 | 10.54 | 1.81 | 43.62 | 0.03346 | 0.155177 |
| YWHAZ | 7534 | -6.28 | 2.93 | -12.09 | -0.47 | 0.034458 | 0.156627 |
| ALAD | 210 | -14.19 | 6.50 | -27.29 | -1.10 | 0.034327 | 0.156627 |
|  |  | -6.04 | 2.84 | -11.67 | -0.41 | 0.035608 | 0.160527 |
| NME1 | 4830 | -17.26 | 7.89 | -33.34 | -1.19 | 0.036136 | 0.161583 |
| EPHA4 | 2043 | 19.10 | 9.05 | 1.14 | 37.06 | 0.037397 | 0.165875 |
| FGA | 2243 | -10.21 | 4.90 | -19.92 | -0.50 | 0.039497 | 0.173788 |
| COL1A2 | 1278 | 22.06 | 10.62 | 0.98 | 43.13 | 0.040422 | 0.175058 |
|  |  | -8.30 | 3.99 | -16.23 | -0.38 | 0.040247 | 0.175058 |
| ELF1 | 1997 | -16.51 | 7.90 | -32.30 | -0.72 | 0.040748 | 0.17509 |
| GM2A | 2760 | -24.14 | 11.38 | -47.27 | -1.01 | 0.041328 | 0.176206 |
| PZP | 5858 | -10.24 | 4.98 | -20.11 | -0.37 | 0.042167 | 0.178398 |
| COL18A1 | 80781 | 20.31 | 9.89 | 0.68 | 39.94 | 0.042756 | 0.179511 |
| HBB | 3043 | -10.12 | 4.83 | -19.92 | -0.31 | 0.043484 | 0.181184 |
| PPBP | 5473 | -4.97 | 2.50 | -9.92 | -0.01 | 0.049375 | 0.204184 |
| MGP | 4256 | 33.01 | 16.15 | 0.03 | 65.99 | 0.049812 | 0.204452 |
| ITIH2 | 3698 | 13.35 | 6.75 | -0.04 | 26.73 | 0.050661 | 0.204878 |
| CFD | 1675 | -25.31 | 12.78 | -50.66 | 0.03 | 0.050303 | 0.204878 |
| SH3BGRL | 6451 | -18.63 | 9.23 | -37.37 | 0.10 | 0.051214 | 0.205602 |
| PI16 | 221476 | 19.99 | 10.19 | -0.23 | 40.21 | 0.052658 | 0.209643 |
| FGG | 2266 | -10.18 | 5.20 | -20.50 | 0.13 | 0.052982 | 0.209643 |
| AMBP | 259 | -11.61 | 5.95 | -23.41 | 0.19 | 0.053672 | 0.210854 |
| GNPTG | 84572 | -19.03 | 9.77 | -38.41 | 0.36 | 0.054263 | 0.211665 |
| SH3BGRL3 | 83442 | -11.67 | 5.89 | -23.61 | 0.27 | 0.055044 | 0.213199 |
| PCSK9 | 255738 | -14.77 | 7.64 | -29.92 | 0.38 | 0.055925 | 0.213602 |
| S100A6 | 6277 | -12.75 | 6.54 | -25.83 | 0.33 | 0.055917 | 0.213602 |
| DDT | 1652 | -20.98 | 10.74 | -42.58 | 0.62 | 0.056678 | 0.214984 |
| TMSL3 | 7117 | -8.22 | 4.21 | -16.70 | 0.26 | 0.057112 | 0.215147 |
| CHGA | 1113 | -25.01 | 12.72 | -50.87 | 0.85 | 0.057543 | 0.215299 |
| TPM4 | 7171 | -3.80 | 2.00 | -7.76 | 0.17 | 0.060177 | 0.22363 |
| LBP | 3929 | -25.66 | 12.93 | -52.63 | 1.32 | 0.061125 | 0.225629 |
| CDH5 | 1003 | 25.35 | 13.53 | -1.49 | 52.20 | 0.06392 | 0.229778 |
| BPGM | 669 | -8.26 | 4.38 | -16.96 | 0.45 | 0.062778 | 0.229778 |
| B3GNT8 | 374907 | 21.78 | 11.58 | -1.26 | 44.82 | 0.063557 | 0.229778 |
| ITGA7 | 3679 | 25.23 | 13.03 | -1.47 | 51.93 | 0.063101 | 0.229778 |
| FGB | 2244 | -8.09 | 4.38 | -16.79 | 0.60 | 0.067823 | 0.239121 |
| MASP1 | 5648 | -19.78 | 10.71 | -41.03 | 1.47 | 0.067774 | 0.239121 |
| PSME2 | 5721 | -16.28 | 8.44 | -33.83 | 1.27 | 0.067387 | 0.239121 |
| RHOXF1 | 158800 | 18.17 | 9.80 | -1.47 | 37.81 | 0.069036 | 0.241844 |
| ITGB1 | 3688 | 19.14 | 10.43 | -1.63 | 39.92 | 0.070361 | 0.244927 |
| C2 | 717 | -4.38 | 2.41 | -9.16 | 0.40 | 0.072117 | 0.24946 |
| NOTCH3 | 4854 | 13.69 | 7.50 | -1.35 | 28.74 | 0.073615 | 0.253051 |
| SHBG | 6462 | 10.14 | 5.69 | -1.14 | 21.43 | 0.077583 | 0.265035 |
| TTR | 7276 | -2.86 | 1.61 | -6.05 | 0.33 | 0.078133 | 0.265265 |
| MMP9 | 4318 | -15.99 | 9.00 | -33.92 | 1.93 | 0.079566 | 0.268474 |
| CTBS | 1486 | -21.74 | 12.45 | -46.46 | 2.98 | 0.084031 | 0.279993 |
| SERPING1 | 710 | 14.38 | 8.23 | -1.95 | 30.72 | 0.083773 | 0.279993 |
| P4HB | 5034 | -18.16 | 10.42 | -38.83 | 2.52 | 0.084507 | 0.279993 |
| CR2 | 1380 | 15.36 | 8.85 | -2.24 | 32.96 | 0.086229 | 0.280628 |
| TIMP2 | 7077 | 20.86 | 12.03 | -3.02 | 44.74 | 0.086156 | 0.280628 |
| SAA1 | 6288 | -3.53 | 2.03 | -7.57 | 0.50 | 0.085527 | 0.280628 |
| COL1A1 | 1277 | 18.42 | 10.68 | -2.76 | 39.60 | 0.087599 | 0.283407 |
| CNTN1 | 1272 | -27.47 | 16.00 | -59.20 | 4.27 | 0.089034 | 0.286367 |
| CBLN4 | 140689 | 17.19 | 10.09 | -2.85 | 37.22 | 0.091846 | 0.293694 |
| IGFBP5 | 3488 | -15.99 | 9.47 | -34.77 | 2.79 | 0.094285 | 0.299749 |
| IGJ | 3512 | 17.37 | 10.32 | -3.10 | 37.84 | 0.095474 | 0.301787 |
| WARS | 7453 | 27.51 | 15.91 | -5.26 | 60.28 | 0.096122 | 0.302099 |
| COL6A3 | 1293 | 19.68 | 11.78 | -3.71 | 43.08 | 0.098172 | 0.306789 |
| APOF | 319 | 34.52 | 20.10 | -7.28 | 76.33 | 0.10064 | 0.310965 |
| SAA4 | 6291 | -18.16 | 10.93 | -39.92 | 3.60 | 0.100614 | 0.310965 |
| FAH | 2184 | -13.38 | 8.12 | -29.56 | 2.81 | 0.103853 | 0.31669 |
| C3 | 718 | 12.17 | 7.43 | -2.58 | 26.91 | 0.104795 | 0.31669 |
| EIF5A | 1984 | -14.49 | 8.45 | -32.24 | 3.26 | 0.103471 | 0.31669 |
| LILRA3 | 11026 | 10.61 | 6.47 | -2.24 | 23.46 | 0.104516 | 0.31669 |
| PLXNB2 | 23654 | -18.54 | 11.30 | -41.21 | 4.13 | 0.106826 | 0.321061 |
| PLTP | 5360 | 17.05 | 10.55 | -3.89 | 37.99 | 0.109343 | 0.326841 |
| THBS1 | 7057 | -7.82 | 4.85 | -17.44 | 1.81 | 0.110206 | 0.327491 |
| FGFR4 | 2264 | -29.90 | 17.99 | -67.21 | 7.41 | 0.110752 | 0.327491 |
| A2M | 2 | -12.21 | 7.66 | -27.40 | 2.98 | 0.113996 | 0.335283 |
| NRP1 | 8829 | 28.08 | 17.68 | -7.00 | 63.17 | 0.11539 | 0.336964 |
| FGA | 2243 | -4.06 | 2.56 | -9.13 | 1.02 | 0.115793 | 0.336964 |
| CALR | 811 | -17.39 | 10.99 | -39.26 | 4.48 | 0.117438 | 0.339952 |
| HEG1 | 57493 | 11.95 | 7.58 | -3.13 | 27.02 | 0.118867 | 0.340505 |
| LOC442497 | 442497 | 18.95 | 12.03 | -4.96 | 42.87 | 0.118849 | 0.340505 |
| NOTCH2 | 4853 | 13.99 | 8.89 | -3.71 | 31.69 | 0.119621 | 0.340888 |
| PKM2 | 5315 | 12.22 | 7.83 | -3.36 | 27.80 | 0.122601 | 0.344985 |
| ROBO4 | 54538 | 15.77 | 10.13 | -4.34 | 35.89 | 0.122767 | 0.344985 |
| SPARCL1 | 8404 | 17.07 | 10.97 | -4.70 | 38.83 | 0.12294 | 0.344985 |
| C5 | 727 | 13.46 | 8.72 | -3.84 | 30.76 | 0.125915 | 0.35154 |
| GPX3 | 2878 | 13.13 | 8.65 | -4.02 | 30.29 | 0.131885 | 0.36088 |
| PROS1 | 5627 | -23.09 | 15.17 | -53.19 | 7.01 | 0.131203 | 0.36088 |
| GP1BA | 2811 | -17.74 | 11.64 | -40.83 | 5.36 | 0.13081 | 0.36088 |
| PRKCSH | 5589 | -26.34 | 17.10 | -60.90 | 8.21 | 0.131244 | 0.36088 |
| APP | 351 | -9.41 | 6.20 | -21.73 | 2.92 | 0.132986 | 0.36209 |
| HGFAC | 3083 | 9.68 | 6.45 | -3.10 | 22.47 | 0.136132 | 0.36883 |
|  |  | 19.00 | 12.73 | -6.34 | 44.34 | 0.139558 | 0.37626 |
| GC | 2638 | -10.53 | 7.09 | -24.59 | 3.53 | 0.140592 | 0.377197 |
| APOH | 350 | -13.90 | 9.45 | -32.76 | 4.96 | 0.145845 | 0.389392 |
| IL6ST | 3572 | 18.03 | 12.42 | -6.62 | 42.69 | 0.149794 | 0.397692 |
| AMBP | 259 | 9.45 | 6.52 | -3.49 | 22.40 | 0.1504 | 0.397692 |
| SRGN | 5552 | -16.10 | 11.23 | -38.51 | 6.31 | 0.15637 | 0.411499 |
|  |  | 9.19 | 6.45 | -3.61 | 21.99 | 0.157378 | 0.412182 |
| DPEP2 | 64174 | -17.85 | 12.65 | -42.96 | 7.26 | 0.161569 | 0.421152 |
| MDH1 | 4190 | -7.18 | 5.13 | -17.38 | 3.03 | 0.165648 | 0.428391 |
| APOL1 | 8542 | -8.38 | 6.00 | -20.30 | 3.54 | 0.165904 | 0.428391 |
| POSTN | 10631 | 12.90 | 9.29 | -5.53 | 31.33 | 0.167986 | 0.43174 |
| HSPA8 | 3312 | -12.04 | 8.64 | -29.43 | 5.36 | 0.170346 | 0.43577 |
| MCAM | 4162 | 21.88 | 15.92 | -9.69 | 53.45 | 0.172237 | 0.43761 |
| PTPRF | 5792 | -18.49 | 13.46 | -45.22 | 8.24 | 0.172808 | 0.43761 |
| HPR | 3250 | -7.78 | 5.67 | -19.05 | 3.49 | 0.173453 | 0.43761 |
| PTPRM | 5797 | 19.25 | 14.20 | -9.18 | 47.68 | 0.180602 | 0.453567 |
| PVR | 5817 | 14.94 | 11.12 | -7.13 | 37.00 | 0.182285 | 0.455711 |
| VCL | 7414 | -6.64 | 5.00 | -16.56 | 3.28 | 0.187066 | 0.465252 |
| ECM1 | 1893 | 15.13 | 11.41 | -7.50 | 37.77 | 0.187793 | 0.465252 |
| GSTO1 | 9446 | -6.29 | 4.78 | -15.78 | 3.19 | 0.190933 | 0.470912 |
| NPC2 | 10577 | -18.79 | 14.11 | -47.53 | 9.94 | 0.192178 | 0.471866 |
| HSPA5 | 3309 | -20.52 | 15.76 | -51.80 | 10.75 | 0.195891 | 0.474626 |
| SERPINA1 | 5265 | -7.28 | 5.59 | -18.38 | 3.81 | 0.19566 | 0.474626 |
| ANPEP | 290 | -11.64 | 8.90 | -29.39 | 6.11 | 0.19504 | 0.474626 |
| GOLPH3 | 64083 | 10.30 | 7.82 | -5.82 | 26.41 | 0.200131 | 0.480665 |
| FUCA1 | 2517 | -8.87 | 6.85 | -22.54 | 4.79 | 0.199507 | 0.480665 |
| ARHGDIB | 397 | -8.72 | 6.71 | -22.47 | 5.03 | 0.204491 | 0.489 |
| EXTL2 | 2135 | 12.94 | 10.14 | -7.32 | 33.20 | 0.20654 | 0.491761 |
| GPNMB | 10457 | 17.37 | 13.69 | -10.21 | 44.95 | 0.211094 | 0.492388 |
| C8G | 733 | -15.01 | 11.80 | -38.72 | 8.69 | 0.209081 | 0.492388 |
| F5 | 2153 | -14.23 | 11.36 | -36.76 | 8.30 | 0.21307 | 0.492388 |
|  |  | 13.88 | 10.97 | -7.97 | 35.73 | 0.20973 | 0.492388 |
| CD248 | 57124 | 14.76 | 11.67 | -8.76 | 38.29 | 0.212666 | 0.492388 |
| APOA4 | 337 | 1.18 | 0.94 | -0.68 | 3.04 | 0.210776 | 0.492388 |
| APOA4 | 337 | 4.28 | 3.40 | -2.47 | 11.02 | 0.2113 | 0.492388 |
| SILV | 6490 | 24.18 | 18.93 | -15.08 | 63.44 | 0.214743 | 0.494178 |
| A2M | 2 | -9.38 | 7.57 | -24.38 | 5.63 | 0.218104 | 0.499822 |
| ABI3BP | 25890 | 16.01 | 13.09 | -9.95 | 41.96 | 0.224048 | 0.508204 |
| IL1RAP | 3556 | 14.05 | 11.53 | -8.82 | 36.93 | 0.225819 | 0.508204 |
| DBH | 1621 | 5.79 | 4.72 | -3.58 | 15.16 | 0.222979 | 0.508204 |
| ROBO1 | 6091 | 17.79 | 14.46 | -11.51 | 47.10 | 0.226382 | 0.508204 |
| FGL2 | 10875 | 14.90 | 11.83 | -10.05 | 39.85 | 0.224594 | 0.508204 |
| CETP | 1071 | 7.29 | 6.02 | -4.70 | 19.27 | 0.229861 | 0.513916 |
| IGFBP1 | 3484 | -6.40 | 5.28 | -16.98 | 4.18 | 0.23106 | 0.514505 |
| CSPG4 | 1464 | 12.76 | 10.67 | -8.56 | 34.08 | 0.236189 | 0.521702 |
| ACSM3 | 6296 | 15.93 | 13.27 | -10.78 | 42.65 | 0.236074 | 0.521702 |
| PROZ | 8858 | -6.83 | 5.86 | -18.45 | 4.79 | 0.246641 | 0.54261 |
| HLA-C | 3107 | 5.24 | 4.55 | -3.78 | 14.27 | 0.251685 | 0.5515 |
| C2 | 717 | -6.12 | 5.33 | -16.70 | 4.45 | 0.253468 | 0.551766 |
| TFRC | 7037 | -8.70 | 7.57 | -23.74 | 6.34 | 0.253813 | 0.551766 |
| LILRB5 | 10990 | 13.44 | 11.67 | -10.15 | 37.03 | 0.256288 | 0.554953 |
| PGD | 5226 | -9.58 | 8.44 | -26.55 | 7.38 | 0.261844 | 0.564762 |
| C1RL | 51279 | 12.05 | 10.71 | -9.20 | 33.31 | 0.26325 | 0.565576 |
| IGFALS | 3483 | 6.23 | 5.56 | -4.80 | 17.26 | 0.265166 | 0.567476 |
| GGH | 8836 | -12.45 | 11.21 | -34.70 | 9.79 | 0.26938 | 0.569841 |
| TIE1 | 7075 | -11.91 | 10.68 | -33.19 | 9.38 | 0.268421 | 0.569841 |
| CRELD1 | 78987 | 22.03 | 19.46 | -18.06 | 62.11 | 0.268515 | 0.569841 |
| VWF | 7450 | -14.11 | 12.78 | -39.47 | 11.25 | 0.272224 | 0.571434 |
| ITIH4 | 3700 | -12.10 | 10.96 | -33.84 | 9.65 | 0.272349 | 0.571434 |
| LCAT | 3931 | 12.90 | 11.71 | -10.33 | 36.14 | 0.273249 | 0.571434 |
| AGT | 183 | -9.66 | 8.82 | -27.16 | 7.84 | 0.276007 | 0.572845 |
| SERPINA7 | 6906 | -11.44 | 10.43 | -32.13 | 9.24 | 0.275163 | 0.572845 |
| FETUB | 26998 | 9.52 | 8.78 | -7.89 | 26.94 | 0.280542 | 0.578517 |
| ALK | 238 | -12.64 | 11.34 | -36.57 | 11.30 | 0.280844 | 0.578517 |
| C1QB | 713 | 17.35 | 15.95 | -15.32 | 50.02 | 0.28603 | 0.587002 |
|  |  | -8.31 | 7.82 | -23.85 | 7.23 | 0.290998 | 0.594692 |
| UMOD | 7369 | -10.39 | 9.80 | -29.85 | 9.07 | 0.29194 | 0.594692 |
| MMP2 | 4313 | 13.42 | 12.72 | -11.80 | 38.65 | 0.293733 | 0.596137 |
| F11 | 2160 | -13.16 | 12.53 | -38.04 | 11.71 | 0.296071 | 0.596481 |
| FBLN1 | 2192 | -9.76 | 9.28 | -28.18 | 8.65 | 0.295205 | 0.596481 |
| MST1 | 4485 | -8.28 | 8.02 | -24.19 | 7.63 | 0.304353 | 0.608705 |
| CFP | 5199 | -8.23 | 7.96 | -24.05 | 7.59 | 0.304156 | 0.608705 |
| B3GNT1 | 11041 | -10.36 | 10.20 | -30.59 | 9.87 | 0.31222 | 0.622177 |
| ICAM1 | 3383 | -13.49 | 13.55 | -40.37 | 13.39 | 0.321763 | 0.623418 |
| CAST | 831 | -6.66 | 6.56 | -20.25 | 6.94 | 0.320859 | 0.623418 |
|  |  | -11.21 | 11.19 | -33.41 | 10.99 | 0.318975 | 0.623418 |
| PCOLCE | 5118 | 13.33 | 13.44 | -13.34 | 40.00 | 0.323884 | 0.623418 |
| EPHA1 | 2041 | 6.40 | 6.36 | -6.24 | 19.04 | 0.317301 | 0.623418 |
| KIT | 3815 | 14.96 | 15.07 | -15.42 | 45.35 | 0.326162 | 0.623418 |
| TGFBI | 7045 | 14.84 | 15.00 | -14.92 | 44.60 | 0.325016 | 0.623418 |
| CECR1 | 51816 | 8.09 | 8.13 | -8.03 | 24.21 | 0.321616 | 0.623418 |
| IGHG3 | 3502 | -5.89 | 5.97 | -17.74 | 5.96 | 0.326444 | 0.623418 |
| LAMA2 | 3908 | -8.57 | 8.62 | -25.67 | 8.52 | 0.322081 | 0.623418 |
| IGF2 | 3481 | 6.43 | 6.40 | -6.26 | 19.13 | 0.317009 | 0.623418 |
| CRHBP | 1393 | 11.88 | 11.81 | -11.80 | 35.55 | 0.319026 | 0.623418 |
| MASP1 | 5648 | -12.25 | 12.46 | -36.96 | 12.47 | 0.327909 | 0.624049 |
| NAGLU | 4669 | -6.59 | 6.74 | -19.95 | 6.78 | 0.33056 | 0.626924 |
| HPX | 3263 | 6.58 | 6.82 | -6.95 | 20.11 | 0.336787 | 0.636538 |
| SERPINF1 | 5176 | -6.70 | 6.98 | -20.55 | 7.14 | 0.339097 | 0.63871 |
| RNASE4 | 6038 | 23.90 | 24.56 | -27.69 | 75.49 | 0.343342 | 0.644499 |
| TF | 7018 | -5.48 | 5.85 | -17.09 | 6.14 | 0.351638 | 0.655597 |
| IGF2R | 3482 | -8.05 | 8.59 | -25.09 | 9.00 | 0.351337 | 0.655597 |
| FSTL1 | 11167 | 11.07 | 11.93 | -12.60 | 34.74 | 0.355712 | 0.660951 |
| RARRES2 | 5919 | -8.65 | 9.61 | -27.84 | 10.54 | 0.371314 | 0.679585 |
| CNDP1 | 84735 | -4.53 | 5.04 | -14.53 | 5.47 | 0.370515 | 0.679585 |
| SERPINA7 | 6906 | -6.48 | 7.21 | -20.78 | 7.83 | 0.371459 | 0.679585 |
| EFEMP2 | 30008 | -17.13 | 18.65 | -55.80 | 21.54 | 0.368261 | 0.679585 |
| GALNT2 | 2590 | -14.67 | 16.10 | -48.06 | 18.71 | 0.371919 | 0.679585 |
|  |  | -7.69 | 8.79 | -25.12 | 9.74 | 0.383617 | 0.689508 |
| CAST | 831 | -5.05 | 5.70 | -16.48 | 6.37 | 0.37916 | 0.689508 |
| TCN1 | 6947 | -8.56 | 9.74 | -27.93 | 10.81 | 0.381844 | 0.689508 |
| PON3 | 5446 | 12.20 | 13.84 | -15.63 | 40.03 | 0.382507 | 0.689508 |
| PTPRU | 10076 | 3.58 | 4.02 | -4.63 | 11.79 | 0.380082 | 0.689508 |
| ICOSLG | 23308 | 11.94 | 13.86 | -15.55 | 39.44 | 0.390898 | 0.700204 |
| GPLD1 | 2822 | 9.67 | 11.25 | -12.65 | 31.99 | 0.392114 | 0.700204 |
| CLSTN1 | 22883 | 10.76 | 12.67 | -14.39 | 35.90 | 0.398091 | 0.706291 |
| CSK | 1445 | -15.78 | 18.24 | -53.83 | 22.28 | 0.397365 | 0.706291 |
| BTD | 686 | -7.24 | 8.61 | -24.33 | 9.85 | 0.402521 | 0.711853 |
| VWF | 7450 | -8.25 | 9.94 | -27.96 | 11.47 | 0.408735 | 0.720527 |
| TKT | 7086 | 9.36 | 11.33 | -13.29 | 32.01 | 0.411978 | 0.721961 |
| SIRPA | 140885 | -7.65 | 9.25 | -26.21 | 10.91 | 0.412174 | 0.721961 |
| FAM20C | 56975 | 8.00 | 9.76 | -11.35 | 27.36 | 0.413856 | 0.722605 |
| IGH@ | 3492 | 5.07 | 6.22 | -7.26 | 17.41 | 0.416404 | 0.724755 |
| VASN | 114990 | 10.44 | 12.91 | -15.16 | 36.04 | 0.420536 | 0.729637 |
| LMAN2 | 10960 | -12.89 | 16.98 | -48.56 | 22.78 | 0.457604 | 0.735955 |
| CLU | 1191 | -7.03 | 9.39 | -25.66 | 11.60 | 0.455969 | 0.735955 |
| ANPEP | 290 | 11.41 | 15.03 | -18.41 | 41.24 | 0.449606 | 0.735955 |
| ITIH1 | 3697 | 6.55 | 8.42 | -10.16 | 23.27 | 0.438427 | 0.735955 |
| EFEMP1 | 2202 | -5.98 | 7.78 | -21.42 | 9.46 | 0.443928 | 0.735955 |
| APLP1 | 333 | 7.60 | 9.71 | -11.68 | 26.89 | 0.435671 | 0.735955 |
| AHSG | 197 | -8.74 | 11.48 | -31.51 | 14.03 | 0.448153 | 0.735955 |
| ACAN | 176 | 11.88 | 15.86 | -19.58 | 43.34 | 0.455383 | 0.735955 |
| SLC38A10 | 124565 | 7.82 | 9.95 | -11.92 | 27.55 | 0.433785 | 0.735955 |
|  |  | 4.82 | 6.37 | -7.82 | 17.47 | 0.450848 | 0.735955 |
| ACTB | 60 | -5.82 | 7.27 | -20.25 | 8.62 | 0.425725 | 0.735955 |
| KLKB1 | 3818 | -4.82 | 6.26 | -17.24 | 7.60 | 0.443153 | 0.735955 |
| PRAP1 | 118471 | -18.02 | 23.29 | -65.89 | 29.85 | 0.44609 | 0.735955 |
| ALDOB | 229 | -3.75 | 4.77 | -13.24 | 5.73 | 0.43385 | 0.735955 |
| ADIPOQ | 9370 | 4.87 | 6.27 | -7.59 | 17.32 | 0.439861 | 0.735955 |
| SERPINE1 | 5054 | -7.63 | 10.03 | -28.37 | 13.11 | 0.454482 | 0.735955 |
| PNLIP | 5406 | 7.01 | 8.83 | -10.84 | 24.86 | 0.431896 | 0.735955 |
| PLD4 | 122618 | -9.87 | 13.18 | -36.40 | 16.66 | 0.45763 | 0.735955 |
| PSMC3IP | 29893 | -3.67 | 4.56 | -13.16 | 5.81 | 0.429692 | 0.735955 |
| ATF6 | 22926 | 19.46 | 24.26 | -30.23 | 69.14 | 0.429278 | 0.735955 |
| IGHD | 3495 | 3.30 | 4.42 | -5.47 | 12.08 | 0.456391 | 0.735955 |
| AMH | 268 | 6.53 | 8.52 | -10.71 | 23.77 | 0.447913 | 0.735955 |
| C1RL | 51279 | 1.91 | 2.54 | -3.14 | 6.95 | 0.455081 | 0.735955 |
| PSMA1 | 5682 | -5.06 | 6.61 | -18.25 | 8.13 | 0.446678 | 0.735955 |
| PTK2B | 2185 | 3.61 | 4.58 | -5.72 | 12.94 | 0.436663 | 0.735955 |
| CPB2 | 1361 | -7.58 | 10.27 | -27.95 | 12.79 | 0.462304 | 0.739149 |
| B2M | 567 | 8.86 | 11.89 | -15.39 | 33.11 | 0.461739 | 0.739149 |
| CPN1 | 1369 | -7.12 | 9.69 | -26.35 | 12.10 | 0.463999 | 0.739708 |
| FMOD | 2331 | 6.39 | 8.71 | -11.13 | 23.91 | 0.467042 | 0.740269 |
| FGL1 | 2267 | -4.94 | 6.68 | -18.67 | 8.78 | 0.465821 | 0.740269 |
| TIMP1 | 7076 | -9.09 | 12.58 | -34.18 | 16.00 | 0.472274 | 0.746409 |
| LRP1 | 4035 | -8.99 | 12.52 | -33.90 | 15.93 | 0.475011 | 0.746446 |
| HYOU1 | 10525 | 9.60 | 13.38 | -16.95 | 36.15 | 0.474987 | 0.746446 |
| MBL2 | 4153 | 4.62 | 6.50 | -8.28 | 17.51 | 0.479064 | 0.750671 |
| FUCA2 | 2519 | -6.13 | 8.66 | -23.49 | 11.23 | 0.482054 | 0.75321 |
| GRN | 2896 | 8.47 | 12.04 | -15.61 | 32.56 | 0.484352 | 0.754657 |
| C4A | 720 | -6.17 | 8.85 | -23.90 | 11.55 | 0.488231 | 0.758551 |
| C8A | 731 | 4.43 | 6.46 | -8.40 | 17.26 | 0.494713 | 0.761506 |
| IGFBP2 | 3485 | 3.74 | 5.44 | -7.06 | 14.54 | 0.493798 | 0.761506 |
| IGF1 | 3479 | -3.95 | 5.74 | -15.34 | 7.44 | 0.493142 | 0.761506 |
| SERPINF1 | 5176 | -2.22 | 3.24 | -8.66 | 4.22 | 0.495671 | 0.761506 |
| KRT10 | 3858 | 4.59 | 6.72 | -8.90 | 18.09 | 0.497509 | 0.762201 |
|  |  | -5.80 | 8.65 | -22.95 | 11.35 | 0.503646 | 0.76946 |
| ENPP2 | 5168 | 6.93 | 10.38 | -13.67 | 27.52 | 0.506095 | 0.771059 |
| SPON1 | 10418 | -12.28 | 18.33 | -50.11 | 25.54 | 0.509139 | 0.771423 |
| RBP4 | 5950 | 4.30 | 6.47 | -8.54 | 17.13 | 0.50807 | 0.771423 |
| SDC1 | 6382 | -5.96 | 9.16 | -24.18 | 12.26 | 0.517397 | 0.781782 |
| NRCAM | 4897 | 6.49 | 10.03 | -13.42 | 26.40 | 0.519239 | 0.782415 |
| APOM | 55937 | 2.85 | 4.43 | -5.94 | 11.64 | 0.521708 | 0.783987 |
| SPP2 | 6694 | 7.30 | 11.42 | -15.45 | 30.04 | 0.524764 | 0.786431 |
| CHL1 | 10752 | 10.60 | 16.71 | -22.55 | 43.76 | 0.527248 | 0.787785 |
| SEPP1 | 6414 | 9.25 | 14.63 | -19.77 | 38.27 | 0.528532 | 0.787785 |
| FLT4 | 2324 | 8.35 | 13.34 | -18.23 | 34.93 | 0.533263 | 0.788426 |
| APOA2 | 336 | -6.67 | 10.63 | -27.75 | 14.42 | 0.531875 | 0.788426 |
|  |  | -7.38 | 11.70 | -31.01 | 16.26 | 0.531882 | 0.788426 |
| FGFR1 | 2260 | 7.92 | 12.93 | -17.90 | 33.73 | 0.542572 | 0.800039 |
| CD163 | 9332 | 7.96 | 13.21 | -18.25 | 34.18 | 0.547973 | 0.805843 |
| COL11A2 | 1302 | 4.59 | 7.70 | -10.68 | 19.86 | 0.552198 | 0.808315 |
| CD109 | 135228 | -6.44 | 10.80 | -27.88 | 15.00 | 0.552594 | 0.808315 |
| PTPRS | 5802 | 6.73 | 11.38 | -15.90 | 29.36 | 0.555817 | 0.809471 |
| KNG1 | 3827 | 8.38 | 14.30 | -19.99 | 36.75 | 0.559094 | 0.809471 |
| ICAM2 | 3384 | 9.52 | 16.24 | -22.71 | 41.75 | 0.559271 | 0.809471 |
| SERPINA4 | 5267 | 3.34 | 5.69 | -7.96 | 14.63 | 0.559094 | 0.809471 |
| ANG | 283 | 7.95 | 13.65 | -19.28 | 35.18 | 0.5622 | 0.811575 |
| PON1 | 5444 | -3.28 | 5.68 | -14.54 | 7.98 | 0.564525 | 0.812797 |
| F10 | 2159 | -2.69 | 4.68 | -11.97 | 6.58 | 0.566218 | 0.813107 |
| BST1 | 683 | 4.30 | 7.57 | -10.72 | 19.33 | 0.570985 | 0.815693 |
| OSCAR | 126014 | 4.80 | 8.40 | -11.92 | 21.51 | 0.569683 | 0.815693 |
| SCARA3 | 51435 | -14.74 | 25.85 | -68.49 | 39.01 | 0.574548 | 0.818657 |
| C6 | 729 | -4.00 | 7.24 | -18.36 | 10.37 | 0.582145 | 0.825344 |
| IGHA1 | 3493 | -2.64 | 4.79 | -12.14 | 6.86 | 0.582243 | 0.825344 |
| APOC1 | 341 | 4.95 | 9.05 | -13.01 | 22.91 | 0.585765 | 0.828202 |
| COL3A1 | 1281 | -5.64 | 10.78 | -27.04 | 15.77 | 0.602219 | 0.830126 |
| CPN2 | 1370 | -6.37 | 12.15 | -30.47 | 17.74 | 0.60155 | 0.830126 |
|  | 5648 | -7.62 | 14.04 | -35.47 | 20.24 | 0.588718 | 0.830126 |
| PRG2 | 5553 | -4.58 | 8.59 | -21.63 | 12.46 | 0.594898 | 0.830126 |
| DSG2 | 1829 | -6.27 | 11.94 | -30.02 | 17.48 | 0.60087 | 0.830126 |
| HABP2 | 3026 | -5.21 | 9.82 | -24.69 | 14.27 | 0.596912 | 0.830126 |
| LAMP2 | 3920 | -8.46 | 15.71 | -39.62 | 22.71 | 0.59148 | 0.830126 |
| HAPLN1 | 1404 | 7.99 | 15.05 | -22.57 | 38.55 | 0.598833 | 0.830126 |
| C4A | 720 | 5.14 | 9.64 | -14.01 | 24.29 | 0.595262 | 0.830126 |
| EXT2 | 2132 | -8.32 | 15.44 | -40.89 | 24.26 | 0.596974 | 0.830126 |
| MRC1 | 4360 | 5.99 | 11.81 | -17.53 | 29.51 | 0.613476 | 0.838686 |
| ADAMTS13 | 11093 | 5.95 | 11.78 | -17.42 | 29.32 | 0.614528 | 0.838686 |
| C8B | 732 | 5.21 | 10.30 | -15.22 | 25.63 | 0.614059 | 0.838686 |
| CD44 | 960 | 6.38 | 12.58 | -18.57 | 31.33 | 0.613073 | 0.838686 |
| APOA1 | 335 | 2.46 | 4.99 | -7.45 | 12.37 | 0.62366 | 0.842783 |
|  |  | 4.45 | 8.99 | -13.49 | 22.39 | 0.62221 | 0.842783 |
| A2M | 2 | -0.78 | 1.58 | -3.91 | 2.35 | 0.620945 | 0.842783 |
| BASP1 | 10409 | 4.40 | 8.89 | -13.37 | 22.18 | 0.622184 | 0.842783 |
| SERPINA5 | 5104 | -7.14 | 14.64 | -36.51 | 22.23 | 0.627878 | 0.846405 |
| LTBP4 | 8425 | -7.49 | 15.50 | -39.73 | 24.76 | 0.634202 | 0.852838 |
| C1R | 715 | 3.92 | 8.32 | -12.58 | 20.42 | 0.638592 | 0.856647 |
| NME3 | 4832 | 8.93 | 19.25 | -31.10 | 48.96 | 0.647466 | 0.866438 |
| LDHB | 3945 | 2.58 | 5.85 | -9.03 | 14.19 | 0.660736 | 0.878845 |
| IGHG4 | 3503 | 1.49 | 3.38 | -5.22 | 8.19 | 0.661207 | 0.878845 |
| COL5A1 | 1289 | -5.78 | 13.09 | -32.36 | 20.80 | 0.661531 | 0.878845 |
| RTN4RL2 | 349667 | -3.77 | 8.62 | -21.06 | 13.51 | 0.663229 | 0.878978 |
| MINPP1 | 9562 | 6.41 | 15.19 | -23.72 | 36.54 | 0.67395 | 0.891039 |
| MASP2 | 10747 | -2.80 | 6.81 | -16.32 | 10.71 | 0.681432 | 0.893116 |
| ITIH3 | 3699 | -4.41 | 10.65 | -25.55 | 16.73 | 0.679721 | 0.893116 |
| C1S | 716 | -3.52 | 8.80 | -20.98 | 13.93 | 0.689592 | 0.893116 |
| PGLYRP2 | 114770 | -2.61 | 6.54 | -15.58 | 10.36 | 0.690417 | 0.893116 |
| LTBP1 | 4052 | -6.30 | 15.62 | -37.83 | 25.23 | 0.688814 | 0.893116 |
| CDH1 | 999 | 4.90 | 12.36 | -19.66 | 29.46 | 0.692668 | 0.893116 |
| LYVE1 | 10894 | 4.79 | 12.06 | -19.16 | 28.74 | 0.692206 | 0.893116 |
| LSAMP | 4045 | 5.44 | 13.17 | -20.71 | 31.60 | 0.680436 | 0.893116 |
| FTL | 2512 | -3.15 | 7.77 | -18.59 | 12.29 | 0.686128 | 0.893116 |
| CLPX | 10845 | 5.14 | 12.94 | -20.93 | 31.21 | 0.693383 | 0.893116 |
| FCGR3B | 2215 | -2.02 | 5.06 | -12.10 | 8.06 | 0.690572 | 0.893116 |
| CORO1A | 11151 | -3.20 | 8.37 | -19.97 | 13.56 | 0.703326 | 0.895705 |
| LGALS3BP | 3959 | -2.41 | 6.34 | -14.99 | 10.18 | 0.705164 | 0.895705 |
| SIGLEC5 | 8778 | 3.21 | 8.41 | -13.58 | 19.99 | 0.704197 | 0.895705 |
| PTPRD | 5789 | 4.20 | 10.87 | -17.65 | 26.06 | 0.700706 | 0.895705 |
| FCN2 | 2220 | -4.78 | 12.53 | -30.01 | 20.46 | 0.704778 | 0.895705 |
| SERPINA11 | 256394 | -2.83 | 7.31 | -17.34 | 11.69 | 0.699935 | 0.895705 |
| LOC100289383 | 1E+08 | -0.94 | 2.51 | -5.95 | 4.06 | 0.707968 | 0.897194 |
| FCGBP | 8857 | 6.36 | 17.14 | -27.66 | 40.37 | 0.711509 | 0.898645 |
| PLXDC2 | 84898 | -3.59 | 9.72 | -22.90 | 15.71 | 0.71238 | 0.898645 |
| PODXL | 5420 | -3.28 | 8.93 | -21.19 | 14.64 | 0.715095 | 0.900005 |
| IGFBP4 | 3487 | -4.08 | 11.25 | -26.43 | 18.27 | 0.717462 | 0.900923 |
|  |  | -2.39 | 6.62 | -15.52 | 10.74 | 0.719167 | 0.901007 |
| KRT1 | 3848 | -1.40 | 4.04 | -9.42 | 6.61 | 0.729167 | 0.911459 |
| HSP90B1 | 7184 | 3.63 | 11.28 | -18.78 | 26.04 | 0.748519 | 0.921306 |
| ALB | 213 | 2.08 | 6.31 | -10.44 | 14.61 | 0.74218 | 0.921306 |
| APOB | 338 | -4.26 | 12.77 | -29.59 | 21.07 | 0.739496 | 0.921306 |
| SPARC | 6678 | -2.07 | 6.26 | -14.53 | 10.39 | 0.741564 | 0.921306 |
| LCN2 | 3934 | -2.93 | 9.10 | -21.10 | 15.25 | 0.74877 | 0.921306 |
| MOS | 4342 | 2.10 | 6.50 | -10.82 | 15.01 | 0.747791 | 0.921306 |
| GOLM1 | 51280 | 4.85 | 14.88 | -25.08 | 34.77 | 0.746134 | 0.921306 |
| CTSZ | 1522 | 3.98 | 12.50 | -21.09 | 29.05 | 0.751749 | 0.921502 |
| EXT1 | 2131 | -2.38 | 7.51 | -17.48 | 12.72 | 0.752281 | 0.921502 |
| B3GAT3 | 26229 | 2.47 | 7.97 | -14.11 | 19.05 | 0.75992 | 0.928791 |
| AXL | 558 | 2.05 | 6.88 | -11.72 | 15.82 | 0.766454 | 0.92908 |
| ICAM3 | 3385 | 3.80 | 13.15 | -22.31 | 29.91 | 0.77332 | 0.92908 |
|  |  | 1.69 | 5.81 | -9.83 | 13.22 | 0.7711 | 0.92908 |
|  |  | -2.56 | 8.75 | -21.01 | 15.90 | 0.773524 | 0.92908 |
| TMEM132C | 92293 | 3.36 | 11.60 | -20.02 | 26.73 | 0.77367 | 0.92908 |
| FAM3C | 10447 | -3.46 | 11.68 | -26.79 | 19.88 | 0.76829 | 0.92908 |
| KRT9 | 3857 | 4.07 | 14.00 | -24.95 | 33.10 | 0.773667 | 0.92908 |
| PLXNA1 | 5361 | 4.63 | 15.75 | -27.38 | 36.63 | 0.770714 | 0.92908 |
| H6PD | 9563 | -2.19 | 7.73 | -17.54 | 13.16 | 0.777111 | 0.929234 |
| SERPINC1 | 462 | 1.45 | 5.13 | -8.74 | 11.63 | 0.778867 | 0.929234 |
| PPIB | 5479 | 2.36 | 8.32 | -14.51 | 19.22 | 0.778718 | 0.929234 |
| A1BG | 1 | -1.29 | 4.69 | -10.59 | 8.01 | 0.783811 | 0.930702 |
| C1QC | 714 | 2.62 | 9.73 | -16.75 | 21.98 | 0.788514 | 0.930702 |
| APOC3 | 345 | 1.54 | 5.72 | -9.81 | 12.89 | 0.788559 | 0.930702 |
| C7 | 730 | 1.82 | 6.68 | -11.42 | 15.07 | 0.785194 | 0.930702 |
| RNASE2 | 6036 | -5.24 | 19.19 | -43.84 | 33.37 | 0.786182 | 0.930702 |
| CST3 | 1471 | 2.56 | 10.03 | -17.34 | 22.46 | 0.799032 | 0.931341 |
| CD14 | 929 | -2.57 | 9.96 | -22.32 | 17.19 | 0.797126 | 0.931341 |
| PTGDS | 5730 | 3.06 | 12.01 | -20.76 | 26.88 | 0.799259 | 0.931341 |
| F10 | 2159 | 3.36 | 12.82 | -22.07 | 28.79 | 0.793792 | 0.931341 |
| FCGR2A | 2212 | 1.84 | 7.03 | -12.11 | 15.78 | 0.794261 | 0.931341 |
| SOD2 | 6648 | -2.93 | 11.15 | -25.26 | 19.41 | 0.79385 | 0.931341 |
| PLG | 5340 | -1.74 | 7.34 | -16.29 | 12.81 | 0.812982 | 0.931352 |
| BCHE | 590 | -3.25 | 12.90 | -28.83 | 22.34 | 0.801816 | 0.931352 |
|  |  | 2.09 | 8.87 | -15.52 | 19.69 | 0.814623 | 0.931352 |
| FCN3 | 8547 | 2.55 | 10.99 | -19.24 | 24.35 | 0.816708 | 0.931352 |
| PSMB2 | 5690 | -2.39 | 10.26 | -23.25 | 18.48 | 0.817555 | 0.931352 |
| APOD | 347 | -2.60 | 11.37 | -25.16 | 19.96 | 0.819589 | 0.931352 |
|  |  | 1.38 | 5.63 | -9.80 | 12.55 | 0.807267 | 0.931352 |
|  |  | -1.74 | 7.30 | -16.23 | 12.74 | 0.811942 | 0.931352 |
| F5 | 2153 | -2.40 | 9.96 | -22.21 | 17.41 | 0.810059 | 0.931352 |
| LAMB1 | 3912 | 2.72 | 11.75 | -20.60 | 26.05 | 0.817226 | 0.931352 |
| LAMP1 | 3916 | -3.26 | 13.36 | -29.94 | 23.41 | 0.807835 | 0.931352 |
| ESAM | 90952 | 2.46 | 10.64 | -19.00 | 23.93 | 0.818013 | 0.931352 |
| AMY2B | 280 | 2.26 | 10.10 | -17.78 | 22.30 | 0.823464 | 0.931904 |
| NEGR1 | 257194 | -2.08 | 9.28 | -20.51 | 16.34 | 0.822986 | 0.931904 |
| DKK3 | 27122 | -2.43 | 11.01 | -24.89 | 20.04 | 0.827026 | 0.934013 |
|  | 2192 | -1.80 | 8.44 | -18.54 | 14.94 | 0.831526 | 0.936797 |
| OPCML | 4978 | 3.38 | 15.83 | -29.53 | 36.29 | 0.832898 | 0.936797 |
| CFH | 3075 | -2.12 | 10.36 | -22.79 | 18.55 | 0.838477 | 0.939735 |
| LYZ | 4069 | 2.49 | 12.18 | -22.06 | 27.05 | 0.838927 | 0.939735 |
| CADM1 | 23705 | 4.45 | 22.18 | -40.03 | 48.93 | 0.84175 | 0.940981 |
| C1QTNF5 | 114902 | 2.89 | 14.53 | -27.03 | 32.82 | 0.84376 | 0.941315 |
| APOE | 348 | -1.40 | 7.24 | -15.77 | 12.97 | 0.84733 | 0.943383 |
| ENPEP | 2028 | -1.72 | 9.03 | -19.68 | 16.24 | 0.84955 | 0.943945 |
| F7 | 2155 | -1.81 | 9.79 | -21.24 | 17.61 | 0.853522 | 0.946445 |
| IGL@ | 3535 | 1.40 | 7.81 | -14.09 | 16.90 | 0.85787 | 0.947447 |
| PTPRJ | 5795 | 2.44 | 13.58 | -24.51 | 29.38 | 0.857808 | 0.947447 |
| MAN1A1 | 4121 | -1.41 | 8.12 | -17.52 | 14.70 | 0.862502 | 0.950654 |
|  |  | -2.88 | 16.79 | -37.61 | 31.85 | 0.865377 | 0.951491 |
| MMRN1 | 22915 | -3.39 | 20.05 | -44.23 | 37.44 | 0.866721 | 0.951491 |
| SEMA3F | 6405 | 2.91 | 17.67 | -33.74 | 39.56 | 0.870613 | 0.952273 |
| ALDOC | 230 | 3.49 | 21.59 | -41.87 | 48.84 | 0.873507 | 0.952273 |
| IGHG2 | 3501 | 0.82 | 5.16 | -9.42 | 11.06 | 0.87436 | 0.952273 |
| CA11 | 770 | -2.15 | 13.19 | -28.78 | 24.47 | 0.871052 | 0.952273 |
| PRCP | 5547 | 1.61 | 10.88 | -20.19 | 23.40 | 0.882866 | 0.952657 |
| F2 | 2147 | -0.48 | 3.33 | -7.09 | 6.13 | 0.885105 | 0.952657 |
|  |  | -1.39 | 9.00 | -19.38 | 16.60 | 0.878006 | 0.952657 |
| ANGPTL3 | 27329 | 1.57 | 10.76 | -19.84 | 22.97 | 0.884571 | 0.952657 |
| B3GNT2 | 10678 | 0.73 | 4.91 | -9.05 | 10.51 | 0.882077 | 0.952657 |
| FLNB | 2317 | 1.57 | 10.61 | -20.33 | 23.47 | 0.883587 | 0.952657 |
| CEACAM1 | 634 | 1.25 | 9.34 | -17.39 | 19.88 | 0.894131 | 0.956314 |
| FBLN1 | 2192 | -1.05 | 7.70 | -16.33 | 14.24 | 0.892155 | 0.956314 |
| TNC | 3371 | -2.04 | 14.93 | -31.67 | 27.58 | 0.891363 | 0.956314 |
| CTSB | 1508 | -1.99 | 15.05 | -32.38 | 28.40 | 0.895458 | 0.956314 |
|  |  | 1.05 | 8.14 | -15.17 | 17.28 | 0.897604 | 0.956748 |
| CCDC80 | 151887 | -1.39 | 11.40 | -24.40 | 21.62 | 0.903423 | 0.959233 |
| CNTFR | 1271 | -2.54 | 20.38 | -44.69 | 39.62 | 0.901964 | 0.959233 |
|  |  | -0.98 | 8.36 | -17.57 | 15.61 | 0.906765 | 0.960926 |
| ALCAM | 214 | 1.73 | 15.58 | -29.17 | 32.63 | 0.911672 | 0.964268 |
| F12 | 2161 | -0.77 | 7.25 | -15.15 | 13.61 | 0.915366 | 0.964588 |
| MEGF8 | 1954 | 1.09 | 10.82 | -20.39 | 22.56 | 0.920032 | 0.964588 |
| SEMA4B | 10509 | -1.90 | 18.95 | -40.17 | 36.37 | 0.920743 | 0.964588 |
|  |  | -0.60 | 5.91 | -12.35 | 11.15 | 0.919024 | 0.964588 |
| RNASE1 | 6035 | 1.06 | 10.12 | -19.27 | 21.38 | 0.917325 | 0.964588 |
| AZGP1 | 563 | 0.90 | 9.96 | -18.86 | 20.66 | 0.928065 | 0.968568 |
| IGL@ | 3535 | -0.63 | 6.87 | -14.25 | 13.00 | 0.927548 | 0.968568 |
|  | 51279 | -0.85 | 11.87 | -24.39 | 22.70 | 0.943256 | 0.969702 |
| LTA4H | 4048 | 0.65 | 9.03 | -17.42 | 18.71 | 0.942915 | 0.969702 |
| HRG | 3273 | -1.12 | 13.37 | -27.64 | 25.39 | 0.933186 | 0.969702 |
| IDH3A | 3419 | -0.47 | 6.52 | -13.43 | 12.50 | 0.943036 | 0.969702 |
| IL1R2 | 7850 | -0.75 | 9.83 | -20.51 | 19.01 | 0.93939 | 0.969702 |
| APOC4 | 346 | 0.57 | 7.34 | -14.10 | 15.25 | 0.937914 | 0.969702 |
| F2 | 2147 | 0.28 | 3.46 | -6.58 | 7.13 | 0.936296 | 0.969702 |
| SEC31B | 25956 | -1.46 | 17.33 | -37.40 | 34.47 | 0.933469 | 0.969702 |
| MSN | 4478 | -0.44 | 6.82 | -13.98 | 13.11 | 0.949232 | 0.974025 |
| OLFM1 | 10439 | -0.67 | 15.37 | -31.16 | 29.82 | 0.965469 | 0.988839 |
| APOC2 | 344 | 0.29 | 7.54 | -14.66 | 15.24 | 0.968965 | 0.988839 |
| FBN1 | 2200 | 0.39 | 10.02 | -19.51 | 20.29 | 0.969062 | 0.988839 |
| IGFBP3 | 3486 | 0.08 | 8.08 | -15.94 | 16.11 | 0.991866 | 0.991866 |
| FN1 | 2335 | 0.19 | 9.06 | -17.81 | 18.20 | 0.983204 | 0.991866 |
| ATRN | 8455 | 0.23 | 18.13 | -35.73 | 36.19 | 0.99004 | 0.991866 |
| ALDOA | 226 | 0.20 | 7.57 | -14.84 | 15.24 | 0.978823 | 0.991866 |
| TCN2 | 6948 | -0.12 | 10.58 | -21.18 | 20.95 | 0.99104 | 0.991866 |
| CFHR1 | 3078 | -0.09 | 8.34 | -17.01 | 16.83 | 0.991114 | 0.991866 |
| IGDCC4 | 57722 | -0.23 | 9.49 | -19.10 | 18.63 | 0.980439 | 0.991866 |
| TRA@ | 6955 | 0.24 | 8.57 | -17.57 | 18.06 | 0.9776 | 0.991866 |
| SERPINF1 | 5176 | -0.08 | 6.87 | -13.71 | 13.55 | 0.990614 | 0.991866 |
| SOD3 | 6649 | 0.06 | 4.12 | -8.11 | 8.23 | 0.988422 | 0.991866 |
|  |  | -0.15 | 6.74 | -13.56 | 13.26 | 0.98262 | 0.991866 |
| FERMT2 | 10979 | NA | NA | NA | NA | NA | NA |
| SLC33A1 | 9197 | NA | NA | NA | NA | NA | NA |
| NPHS2 | 7827 | NA | NA | NA | NA | NA | NA |
| CNTN3 | 5067 | NA | NA | NA | NA | NA | NA |
| C22orf31 | 25770 | NA | NA | NA | NA | NA | NA |
| POMGNT1 | 55624 | NA | NA | NA | NA | NA | NA |
| GPD1 | 2819 | NA | NA | NA | NA | NA | NA |
| STOML2 | 30968 | NA | NA | NA | NA | NA | NA |
| PCNT | 5116 | NA | NA | NA | NA | NA | NA |
| GUCA2A | 2980 | NA | NA | NA | NA | NA | NA |
| KLHDC7B | 113730 | NA | NA | NA | NA | NA | NA |
| BXDC1 | 84154 | NA | NA | NA | NA | NA | NA |
| UBE2V1 | 7335 | NA | NA | NA | NA | NA | NA |
| TYMP | 1890 | NA | NA | NA | NA | NA | NA |
| NCAM2 | 4685 | NA | NA | NA | NA | NA | NA |
| GPR116 | 221395 | NA | NA | NA | NA | NA | NA |
| PRDX5 | 25824 | NA | NA | NA | NA | NA | NA |
| C9orf141 | 203235 | NA | NA | NA | NA | NA | NA |
| TIMD4 | 91937 | NA | NA | NA | NA | NA | NA |
| MAN2A2 | 4122 | NA | NA | NA | NA | NA | NA |
| SPINK5 | 11005 | NA | NA | NA | NA | NA | NA |
| MARCO | 8685 | NA | NA | NA | NA | NA | NA |
| CD5L | 922 | NA | NA | NA | NA | NA | NA |
| MERTK | 10461 | NA | NA | NA | NA | NA | NA |
| DNHD1 | 144132 | NA | NA | NA | NA | NA | NA |
| C6orf174 | 387104 | NA | NA | NA | NA | NA | NA |
| PPIL2 | 23759 | NA | NA | NA | NA | NA | NA |
| DBI | 1622 | NA | NA | NA | NA | NA | NA |
| DCUN1D1 | 54165 | NA | NA | NA | NA | NA | NA |
| SEPSECS | 51091 | NA | NA | NA | NA | NA | NA |
| LOC100289290 | 1E+08 | NA | NA | NA | NA | NA | NA |
| VCP | 7415 | NA | NA | NA | NA | NA | NA |
| MEGF10 | 84466 | NA | NA | NA | NA | NA | NA |
| PAPLN | 89932 | NA | NA | NA | NA | NA | NA |
| TGFBR3 | 7049 | NA | NA | NA | NA | NA | NA |
| ANXA3 | 306 | NA | NA | NA | NA | NA | NA |
| FCGRT | 2217 | NA | NA | NA | NA | NA | NA |
| ZNF770 | 54989 | NA | NA | NA | NA | NA | NA |
| LAMB2 | 3913 | NA | NA | NA | NA | NA | NA |
| PLXDC1 | 57125 | NA | NA | NA | NA | NA | NA |
| FAM135B | 51059 | NA | NA | NA | NA | NA | NA |
| CENPF | 1063 | NA | NA | NA | NA | NA | NA |
| EYA3 | 2140 | NA | NA | NA | NA | NA | NA |
| TCL6 | 27004 | NA | NA | NA | NA | NA | NA |
| GPR123 | 84435 | NA | NA | NA | NA | NA | NA |
| CCL16 | 6360 | NA | NA | NA | NA | NA | NA |
| TGOLN2 | 10618 | NA | NA | NA | NA | NA | NA |
| SEMA7A | 8482 | NA | NA | NA | NA | NA | NA |
| COLEC11 | 78989 | NA | NA | NA | NA | NA | NA |
| ACE | 1636 | NA | NA | NA | NA | NA | NA |
| RNH1 | 6050 | NA | NA | NA | NA | NA | NA |
| TALDO1 | 6888 | NA | NA | NA | NA | NA | NA |
| SLC25A11 | 8402 | NA | NA | NA | NA | NA | NA |
| LOC100133794 | 1E+08 | NA | NA | NA | NA | NA | NA |
| ARPC3 | 10094 | NA | NA | NA | NA | NA | NA |
| OIT3 | 170392 | NA | NA | NA | NA | NA | NA |
| IRX3 | 79191 | NA | NA | NA | NA | NA | NA |
| PVRL1 | 5818 | NA | NA | NA | NA | NA | NA |
| PSMB1 | 5689 | NA | NA | NA | NA | NA | NA |
| PGAM2 | 5224 | NA | NA | NA | NA | NA | NA |
| RREB1 | 6239 | NA | NA | NA | NA | NA | NA |
| TK1 | 7083 | NA | NA | NA | NA | NA | NA |
| PPEF1 | 5475 | NA | NA | NA | NA | NA | NA |
| ZNF496 | 84838 | NA | NA | NA | NA | NA | NA |
| NAALADL2 | 254827 | NA | NA | NA | NA | NA | NA |
| PLEKHA5 | 54477 | NA | NA | NA | NA | NA | NA |
| PIGR | 5284 | NA | NA | NA | NA | NA | NA |
| PCGF2 | 7703 | NA | NA | NA | NA | NA | NA |
| CDH6 | 1004 | NA | NA | NA | NA | NA | NA |
| MCM10 | 55388 | NA | NA | NA | NA | NA | NA |
| SLC14A2 | 8170 | NA | NA | NA | NA | NA | NA |
| SMCR8 | 140775 | NA | NA | NA | NA | NA | NA |
| NPHS1 | 4868 | NA | NA | NA | NA | NA | NA |
| EPAS1 | 2034 | NA | NA | NA | NA | NA | NA |
| COL15A1 | 1306 | NA | NA | NA | NA | NA | NA |
| COG7 | 91949 | NA | NA | NA | NA | NA | NA |
| PEBP1 | 5037 | NA | NA | NA | NA | NA | NA |
| KCNG4 | 93107 | NA | NA | NA | NA | NA | NA |
| SPTAN1 | 6709 | NA | NA | NA | NA | NA | NA |
| CPD | 1362 | NA | NA | NA | NA | NA | NA |
| GPT | 2875 | NA | NA | NA | NA | NA | NA |
| NFKB2 | 4791 | NA | NA | NA | NA | NA | NA |
| REG1A | 5967 | NA | NA | NA | NA | NA | NA |
| FBXO38 | 81545 | NA | NA | NA | NA | NA | NA |
| SRPRB | 58477 | NA | NA | NA | NA | NA | NA |
| FBLN5 | 10516 | NA | NA | NA | NA | NA | NA |
| NAP1L4 | 4676 | NA | NA | NA | NA | NA | NA |
| NAMPT | 10135 | NA | NA | NA | NA | NA | NA |
| DNAH5 | 1767 | NA | NA | NA | NA | NA | NA |
| CUTA | 51596 | NA | NA | NA | NA | NA | NA |
| KCTD12 | 115207 | NA | NA | NA | NA | NA | NA |
| LSG1 | 55341 | NA | NA | NA | NA | NA | NA |
| CALCR | 799 | NA | NA | NA | NA | NA | NA |
| PSME1 | 5720 | NA | NA | NA | NA | NA | NA |
| TRAF5 | 7188 | NA | NA | NA | NA | NA | NA |
| ADAMDEC1 | 27299 | NA | NA | NA | NA | NA | NA |
| OGN | 4969 | NA | NA | NA | NA | NA | NA |
| GOT1 | 2805 | NA | NA | NA | NA | NA | NA |
| PAM | 5066 | NA | NA | NA | NA | NA | NA |
| OAF | 220323 | NA | NA | NA | NA | NA | NA |
| PLEKHG4B | 153478 | NA | NA | NA | NA | NA | NA |
| LOC100130248 | 1E+08 | NA | NA | NA | NA | NA | NA |
| NDEL1 | 81565 | NA | NA | NA | NA | NA | NA |
| TRIM41 | 90933 | NA | NA | NA | NA | NA | NA |
| GALNS | 2588 | NA | NA | NA | NA | NA | NA |
| PLXND1 | 23129 | NA | NA | NA | NA | NA | NA |
| NUTF2 | 10204 | NA | NA | NA | NA | NA | NA |
| HEXB | 3074 | NA | NA | NA | NA | NA | NA |
| APC | 324 | NA | NA | NA | NA | NA | NA |
| PDCD6 | 10016 | NA | NA | NA | NA | NA | NA |
| CANT1 | 124583 | NA | NA | NA | NA | NA | NA |
| CABC1 | 56997 | NA | NA | NA | NA | NA | NA |
| PDIA6 | 10130 | NA | NA | NA | NA | NA | NA |
| FABP4 | 2167 | NA | NA | NA | NA | NA | NA |
| ITGAM | 3684 | NA | NA | NA | NA | NA | NA |
| MED8 | 112950 | NA | NA | NA | NA | NA | NA |
| APEH | 327 | NA | NA | NA | NA | NA | NA |
| LOC100289629 | 1E+08 | NA | NA | NA | NA | NA | NA |
| FTSJD2 | 23070 | NA | NA | NA | NA | NA | NA |
| DDX53 | 168400 | NA | NA | NA | NA | NA | NA |
| GHR | 2690 | NA | NA | NA | NA | NA | NA |
| NAPRT1 | 93100 | NA | NA | NA | NA | NA | NA |
| LOC344065 | 344065 | NA | NA | NA | NA | NA | NA |
| RNF169 | 254225 | NA | NA | NA | NA | NA | NA |
| PDCD1LG2 | 80380 | NA | NA | NA | NA | NA | NA |
| SPTBN1 | 6711 | NA | NA | NA | NA | NA | NA |
| HPCA | 3208 | NA | NA | NA | NA | NA | NA |
| CEP135 | 9662 | NA | NA | NA | NA | NA | NA |
| PUF60 | 22827 | NA | NA | NA | NA | NA | NA |
| KIF3B | 9371 | NA | NA | NA | NA | NA | NA |
| DCTN1 | 1639 | NA | NA | NA | NA | NA | NA |
| TUBA4A | 7277 | NA | NA | NA | NA | NA | NA |
| COTL1 | 23406 | NA | NA | NA | NA | NA | NA |
| COBRA1 | 25920 | NA | NA | NA | NA | NA | NA |
| SMPDL3A | 10924 | NA | NA | NA | NA | NA | NA |
| B4GALT1 | 2683 | NA | NA | NA | NA | NA | NA |
| RPL21 | 6144 | NA | NA | NA | NA | NA | NA |
| HIC2 | 23119 | NA | NA | NA | NA | NA | NA |
| ANAPC1 | 64682 | NA | NA | NA | NA | NA | NA |
| ME3 | 10873 | NA | NA | NA | NA | NA | NA |
| SQLE | 6713 | NA | NA | NA | NA | NA | NA |
| NAPA | 8775 | NA | NA | NA | NA | NA | NA |
| ATP8B3 | 148229 | NA | NA | NA | NA | NA | NA |
| GALNT7 | 51809 | NA | NA | NA | NA | NA | NA |
| MKI67 | 4288 | NA | NA | NA | NA | NA | NA |
| RPN2 | 6185 | NA | NA | NA | NA | NA | NA |
| RGS12 | 6002 | NA | NA | NA | NA | NA | NA |
| FCRL5 | 83416 | NA | NA | NA | NA | NA | NA |
| BLMH | 642 | NA | NA | NA | NA | NA | NA |
| NUCB1 | 4924 | NA | NA | NA | NA | NA | NA |
| CHI3L1 | 1116 | NA | NA | NA | NA | NA | NA |
