# Supplementary material for: Candidate Proteins, Metabolites and Transcripts in the Biomarkers for Spinal Muscular Atrophy (BforSMA) Clinical Study
Source: PLoS One. 2012 Apr 27;7(4):e35462. doi: 10.1371/journal.pone.0035462 (PMC3338723; doi:10.1371/journal.pone.0035462)
Supplement: Table S4 — Plasma metabolite analysis against the MHFMS. Analytes described as “unknown” indicate metabolites that could not be identified and there was low confidence of predicting the correct analyte given the acquired structural information; AAA – Amino acid analysis; FFA – Free fatty acid; Q-VALUE – significance corrected for the effect of multiple comparisons; STD – Standard deviation; UCL – Upper 95% confidence limit; LCL – lower 95% confidence limit (on the value of slope); NA – Analytes could not be identified, no assessment made. (DOC) [file pone.0035462.s004.doc]

**Supplementary Table S4. Plasma metabolite analysis against the MHFMS**

| **Platform** | **Analyte Name** | **Slope** | **Std** | **LCL** | **UCL** | **P-value** | **Q-value** |
| --- | --- | --- | --- | --- | --- | --- | --- |
| AAA | Glu | -22.86 | 2.76 | -28.34 | -17.38 | 4.86E-13 | 9.73E-11 |
| GC/MS | L-Glutamic acid | -21.10 | 2.57 | -26.19 | -16.01 | 6.29E-13 | 9.73E-11 |
| GC/MS | L-Aspartic-acid | -25.46 | 3.58 | -32.56 | -18.37 | 1.48E-10 | 1.53E-08 |
| GC/MS | C10:0-fatty-acid | -7.47 | 1.34 | -10.14 | -4.81 | 2.16E-07 | 1.67E-05 |
| AAA | Asp | -17.76 | 3.27 | -24.25 | -11.27 | 3.82E-07 | 2.12E-05 |
| Lipid | 24:1 (major isomer) SM (d18:1) | -7.88 | 1.46 | -10.78 | -4.98 | 4.60E-07 | 2.12E-05 |
| FFA | C10:0 (capric) | -6.47 | 1.20 | -8.86 | -4.08 | 4.80E-07 | 2.12E-05 |
| AAA | PEtN | -16.04 | 3.16 | -22.31 | -9.78 | 1.69E-06 | 6.54E-05 |
| GC/MS | o-Phosphorylethanolamine | -16.91 | 3.58 | -24.02 | -9.81 | 7.42E-06 | 2.55E-04 |
| FFA | C8:0 (caprylic) | -5.48 | 1.17 | -7.80 | -3.16 | 8.72E-06 | 2.69E-04 |
| GC/MS | unknown 31944 uk 08 | 25.44 | 5.73 | 14.09 | 36.80 | 2.23E-05 | 6.28E-04 |
| GC/MS | 2-Ketoglutaric acid | -22.77 | 5.17 | -33.02 | -12.53 | 2.56E-05 | 6.58E-04 |
| AAA | Tau | -18.28 | 4.17 | -26.54 | -10.02 | 2.78E-05 | 6.60E-04 |
| Lipid | 15:1/18:2 PC | 9.55 | 2.12 | 5.31 | 13.79 | 3.39E-05 | 7.48E-04 |
| GC/MS | Glycerol | -10.78 | 2.50 | -15.74 | -5.81 | 3.81E-05 | 7.84E-04 |
| GC/MS | Hypoxanthine | -12.57 | 2.94 | -18.40 | -6.74 | 4.27E-05 | 8.26E-04 |
| FFA | c-C20:1w9 (c-11-eicosenoic) | -15.41 | 3.59 | -22.55 | -8.28 | 4.75E-05 | 8.64E-04 |
| GC/MS | Beta-Alanine | 9.03 | 2.16 | 4.74 | 13.32 | 6.30E-05 | 1.08E-03 |
| GC/MS | unknown 32006\01.08.02 uk x 60 | 8.25 | 2.02 | 4.24 | 12.26 | 8.81E-05 | 1.43E-03 |
| GC/MS | 2-Hydroxybutanoic acid | -13.15 | 3.34 | -19.77 | -6.52 | 1.51E-04 | 2.33E-03 |
| FFA | c-C18:3w3 (linolenic) | -8.97 | 2.33 | -13.59 | -4.34 | 2.07E-04 | 3.05E-03 |
| GC/MS | Sum|Sphingomyeline (N-base: sphingosine|fatty acid C24:0and C24:1) | -20.95 | 5.55 | -31.97 | -9.94 | 2.70E-04 | 3.79E-03 |
| Lipid | 15:1/20:4 PC | 6.25 | 1.74 | 2.79 | 9.71 | 5.50E-04 | 7.39E-03 |
| FFA | c-C18:1w7 (vaccenic) | -10.66 | 3.00 | -16.61 | -4.71 | 5.78E-04 | 7.45E-03 |
| FFA | c-C16:1w10 (sapienic) | -14.15 | 4.02 | -22.11 | -6.18 | 6.38E-04 | 7.89E-03 |
| AAA | 3MHis | -18.55 | 5.35 | -29.16 | -7.93 | 7.73E-04 | 9.13E-03 |
| AAA | Cit | 19.04 | 5.51 | 8.11 | 29.97 | 7.98E-04 | 9.13E-03 |
| GC/MS | Acetoacetate | -4.57 | 1.37 | -7.28 | -1.86 | 1.14E-03 | 1.19E-02 |
| GC/MS | Sphingomyeline (N-base: sphingosine|fatty acid C17:0) | 19.21 | 5.74 | 7.82 | 30.60 | 1.15E-03 | 1.19E-02 |
| GC/MS | 3-Phosphoglyceric acid | -8.01 | 2.40 | -12.76 | -3.26 | 1.15E-03 | 1.19E-02 |
| GC/MS | Sphingomyeline (N-base: hexadecasphing-4-enine|fatty acid C24:1) | -14.36 | 4.32 | -22.93 | -5.79 | 1.24E-03 | 1.23E-02 |
| GC/MS | Myo-inositol | -17.21 | 5.23 | -27.58 | -6.84 | 1.37E-03 | 1.32E-02 |
| GC/MS | unknown 32006/01.07.02 uk x 21 | 10.67 | 3.33 | 4.06 | 17.29 | 1.83E-03 | 1.62E-02 |
| AAA | 1MHis | 3.36 | 1.05 | 1.27 | 5.44 | 1.85E-03 | 1.62E-02 |
| Lipid | 18:2 (minor isomer) LPC | 6.39 | 1.99 | 2.43 | 10.35 | 1.90E-03 | 1.62E-02 |
| AAA | Hyp | 14.21 | 4.47 | 5.35 | 23.07 | 1.94E-03 | 1.62E-02 |
| GC/MS | 3-Hydroxybutanoic acid | -3.07 | 0.97 | -4.99 | -1.15 | 2.00E-03 | 1.62E-02 |
| GC/MS | L-4-Hydroxyproline | 13.05 | 4.12 | 4.88 | 21.22 | 2.02E-03 | 1.62E-02 |
| GC/MS | C18:1-fatty-acid | -4.95 | 1.56 | -8.05 | -1.85 | 2.04E-03 | 1.62E-02 |
| Lipid | 18:2 (major isomer) LPC | 5.46 | 1.77 | 1.94 | 8.98 | 2.66E-03 | 2.04E-02 |
| FFA | c-C18:1w9 (oleic) | -8.91 | 2.90 | -14.67 | -3.16 | 2.71E-03 | 2.04E-02 |
| Lipid | 16:0/20:5 PC | -3.35 | 1.09 | -5.54 | -1.16 | 3.24E-03 | 2.39E-02 |
| Lipid | 18:0 SM (d18:1) | -6.05 | 2.03 | -10.08 | -2.02 | 3.66E-03 | 2.63E-02 |
| Lipid | 15:0/20:4 PC | 4.93 | 1.67 | 1.62 | 8.24 | 3.93E-03 | 2.76E-02 |
| GC/MS | Citric-acid | 13.93 | 4.79 | 4.44 | 23.42 | 4.41E-03 | 3.03E-02 |
| FFA | C6:0 (caproic) | -15.22 | 5.27 | -25.68 | -4.76 | 4.75E-03 | 3.19E-02 |
| GC/MS | Vitamin E | -16.17 | 5.78 | -27.63 | -4.70 | 6.15E-03 | 4.05E-02 |
| GC/MS | 1-Methylhistidine | 4.34 | 1.61 | 1.14 | 7.54 | 8.38E-03 | 5.40E-02 |
| GC/MS | L-Serine | -16.65 | 6.26 | -29.07 | -4.24 | 9.05E-03 | 5.62E-02 |
| Lipid | 16:0/16:1 PC | -3.95 | 1.48 | -6.89 | -1.01 | 9.10E-03 | 5.62E-02 |
| Lipid | 18:0/22:6 PC | -3.13 | 1.19 | -5.49 | -0.77 | 9.85E-03 | 5.97E-02 |
| GC/MS | 2-Hydroxypiperidine | -10.74 | 4.13 | -18.93 | -2.54 | 1.07E-02 | 6.37E-02 |
| GC/MS | Urea | 10.72 | 4.14 | 2.52 | 18.93 | 1.10E-02 | 6.39E-02 |
| Lipid | 18:0/22:5 PC | -4.16 | 1.60 | -7.36 | -0.97 | 1.14E-02 | 6.53E-02 |
| FFA | c-C20:5w3 (EPA) | -9.99 | 3.85 | -17.68 | -2.29 | 1.18E-02 | 6.61E-02 |
| Lipid | 18:3 CE | -2.84 | 1.11 | -5.05 | -0.63 | 1.22E-02 | 6.76E-02 |
| FFA | 17Me-C18:0 (iso) | -9.50 | 3.79 | -17.02 | -1.97 | 1.40E-02 | 7.61E-02 |
| AAA | Ser | -18.57 | 7.67 | -33.78 | -3.36 | 1.72E-02 | 9.16E-02 |
| GC/MS | Sphingomyeline (N-base: sphingosine|fatty acid C22:0) | 15.95 | 6.63 | 2.81 | 29.09 | 1.78E-02 | 9.34E-02 |
| Lipid | 16:0/18:2 PC | 11.23 | 4.79 | 1.74 | 20.72 | 2.09E-02 | 1.06E-01 |
| GC/MS | Sphingomyeline (N-base: sphingosine|fatty acid C23:0) | 12.48 | 5.32 | 1.93 | 23.04 | 2.09E-02 | 1.06E-01 |
| AAA | Arg | 12.37 | 5.29 | 1.88 | 22.85 | 2.13E-02 | 1.06E-01 |
| FFA | C7:0 (enanthic) | -8.80 | 3.91 | -16.58 | -1.03 | 2.70E-02 | 1.33E-01 |
| Lipid | 16:1 CE | -2.73 | 1.23 | -5.17 | -0.29 | 2.90E-02 | 1.40E-01 |
| Lipid | 17:0/20:4 PC | 7.01 | 3.15 | 0.67 | 13.35 | 3.09E-02 | 1.47E-01 |
| GC/MS | d-Mannose | -17.08 | 7.85 | -32.65 | -1.51 | 3.19E-02 | 1.49E-01 |
| FFA | unknown (C19:2?) | -10.65 | 4.90 | -20.43 | -0.87 | 3.33E-02 | 1.52E-01 |
| AAA | Asn | 13.66 | 6.34 | 1.09 | 26.22 | 3.34E-02 | 1.52E-01 |
| GC/MS | Sphingomyeline (N-base: heptadecasphing-4-enine|fatty acid C24:0)? | -10.74 | 5.07 | -20.80 | -0.68 | 3.66E-02 | 1.64E-01 |
| Lipid | 22:1 SM (d18:1) | 4.70 | 2.23 | 0.27 | 9.13 | 3.77E-02 | 1.66E-01 |
| GC/MS | L-Asparagine | 10.08 | 4.82 | 0.53 | 19.63 | 3.87E-02 | 1.68E-01 |
| GC/MS | L-Threonine | -8.83 | 4.34 | -17.43 | -0.23 | 4.44E-02 | 1.90E-01 |
| Lipid | 20:0 SM (d18:1) | -6.78 | 3.32 | -13.40 | -0.16 | 4.49E-02 | 1.90E-01 |
| FFA | C11:0 (undecanoic) | -3.59 | 1.79 | -7.15 | -0.04 | 4.73E-02 | 1.97E-01 |
| AAA | Aad | -9.12 | 4.58 | -18.20 | -0.04 | 4.89E-02 | 1.97E-01 |
| GC/MS | L(-)-Malic-acid | -11.18 | 5.61 | -22.31 | -0.05 | 4.89E-02 | 1.97E-01 |
| GC/MS | unknown 52 | 9.34 | 4.69 | 0.04 | 18.64 | 4.91E-02 | 1.97E-01 |
| GC/MS | unknown P7502_UK02 | 8.39 | 4.26 | -0.06 | 16.83 | 5.16E-02 | 2.02E-01 |
| Lipid | 16:1/18:2 PC | -2.74 | 1.39 | -5.51 | 0.02 | 5.19E-02 | 2.02E-01 |
| FFA | 15Me-C16:0 (iso) | 4.94 | 2.51 | -0.05 | 9.93 | 5.23E-02 | 2.02E-01 |
| GC/MS | Sphingomyeline (N-base: sphingosine|fatty acid C16:0) | 14.66 | 7.51 | -0.24 | 29.57 | 5.37E-02 | 2.05E-01 |
| GC/MS | unknown 61 | 13.09 | 6.74 | -0.29 | 26.46 | 5.50E-02 | 2.07E-01 |
| Lipid | 18:2 CE | 16.81 | 8.68 | -0.40 | 34.02 | 5.55E-02 | 2.07E-01 |
| GC/MS | Indole-3-propionic acid | 3.20 | 1.66 | -0.09 | 6.50 | 5.68E-02 | 2.09E-01 |
| GC/MS | unknown P7881_uk 21 | -10.49 | 5.49 | -21.38 | 0.39 | 5.87E-02 | 2.12E-01 |
| GC/MS | unknown P7881_uk 01 | 6.83 | 3.57 | -0.26 | 13.92 | 5.90E-02 | 2.12E-01 |
| GC/MS | C16:1-fatty-acid | -3.01 | 1.60 | -6.19 | 0.16 | 6.26E-02 | 2.22E-01 |
| AAA | Cys | -13.23 | 7.08 | -27.28 | 0.82 | 6.46E-02 | 2.27E-01 |
| GC/MS | unknown 39b | -7.44 | 4.01 | -15.40 | 0.52 | 6.67E-02 | 2.32E-01 |
| Lipid | 18:2/18:2/16:0 TG | 2.05 | 1.13 | -0.19 | 4.29 | 7.22E-02 | 2.44E-01 |
| Lipid | 16:0 (minor isomer) LPC | 3.91 | 2.16 | -0.36 | 8.18 | 7.25E-02 | 2.44E-01 |
| Lipid | 20:3 CE | -3.18 | 1.75 | -6.66 | 0.30 | 7.26E-02 | 2.44E-01 |
| GC/MS | 1,2-Diglyceride|Molweight not derivatised = 592 | 10.73 | 5.94 | -1.04 | 22.51 | 7.36E-02 | 2.45E-01 |
| GC/MS | Maltose | -5.68 | 3.16 | -11.95 | 0.60 | 7.57E-02 | 2.49E-01 |
| GC/MS | C16:0-fatty-acid | -5.06 | 2.87 | -10.75 | 0.63 | 8.09E-02 | 2.63E-01 |
| FFA | c-C14:1w5 (myristoleic) | -4.41 | 2.53 | -9.42 | 0.61 | 8.43E-02 | 2.71E-01 |
| FFA | cis-C22:6w3 (DHA, cervonic) | -5.63 | 3.25 | -12.07 | 0.80 | 8.56E-02 | 2.73E-01 |
| AAA | Thr | -9.05 | 5.24 | -19.43 | 1.34 | 8.70E-02 | 2.74E-01 |
| GC/MS | C18:0-fatty-acid | -6.73 | 3.94 | -14.55 | 1.08 | 9.06E-02 | 2.80E-01 |
| AAA | Hcit | -4.72 | 2.77 | -10.21 | 0.76 | 9.06E-02 | 2.80E-01 |
| FFA | c-C16:1w7 (palmitoleic) | -3.79 | 2.26 | -8.27 | 0.69 | 9.64E-02 | 2.94E-01 |
| GC/MS | L-Histidine | 5.96 | 3.56 | -1.10 | 13.03 | 9.71E-02 | 2.94E-01 |
| AAA | Pro | -7.41 | 4.56 | -16.45 | 1.64 | 1.07E-01 | 3.22E-01 |
| GC/MS | D-Ribulose or D- Xylulose | 10.73 | 6.66 | -2.47 | 23.94 | 1.10E-01 | 3.27E-01 |
| FFA | 14Me-C15:0 (iso) | 3.53 | 2.21 | -0.84 | 7.91 | 1.12E-01 | 3.31E-01 |
| Lipid | 16:0 (major isomer) LPC | 4.78 | 3.02 | -1.22 | 10.78 | 1.17E-01 | 3.41E-01 |
| GC/MS | unknown 61b | 5.01 | 3.18 | -1.30 | 11.32 | 1.18E-01 | 3.42E-01 |
| GC/MS | unknown 32006\01.08.02 uk x 10 | 6.65 | 4.24 | -1.76 | 15.06 | 1.20E-01 | 3.43E-01 |
| GC/MS | Sphingomyeline (N-base: heptadecasphing-4-enine|fatty acid C16:0) | 6.80 | 4.35 | -1.83 | 15.44 | 1.21E-01 | 3.44E-01 |
| GC/MS | free Phosphate | -11.55 | 7.54 | -26.50 | 3.40 | 1.29E-01 | 3.61E-01 |
| Lipid | 18:2/18:1/16:0 TG | 2.02 | 1.33 | -0.62 | 4.65 | 1.32E-01 | 3.67E-01 |
| GC/MS | unknown P7881_uk 11 | 7.93 | 5.27 | -2.51 | 18.38 | 1.35E-01 | 3.68E-01 |
| GC/MS | L-Proline | -5.37 | 3.57 | -12.45 | 1.70 | 1.35E-01 | 3.68E-01 |
| GC/MS | 4-hydroxyglutamate semialdehyde | 10.16 | 6.76 | -3.24 | 23.56 | 1.36E-01 | 3.68E-01 |
| Lipid | 18:0/20:3 PC | -2.27 | 1.54 | -5.31 | 0.78 | 1.43E-01 | 3.83E-01 |
| FFA | 12Me-C13:0 (iso-myristic) | -5.69 | 3.85 | -13.36 | 1.99 | 1.44E-01 | 3.83E-01 |
| FFA | c-C22:5w3 (DPA) | -7.49 | 5.08 | -17.64 | 2.67 | 1.46E-01 | 3.85E-01 |
| GC/MS | unknown 81 | 6.12 | 4.31 | -2.42 | 14.67 | 1.58E-01 | 4.12E-01 |
| AAA | His | 13.37 | 9.42 | -5.30 | 32.05 | 1.59E-01 | 4.12E-01 |
| GC/MS | unknown 52b | 7.59 | 5.42 | -3.16 | 18.34 | 1.65E-01 | 4.24E-01 |
| GC/MS | Nicotinamide | -10.44 | 7.48 | -25.27 | 4.40 | 1.66E-01 | 4.24E-01 |
| GC/MS | C22:6-fatty acid | -4.87 | 3.51 | -11.84 | 2.09 | 1.68E-01 | 4.24E-01 |
| GC/MS | Fumaric-acid | -8.73 | 6.30 | -21.22 | 3.76 | 1.69E-01 | 4.24E-01 |
| Lipid | 18:1/18:2 PC | 3.20 | 2.32 | -1.40 | 7.80 | 1.70E-01 | 4.24E-01 |
| GC/MS | Sarcosine | 8.11 | 5.95 | -3.68 | 19.91 | 1.75E-01 | 4.33E-01 |
| GC/MS | N-carboxy-alanine | 3.54 | 2.60 | -1.62 | 8.71 | 1.76E-01 | 4.33E-01 |
| GC/MS | C18:2-fatty-acid | -3.68 | 2.74 | -9.11 | 1.76 | 1.82E-01 | 4.41E-01 |
| GC/MS | 4-Deoxyglucose | -11.11 | 8.31 | -27.60 | 5.38 | 1.85E-01 | 4.41E-01 |
| AAA | Hcy | -23.33 | 17.47 | -57.98 | 11.33 | 1.85E-01 | 4.41E-01 |
| GC/MS | unknown 31944 uk 11 | 6.42 | 4.82 | -3.14 | 15.97 | 1.86E-01 | 4.41E-01 |
| Lipid | 18:0/18:1 PC | -2.31 | 1.76 | -5.80 | 1.17 | 1.91E-01 | 4.50E-01 |
| GC/MS | L-Tryptophan | 5.29 | 4.05 | -2.74 | 13.32 | 1.95E-01 | 4.56E-01 |
| Lipid | 18:1 SM (d18:1) | -3.46 | 2.67 | -8.78 | 1.86 | 1.99E-01 | 4.61E-01 |
| FFA | C9:0 (pelargic) | -4.92 | 3.81 | -12.48 | 2.64 | 2.00E-01 | 4.61E-01 |
| AAA | Trp | 7.93 | 6.18 | -4.33 | 20.19 | 2.02E-01 | 4.63E-01 |
| Lipid | 16:0/22:6 PC | -1.89 | 1.48 | -4.83 | 1.05 | 2.05E-01 | 4.66E-01 |
| GC/MS | 1,3-Diglyceride|Molweight not derivatised = 592 | 6.40 | 5.08 | -3.67 | 16.48 | 2.10E-01 | 4.75E-01 |
| Lipid | 16:0/20:4 PC | 2.82 | 2.28 | -1.71 | 7.35 | 2.20E-01 | 4.92E-01 |
| GC/MS | L-Alanine | 5.68 | 4.65 | -3.54 | 14.91 | 2.25E-01 | 4.98E-01 |
| Lipid | 16:0 SM (d18:1) | 4.19 | 3.45 | -2.66 | 11.04 | 2.28E-01 | 4.98E-01 |
| GC/MS | Threonic acid | -3.67 | 3.03 | -9.67 | 2.33 | 2.28E-01 | 4.98E-01 |
| AAA | Sar | 7.35 | 6.07 | -4.69 | 19.38 | 2.29E-01 | 4.98E-01 |
| FFA | C19:0 (nonadecanoic) | -8.32 | 6.88 | -22.12 | 5.48 | 2.32E-01 | 5.01E-01 |
| FFA | C16:0 (palmitic) | -5.37 | 4.55 | -14.39 | 3.66 | 2.41E-01 | 5.17E-01 |
| GC/MS | Sucrose | 4.28 | 3.65 | -2.96 | 11.52 | 2.44E-01 | 5.20E-01 |
| GC/MS | unknown 31944 uk 12 | -10.20 | 8.75 | -27.55 | 7.16 | 2.47E-01 | 5.22E-01 |
| Lipid | 15:0/18:2 PC | 4.78 | 4.10 | -3.61 | 13.17 | 2.54E-01 | 5.31E-01 |
| FFA | c-C22:4w6 (adrenic) | -11.34 | 9.85 | -31.23 | 8.55 | 2.56E-01 | 5.31E-01 |
| AAA | Tyr | 5.00 | 4.42 | -3.77 | 13.77 | 2.61E-01 | 5.31E-01 |
| AAA | Ala | 6.06 | 5.37 | -4.59 | 16.71 | 2.62E-01 | 5.31E-01 |
| GC/MS | Sphingomyeline (N-base: sphingadiene|fatty acid C16:0) | 8.27 | 7.34 | -6.29 | 22.82 | 2.62E-01 | 5.31E-01 |
| GC/MS | unknown S7010 ukx08 | -9.51 | 8.46 | -26.28 | 7.26 | 2.64E-01 | 5.31E-01 |
| GC/MS | Sphingomyeline (N-base: hexadecasphing-4-enine|fatty acid C22:0) | 4.75 | 4.23 | -3.65 | 13.15 | 2.65E-01 | 5.31E-01 |
| GC/MS | unknown 49 | -5.12 | 4.58 | -14.20 | 3.96 | 2.66E-01 | 5.31E-01 |
| FFA | c-C20:4w6 (arachidonic) | 6.62 | 5.94 | -5.16 | 18.41 | 2.68E-01 | 5.31E-01 |
| GC/MS | Sphingomyeline (N-base: heptadecasphing-4-enine|fatty acid C18:0) | 4.43 | 3.98 | -3.46 | 12.31 | 2.68E-01 | 5.31E-01 |
| GC/MS | Oxalic acid | 10.02 | 9.07 | -7.97 | 28.01 | 2.72E-01 | 5.35E-01 |
| GC/MS | Glycine | 6.40 | 5.83 | -5.17 | 17.97 | 2.75E-01 | 5.38E-01 |
| GC/MS | Sphingomyeline (N-base: hexadecasphing-4-enine|fatty acid C20:0) | -5.03 | 4.60 | -14.15 | 4.09 | 2.77E-01 | 5.38E-01 |
| AAA | EtN | 4.50 | 4.17 | -3.76 | 12.77 | 2.82E-01 | 5.45E-01 |
| GC/MS | Succinic acid | 4.65 | 4.35 | -3.98 | 13.29 | 2.88E-01 | 5.52E-01 |
| FFA | C15:0 (pentadecanoic) | 6.26 | 5.89 | -5.41 | 17.94 | 2.90E-01 | 5.53E-01 |
| GC/MS | Iminodiacetic acid | 3.59 | 3.40 | -3.15 | 10.33 | 2.93E-01 | 5.55E-01 |
| GC/MS | sn-Glycerol-3-phosphate | -6.33 | 6.01 | -18.25 | 5.58 | 2.94E-01 | 5.55E-01 |
| GC/MS | Aminomalonic-acid | 3.91 | 3.74 | -3.50 | 11.32 | 2.98E-01 | 5.58E-01 |
| Lipid | 24:1 (minor isomer) SM (d18:1) | 4.86 | 4.63 | -4.58 | 14.31 | 3.01E-01 | 5.61E-01 |
| Lipid | 18:2/16:0/14:0 TG | 1.18 | 1.15 | -1.13 | 3.49 | 3.09E-01 | 5.71E-01 |
| FFA | C18:0 (stearic) | -4.72 | 4.75 | -14.15 | 4.70 | 3.23E-01 | 5.93E-01 |
| Lipid | 18:2/16:0/16:0 TG | 0.81 | 0.82 | -0.81 | 2.43 | 3.24E-01 | 5.93E-01 |
| GC/MS | L-Tyrosine | 3.12 | 3.17 | -3.17 | 9.41 | 3.27E-01 | 5.95E-01 |
| Lipid | 18:0 (major isomer) LPC | 2.19 | 2.25 | -2.27 | 6.66 | 3.32E-01 | 5.98E-01 |
| Lipid | 22:0 SM (d18:1) | 2.19 | 2.25 | -2.28 | 6.65 | 3.33E-01 | 5.98E-01 |
| FFA | 13Me-C14:0 (iso) | -2.77 | 2.91 | -8.55 | 3.00 | 3.43E-01 | 6.03E-01 |
| GC/MS | Inositol | 2.13 | 2.23 | -2.31 | 6.56 | 3.43E-01 | 6.03E-01 |
| GC/MS | C14:0-fatty-acid | -2.06 | 2.16 | -6.34 | 2.22 | 3.43E-01 | 6.03E-01 |
| Lipid | 16:0 SM (d18:0) | -3.62 | 3.78 | -11.22 | 3.98 | 3.43E-01 | 6.03E-01 |
| GC/MS | S-methyl-L-cysteýne | 3.97 | 4.19 | -4.34 | 12.28 | 3.46E-01 | 6.04E-01 |
| GC/MS | Arachidonic acid | 5.56 | 5.92 | -6.18 | 17.30 | 3.49E-01 | 6.04E-01 |
| Lipid | 24:2 SM (d18:1) | 2.01 | 2.13 | -2.27 | 6.30 | 3.50E-01 | 6.04E-01 |
| FFA | c-C16:1w5 (c-11-hexadecenoic) | -4.65 | 4.98 | -14.56 | 5.25 | 3.53E-01 | 6.05E-01 |
| Lipid | 16:0 CE | 3.48 | 3.74 | -3.93 | 10.88 | 3.54E-01 | 6.05E-01 |
| GC/MS | Sum|Sphingomyeline (N-base: sphingadiene|fatty acid C24:0and C24:1) | -6.13 | 6.83 | -19.68 | 7.42 | 3.72E-01 | 6.32E-01 |
| GC/MS | unknown 60a | -8.09 | 9.20 | -26.33 | 10.16 | 3.81E-01 | 6.44E-01 |
| Lipid | 18:1/18:1/18:1 TG | -0.78 | 0.92 | -2.60 | 1.04 | 3.96E-01 | 6.65E-01 |
| GC/MS | unknown 39d | -4.50 | 5.45 | -15.30 | 6.30 | 4.10E-01 | 6.85E-01 |
| GC/MS | L-Valine | -3.49 | 4.29 | -12.00 | 5.02 | 4.18E-01 | 6.95E-01 |
| GC/MS | L-Lysine | 3.26 | 4.22 | -5.11 | 11.62 | 4.42E-01 | 7.30E-01 |
| GC/MS | EDTA | 7.00 | 9.25 | -11.34 | 25.34 | 4.51E-01 | 7.38E-01 |
| GC/MS | D-Glucose | -7.55 | 9.99 | -27.37 | 12.27 | 4.52E-01 | 7.38E-01 |
| Lipid | 23:0 SM (d18:1) | -3.68 | 4.88 | -13.56 | 6.19 | 4.55E-01 | 7.40E-01 |
| GC/MS | L-Isoleucine | -2.58 | 3.50 | -9.52 | 4.36 | 4.63E-01 | 7.49E-01 |
| Lipid | 16:0/18:1 PC | -1.72 | 2.39 | -6.45 | 3.01 | 4.72E-01 | 7.60E-01 |
| Lipid | 18:2/18:2/18:1 TG | 0.59 | 0.82 | -1.03 | 2.21 | 4.74E-01 | 7.60E-01 |
| GC/MS | unknown 10227\01.03 uk x 25 | -3.94 | 5.53 | -14.91 | 7.03 | 4.78E-01 | 7.61E-01 |
| GC/MS | unknown P7881_uk 48 | -7.97 | 11.48 | -30.72 | 14.79 | 4.89E-01 | 7.73E-01 |
| Lipid | 16:0/16:0 PC | 1.77 | 2.56 | -3.30 | 6.85 | 4.90E-01 | 7.73E-01 |
| Lipid | 20:4 CE | 1.90 | 2.80 | -3.64 | 7.44 | 4.98E-01 | 7.82E-01 |
| AAA | Gly | 4.64 | 7.04 | -9.32 | 18.60 | 5.11E-01 | 7.98E-01 |
| Lipid | 18:0/18:2 PC | 1.66 | 2.53 | -3.36 | 6.67 | 5.14E-01 | 7.98E-01 |
| Lipid | 33:1 PC | -6.78 | 10.31 | -28.22 | 14.66 | 5.18E-01 | 7.99E-01 |
| GC/MS | N-Carboxyproline | -1.84 | 2.85 | -7.50 | 3.81 | 5.20E-01 | 7.99E-01 |
| FFA | C12:0 (lauric) | 1.04 | 1.62 | -2.19 | 4.26 | 5.25E-01 | 8.03E-01 |
| AAA | Val | -3.59 | 5.70 | -14.89 | 7.70 | 5.30E-01 | 8.06E-01 |
| Lipid | 18:1/18:0/16:0 TG | 0.50 | 0.81 | -1.11 | 2.11 | 5.41E-01 | 8.20E-01 |
| GC/MS | L-Cysteýne | -2.81 | 4.62 | -11.97 | 6.36 | 5.45E-01 | 8.21E-01 |
| AAA | Pser | 7.39 | 12.33 | -17.06 | 31.85 | 5.50E-01 | 8.24E-01 |
| GC/MS | 1-Mono-oleoylglycerol | -2.18 | 3.66 | -9.44 | 5.08 | 5.53E-01 | 8.24E-01 |
| GC/MS | L-Phenylalanine | -3.05 | 5.16 | -13.28 | 7.18 | 5.56E-01 | 8.24E-01 |
| GC/MS | 1-Mono-stearoylglycerol | -4.13 | 7.02 | -18.06 | 9.80 | 5.58E-01 | 8.24E-01 |
| AAA | Ile | -2.50 | 4.27 | -10.96 | 5.96 | 5.60E-01 | 8.24E-01 |
| GC/MS | 1-Palmitoyl-sn-glycero-3-phosphocholine | -2.04 | 3.54 | -9.06 | 4.98 | 5.66E-01 | 8.26E-01 |
| GC/MS | Sphingomyeline (N-base: sphingadiene|fatty acid C18:0) | 2.80 | 4.88 | -6.88 | 12.47 | 5.68E-01 | 8.26E-01 |
| GC/MS | Creatinine | -2.88 | 5.04 | -12.87 | 7.11 | 5.69E-01 | 8.26E-01 |
| Lipid | 17:1/20:4 PC | 1.16 | 2.10 | -3.01 | 5.32 | 5.83E-01 | 8.39E-01 |
| GC/MS | D-Xylose | 1.04 | 1.89 | -2.71 | 4.79 | 5.84E-01 | 8.39E-01 |
| GC/MS | Fructose | 0.64 | 1.19 | -1.73 | 3.01 | 5.94E-01 | 8.49E-01 |
| GC/MS | 1,3-Diglyceride|Molweight not derivatised = 620 | 3.02 | 5.72 | -8.33 | 14.37 | 5.99E-01 | 8.50E-01 |
| FFA | c-C18:3w6 (gamma-linolenic) | -1.77 | 3.36 | -8.43 | 4.90 | 6.00E-01 | 8.50E-01 |
| GC/MS | Hexadecanoic-methylester | 2.46 | 4.71 | -6.88 | 11.79 | 6.03E-01 | 8.50E-01 |
| GC/MS | N-acetylaminomalonic acid | 1.19 | 2.31 | -3.40 | 5.77 | 6.09E-01 | 8.56E-01 |
| Lipid | 22:6 CE | -0.91 | 1.79 | -4.48 | 2.66 | 6.12E-01 | 8.56E-01 |
| Lipid | Unidentified | 1.02 | 2.03 | -3.02 | 5.05 | 6.18E-01 | 8.59E-01 |
| GC/MS | 4-Methyl-2-oxovaleric-acid | 2.18 | 4.38 | -6.52 | 10.87 | 6.21E-01 | 8.59E-01 |
| GC/MS | Cholesterol | 2.58 | 5.23 | -7.79 | 12.95 | 6.23E-01 | 8.59E-01 |
| AAA | Gln | 5.93 | 12.11 | -18.08 | 29.94 | 6.25E-01 | 8.59E-01 |
| GC/MS | unknown S7010 ukx11 | -0.92 | 1.93 | -4.74 | 2.90 | 6.33E-01 | 8.62E-01 |
| GC/MS | unknown 59c | -1.62 | 3.39 | -8.35 | 5.11 | 6.34E-01 | 8.62E-01 |
| FFA | C13:0 (tridecanoic) | -1.67 | 3.60 | -8.83 | 5.49 | 6.44E-01 | 8.72E-01 |
| GC/MS | 1,2-Diglyceride|Molweight not derivatised = 620 | -2.43 | 5.28 | -12.92 | 8.05 | 6.46E-01 | 8.72E-01 |
| GC/MS | L-Glutamine | 2.27 | 4.97 | -7.59 | 12.12 | 6.49E-01 | 8.72E-01 |
| Lipid | 18:1 CE | -1.89 | 4.27 | -10.35 | 6.57 | 6.58E-01 | 8.76E-01 |
| GC/MS | unknown 42 | -2.01 | 4.57 | -11.08 | 7.06 | 6.61E-01 | 8.76E-01 |
| GC/MS | 4-hydroxyglutamate semialdehyde | 3.54 | 8.15 | -12.62 | 19.69 | 6.65E-01 | 8.76E-01 |
| Lipid | 16:1 SM (d18:1) | -1.20 | 2.76 | -6.68 | 4.29 | 6.66E-01 | 8.76E-01 |
| GC/MS | unknown P7478_UK09 | -1.06 | 2.47 | -5.96 | 3.84 | 6.68E-01 | 8.76E-01 |
| FFA | c-C18:2w6 (linoleic) | -2.23 | 5.19 | -12.52 | 8.07 | 6.69E-01 | 8.76E-01 |
| FFA | C14:0 (myristic) | -1.12 | 2.64 | -6.36 | 4.12 | 6.73E-01 | 8.78E-01 |
| Lipid | 20:4 (major isomer) LPC | 1.03 | 2.51 | -3.97 | 6.03 | 6.83E-01 | 8.81E-01 |
| GC/MS | Pyrophosphate | 2.27 | 5.60 | -8.85 | 13.38 | 6.86E-01 | 8.81E-01 |
| GC/MS | 1-Palmitoyl-L-alpha-lysophosphatidic acid | 1.98 | 4.92 | -7.77 | 11.73 | 6.88E-01 | 8.81E-01 |
| GC/MS | D-Glyceric acid | -2.47 | 6.22 | -14.80 | 9.86 | 6.92E-01 | 8.81E-01 |
| FFA | C17:0 (heptadecanoic) | 1.61 | 4.09 | -6.51 | 9.72 | 6.95E-01 | 8.81E-01 |
| GC/MS | Pyruvic acid | -2.42 | 6.20 | -14.72 | 9.89 | 6.98E-01 | 8.81E-01 |
| Lipid | 18:0 (minor isomer) LPC | 2.74 | 6.99 | -11.60 | 17.09 | 6.98E-01 | 8.81E-01 |
| FFA | c-C20:3w6 (c-8,11,14-C20:3) | -1.97 | 5.06 | -12.01 | 8.07 | 6.98E-01 | 8.81E-01 |
| AAA | Lys | -2.00 | 5.37 | -12.65 | 8.65 | 7.10E-01 | 8.89E-01 |
| Lipid | 18:2/16:1/16:0 TG | -0.31 | 0.84 | -1.98 | 1.36 | 7.10E-01 | 8.89E-01 |
| Lipid | 17:0/18:2 PC | 0.90 | 2.47 | -4.02 | 5.81 | 7.18E-01 | 8.94E-01 |
| AAA | bAla | -4.59 | 12.80 | -29.97 | 20.80 | 7.21E-01 | 8.95E-01 |
| GC/MS | Sphingomyeline (N-base: hexadecasphing-4-enine|fatty acid C18:0) | -1.34 | 3.78 | -8.83 | 6.16 | 7.24E-01 | 8.95E-01 |
| GC/MS | unknown 10227\01.03 uk x 20 | -1.74 | 5.20 | -12.06 | 8.58 | 7.39E-01 | 9.09E-01 |
| GC/MS | unknown 60 | 1.68 | 5.28 | -8.80 | 12.16 | 7.51E-01 | 9.18E-01 |
| AAA | bAib | -1.36 | 4.27 | -9.83 | 7.12 | 7.52E-01 | 9.18E-01 |
| Lipid | 18:2/18:1/18:1 TG | 0.28 | 0.95 | -1.61 | 2.17 | 7.67E-01 | 9.34E-01 |
| GC/MS | 1-Linoleoyl-sn-glycero-3-phosphocholine | -0.89 | 3.04 | -6.91 | 5.13 | 7.71E-01 | 9.34E-01 |
| GC/MS | C12:0-fatty-acid | 0.50 | 1.73 | -2.94 | 3.94 | 7.74E-01 | 9.34E-01 |
| GC/MS | unknown 48 | 0.92 | 3.29 | -5.61 | 7.45 | 7.80E-01 | 9.38E-01 |
| FFA | t-C18:1w9 (elaidic) | 0.91 | 3.29 | -5.65 | 7.46 | 7.83E-01 | 9.38E-01 |
| Lipid | 18:3/18:2/16:0 TG | -0.31 | 1.17 | -2.65 | 2.02 | 7.90E-01 | 9.43E-01 |
| FFA | c-C17:1w8 (c-11-heptadecenoic) | 0.89 | 3.40 | -5.86 | 7.63 | 7.95E-01 | 9.45E-01 |
| GC/MS | myo-Inositol 1,2-cyclic phosphate | 1.57 | 6.15 | -10.62 | 13.77 | 7.98E-01 | 9.45E-01 |
| GC/MS | Uric acid | -1.00 | 3.99 | -8.92 | 6.92 | 8.03E-01 | 9.47E-01 |
| Lipid | 18:1/18:1/18:0 TG | -0.24 | 0.98 | -2.20 | 1.72 | 8.10E-01 | 9.52E-01 |
| FFA | c-C18:1w6 (c-12-octadecenoic) | 0.60 | 2.60 | -4.56 | 5.77 | 8.17E-01 | 9.55E-01 |
| GC/MS | L-Ornithine | -1.03 | 4.48 | -9.91 | 7.86 | 8.19E-01 | 9.55E-01 |
| GC/MS | unknown 56 | 0.62 | 2.78 | -4.90 | 6.14 | 8.24E-01 | 9.58E-01 |
| GC/MS | unknown 76 | 0.60 | 2.79 | -4.93 | 6.12 | 8.31E-01 | 9.60E-01 |
| GC/MS | Sphingomyeline (N-base: sphingadiene|fatty acid C20:0) | -1.49 | 7.06 | -15.49 | 12.50 | 8.33E-01 | 9.60E-01 |
| GC/MS | Monomethylphosphate | 0.92 | 4.52 | -8.04 | 9.89 | 8.38E-01 | 9.63E-01 |
| FFA | C20:0 (eicosanoic) | -0.61 | 3.22 | -6.99 | 5.77 | 8.50E-01 | 9.70E-01 |
| Lipid | 18:0/20:4 PC | 0.43 | 2.26 | -4.06 | 4.91 | 8.51E-01 | 9.70E-01 |
| GC/MS | L-Leucine | -0.68 | 3.94 | -8.50 | 7.14 | 8.64E-01 | 9.80E-01 |
| GC/MS | N-methyl-4-hydroxyproline | 0.28 | 1.64 | -3.01 | 3.57 | 8.66E-01 | 9.80E-01 |
| AAA | Phe | -0.86 | 5.63 | -12.03 | 10.32 | 8.79E-01 | 9.88E-01 |
| GC/MS | Sphingomyeline (N-base: dihydrosphingosine|fatty acid C16:0) | 1.04 | 7.06 | -12.96 | 15.05 | 8.83E-01 | 9.88E-01 |
| AAA | Met | 0.63 | 4.49 | -8.26 | 9.53 | 8.88E-01 | 9.88E-01 |
| GC/MS | unknown 32006\01.08.02 uk x 20 | -0.40 | 2.89 | -6.19 | 5.40 | 8.91E-01 | 9.88E-01 |
| GC/MS | unknown 10227\01.03 uk x 40 | 0.35 | 2.70 | -5.00 | 5.70 | 8.97E-01 | 9.88E-01 |
| AAA | Orn | -0.65 | 5.02 | -10.61 | 9.31 | 8.97E-01 | 9.88E-01 |
| Lipid | 20:4/18:2/16:0 TG | 0.13 | 1.09 | -2.05 | 2.31 | 9.06E-01 | 9.88E-01 |
| GC/MS | unknown 31944 uk 02 | -0.45 | 3.87 | -8.12 | 7.21 | 9.07E-01 | 9.88E-01 |
| AAA | Hyl | 0.77 | 6.79 | -12.70 | 14.25 | 9.10E-01 | 9.88E-01 |
| GC/MS | 1-Mono-palmitoylglycerol | 0.81 | 7.18 | -13.42 | 15.05 | 9.10E-01 | 9.88E-01 |
| Lipid | 18:1 (minor isomer) LPC | 0.21 | 2.04 | -3.83 | 4.26 | 9.16E-01 | 9.88E-01 |
| GC/MS | L-Methionine | -0.45 | 4.41 | -9.20 | 8.29 | 9.18E-01 | 9.88E-01 |
| GC/MS | Erythronic acid | 0.65 | 7.63 | -14.49 | 15.79 | 9.32E-01 | 9.88E-01 |
| GC/MS | Sphingomyeline (N-base: sphingosine|fatty acid C18:0) | -0.42 | 5.52 | -11.37 | 10.54 | 9.40E-01 | 9.88E-01 |
| Lipid | 18:1/16:0/14:0 TG | 0.06 | 0.81 | -1.56 | 1.68 | 9.42E-01 | 9.88E-01 |
| AAA | Abu | -0.33 | 4.67 | -9.60 | 8.93 | 9.43E-01 | 9.88E-01 |
| Lipid | 24:0 SM (d18:1) | 0.17 | 2.43 | -4.66 | 5.01 | 9.44E-01 | 9.88E-01 |
| GC/MS | 1,5-Anhydro-D-Glucitol | -0.24 | 3.49 | -7.16 | 6.68 | 9.44E-01 | 9.88E-01 |
| GC/MS | unknown 37b | 0.37 | 5.71 | -10.96 | 11.70 | 9.49E-01 | 9.88E-01 |
| Lipid | 18:1/16:0/16:0 TG | 0.05 | 0.82 | -1.57 | 1.67 | 9.49E-01 | 9.88E-01 |
| Lipid | 16:0/22:5 PC | -0.11 | 1.87 | -3.82 | 3.61 | 9.55E-01 | 9.88E-01 |
| GC/MS | meso-Erythritol | 0.43 | 7.88 | -15.21 | 16.06 | 9.57E-01 | 9.88E-01 |
| Lipid | 16:0/16:0/16:0 TG | 0.05 | 1.04 | -2.04 | 2.14 | 9.59E-01 | 9.88E-01 |
| Lipid | 18:0/16:0/14:0 TG | 0.08 | 1.63 | -3.25 | 3.42 | 9.59E-01 | 9.88E-01 |
| GC/MS | L-Lysine | -0.20 | 3.97 | -8.07 | 7.67 | 9.59E-01 | 9.88E-01 |
| GC/MS | Sphingomyeline (N-base: hexadecasphing-4-enine|fatty acid C16:0) | 0.17 | 3.73 | -7.23 | 7.58 | 9.63E-01 | 9.88E-01 |
| AAA | Leu | 0.19 | 4.59 | -8.90 | 9.29 | 9.67E-01 | 9.88E-01 |
| GC/MS | C17:0-fatty-acid | 0.15 | 3.65 | -7.10 | 7.39 | 9.68E-01 | 9.88E-01 |
| GC/MS | DL-Lactic acid | 0.13 | 3.41 | -6.62 | 6.89 | 9.69E-01 | 9.88E-01 |
| GC/MS | Glutamic acid-internal-amide | 0.32 | 9.59 | -18.70 | 19.34 | 9.74E-01 | 9.88E-01 |
| FFA | 12Me-C14:0 (anteiso) | 0.08 | 2.39 | -4.67 | 4.82 | 9.74E-01 | 9.88E-01 |
| GC/MS | Arabinose | -0.21 | 6.48 | -13.05 | 12.64 | 9.75E-01 | 9.88E-01 |
| GC/MS | unknown 31944 uk 15 | 0.07 | 2.87 | -5.62 | 5.76 | 9.81E-01 | 9.91E-01 |
| Lipid | 20:4/18:1/18:1 TG | -0.04 | 2.27 | -4.58 | 4.51 | 9.87E-01 | 9.94E-01 |
| GC/MS | 1,2,4-Trihydroxybenzene | -0.02 | 2.89 | -5.75 | 5.70 | 9.93E-01 | 9.96E-01 |
| GC/MS | 3-Methyl-2-oxovaleric acid | 0.01 | 5.89 | -11.67 | 11.70 | 9.98E-01 | 9.98E-01 |
| FFA | 18Me-C19:0 (iso-eicosanoic) | NA | NA | NA | NA | NA | NA |
| FFA | c-C18:4w3 (stearidonic) | NA | NA | NA | NA | NA | NA |
| FFA | c-C20:2w6 (c-11,14-eicosadinoic) | NA | NA | NA | NA | NA | NA |
| FFA | c-C20:3w3 (c-11,14,17-C20:3) | NA | NA | NA | NA | NA | NA |
| FFA | C22:0 (behenic) | NA | NA | NA | NA | NA | NA |
| FFA | C24:0 (lignoceric) | NA | NA | NA | NA | NA | NA |
