# Supplementary material for: Candidate Proteins, Metabolites and Transcripts in the Biomarkers for Spinal Muscular Atrophy (BforSMA) Clinical Study
Source: PLoS One. 2012 Apr 27;7(4):e35462. doi: 10.1371/journal.pone.0035462 (PMC3338723; doi:10.1371/journal.pone.0035462)
Supplement: Table S5 — Urine metabolite analysis against the MHFMS. Analytes with XXXX_uk## formats indicate metabolites that could not be identified and there was low confidence of predicting the correct analyte given the acquired structural information; Q-VALUE – significance corrected for the effect of multiple comparisons; STD – Standard deviation; UCL – Upper 95% confidence limit; LCL – lower 95% confidence limit (on the value of slope); NA – Analytes could not be identified, no assessment made. (DOC) [file pone.0035462.s005.doc]

**Supplementary Table S5. Urine metabolite analysis against the MHFMS**

| **Analyte Name** | **Slope** | **Std** | **LCL** | **UCL** | **P-value** | **Q-value** |
| --- | --- | --- | --- | --- | --- | --- |
| VP9U_uk041 | 17.02 | 2.27 | 12.53 | 21.52 | 2.72E-11 | 4.57E-09 |
| Pantothenic acid | -10.98 | 2.18 | -15.31 | -6.65 | 2.21E-06 | 1.85E-04 |
| Uric acid | 15.66 | 3.78 | 8.17 | 23.16 | 7.20E-05 | 4.03E-03 |
| VP9U_uk088 | 6.81 | 1.67 | 3.48 | 10.13 | 9.93E-05 | 4.17E-03 |
| VP9U_UK083 | 6.82 | 1.73 | 3.38 | 10.26 | 1.55E-04 | 4.40E-03 |
| Inositol | 6.16 | 1.57 | 3.04 | 9.28 | 1.64E-04 | 4.40E-03 |
| VP9U_uk029 | 6.79 | 1.75 | 3.33 | 10.26 | 1.83E-04 | 4.40E-03 |
| IVYU_UK19 | -4.74 | 1.29 | -7.33 | -2.15 | 5.60E-04 | 1.18E-02 |
| L(-)-Malic-acid | -8.29 | 2.39 | -13.04 | -3.53 | 8.09E-04 | 1.51E-02 |
| IVYU_UK21 | 5.02 | 1.52 | 2.00 | 8.04 | 1.38E-03 | 1.78E-02 |
| VP9U_uk094 | -7.54 | 2.31 | -12.13 | -2.96 | 1.51E-03 | 1.78E-02 |
| IVYU_UK06 | 7.52 | 2.29 | 2.96 | 12.08 | 1.56E-03 | 1.78E-02 |
| unknown 31944 uk 07 | -14.28 | 4.41 | -23.03 | -5.54 | 1.63E-03 | 1.78E-02 |
| Diana_uk02 | 7.11 | 2.20 | 2.74 | 11.48 | 1.70E-03 | 1.78E-02 |
| VP9U_uk045 | -6.19 | 1.93 | -10.01 | -2.37 | 1.78E-03 | 1.78E-02 |
| VP9U_uk061 | 6.70 | 2.09 | 2.56 | 10.84 | 1.80E-03 | 1.78E-02 |
| VP9U_uk069 | 6.03 | 1.88 | 2.30 | 9.75 | 1.80E-03 | 1.78E-02 |
| 3-Methylhistidine | 3.81 | 1.22 | 1.39 | 6.22 | 2.30E-03 | 2.15E-02 |
| hexanedioic acid | -5.45 | 1.78 | -8.98 | -1.92 | 2.82E-03 | 2.41E-02 |
| VP9U_uk068 | 5.59 | 1.83 | 1.96 | 9.22 | 2.87E-03 | 2.41E-02 |
| D-Glucose-6-phosphate | -11.54 | 3.77 | -19.05 | -4.03 | 3.04E-03 | 2.44E-02 |
| IVYU_UK02 | 7.10 | 2.36 | 2.40 | 11.80 | 3.49E-03 | 2.57E-02 |
| Glycolic Acid | 10.56 | 3.54 | 3.53 | 17.58 | 3.61E-03 | 2.57E-02 |
| unknown P7881_uk 25 | 6.17 | 2.07 | 2.06 | 10.27 | 3.67E-03 | 2.57E-02 |
| unknown 31944 uk 08 | 8.54 | 2.89 | 2.82 | 14.27 | 3.85E-03 | 2.58E-02 |
| Ascorbic acid | -2.63 | 0.89 | -4.41 | -0.85 | 4.10E-03 | 2.65E-02 |
| N-(2-hydroxybenzoyl)glycine | 7.15 | 2.44 | 2.29 | 12.01 | 4.40E-03 | 2.74E-02 |
| VP9U_uk064 | 6.58 | 2.36 | 1.89 | 11.27 | 6.42E-03 | 3.85E-02 |
| Allantoin | 5.17 | 1.91 | 1.38 | 8.97 | 8.25E-03 | 4.78E-02 |
| DL-alpha-Aminoadipic acid | -6.00 | 2.31 | -10.58 | -1.42 | 1.08E-02 | 5.89E-02 |
| VP9U_uk047 | -12.45 | 4.79 | -21.96 | -2.93 | 1.09E-02 | 5.89E-02 |
| IVYU_UK05 | 5.59 | 2.23 | 1.16 | 10.02 | 1.41E-02 | 7.40E-02 |
| Hypoxanthine | 7.08 | 2.85 | 1.43 | 12.73 | 1.45E-02 | 7.41E-02 |
| Diana_uk16 | 5.59 | 2.28 | 1.07 | 10.12 | 1.59E-02 | 7.80E-02 |
| Glucuronic acid | -11.10 | 4.54 | -20.11 | -2.08 | 1.64E-02 | 7.80E-02 |
| Xanthine | 8.98 | 3.70 | 1.64 | 16.32 | 1.70E-02 | 7.80E-02 |
| P7881_uk 55 | 10.55 | 4.23 | 1.98 | 19.11 | 1.72E-02 | 7.80E-02 |
| IVYU_UK04 | -4.51 | 1.89 | -8.27 | -0.74 | 1.95E-02 | 8.63E-02 |
| VP9U_uk078 | 12.34 | 5.25 | 1.92 | 22.76 | 2.08E-02 | 8.95E-02 |
| free Sulphate | 2.78 | 1.19 | 0.42 | 5.14 | 2.14E-02 | 8.98E-02 |
| VP9U_uk067 | 14.69 | 6.32 | 2.16 | 27.23 | 2.21E-02 | 8.98E-02 |
| VP9U_uk037 | 8.85 | 3.81 | 1.28 | 16.41 | 2.24E-02 | 8.98E-02 |
| VP9U_uk030 | 4.05 | 1.76 | 0.55 | 7.55 | 2.37E-02 | 9.25E-02 |
| IVYU_UK22 | 5.23 | 2.29 | 0.68 | 9.79 | 2.48E-02 | 9.47E-02 |
| unknown 48 | 5.78 | 2.61 | 0.61 | 10.96 | 2.89E-02 | 1.08E-01 |
| VP9U_uk062 | -11.15 | 5.17 | -21.41 | -0.89 | 3.35E-02 | 1.22E-01 |
| L-Histidine | 2.70 | 1.31 | 0.11 | 5.30 | 4.15E-02 | 1.48E-01 |
| L-Tyrosine | 5.91 | 2.94 | 0.07 | 11.76 | 4.74E-02 | 1.66E-01 |
| Oxalic acid | 4.06 | 2.05 | -0.01 | 8.12 | 5.03E-02 | 1.70E-01 |
| VP9U_uk086 | 3.40 | 1.72 | -0.01 | 6.81 | 5.05E-02 | 1.70E-01 |
| Tartaric acid | -2.23 | 1.14 | -4.50 | 0.04 | 5.37E-02 | 1.77E-01 |
| cis-Aconitic acid | -9.32 | 4.87 | -18.99 | 0.35 | 5.87E-02 | 1.90E-01 |
| VP9U_uk054 | 6.07 | 3.19 | -0.27 | 12.41 | 6.02E-02 | 1.91E-01 |
| D-Glucose | 6.40 | 3.40 | -0.34 | 13.14 | 6.25E-02 | 1.94E-01 |
| VP9U_uk073 | 2.81 | 1.50 | -0.16 | 5.78 | 6.36E-02 | 1.94E-01 |
| VP9U_uk056 | 6.09 | 3.27 | -0.39 | 12.58 | 6.53E-02 | 1.96E-01 |
| VP9U_uk055 | 1.96 | 1.06 | -0.15 | 4.06 | 6.81E-02 | 2.01E-01 |
| 3-hydroxypropionic acid | -4.49 | 2.46 | -9.38 | 0.40 | 7.13E-02 | 2.06E-01 |
| VP9U_uk023 | 2.39 | 1.32 | -0.22 | 5.00 | 7.27E-02 | 2.07E-01 |
| IVYU_UK11 | 5.35 | 3.02 | -0.64 | 11.35 | 7.96E-02 | 2.20E-01 |
| Fructose | 2.35 | 1.33 | -0.28 | 4.98 | 7.97E-02 | 2.20E-01 |
| Quinic acid | -2.34 | 1.34 | -5.00 | 0.33 | 8.48E-02 | 2.30E-01 |
| IVYU_UK09 | 7.68 | 4.45 | -1.16 | 16.51 | 8.77E-02 | 2.34E-01 |
| VP9U_uk079 | -3.30 | 1.94 | -7.16 | 0.56 | 9.29E-02 | 2.44E-01 |
| IVYU_UK16 | 2.55 | 1.52 | -0.47 | 5.58 | 9.69E-02 | 2.47E-01 |
| IVYU_UK17 | -1.50 | 0.90 | -3.29 | 0.28 | 9.71E-02 | 2.47E-01 |
| o-Phosphorylethanolamine | -5.09 | 3.05 | -11.14 | 0.96 | 9.85E-02 | 2.47E-01 |
| N-methyl-4-hydroxyproline | 1.72 | 1.04 | -0.36 | 3.79 | 1.03E-01 | 2.54E-01 |
| unknown 39d | -2.41 | 1.47 | -5.32 | 0.51 | 1.04E-01 | 2.54E-01 |
| sn-Glycerol-3-phosphate | -5.69 | 3.51 | -12.65 | 1.27 | 1.08E-01 | 2.55E-01 |
| 1-Methylhistidine | 1.77 | 1.09 | -0.40 | 3.93 | 1.09E-01 | 2.55E-01 |
| VP9U_uk076 | 2.14 | 1.32 | -0.49 | 4.76 | 1.09E-01 | 2.55E-01 |
| VP9U_uk082 | 3.76 | 2.42 | -1.05 | 8.57 | 1.24E-01 | 2.86E-01 |
| VP9U_uk006 | 1.84 | 1.19 | -0.53 | 4.21 | 1.27E-01 | 2.88E-01 |
| unknown 32006\01.07.02 uk x 24 | 1.74 | 1.15 | -0.54 | 4.01 | 1.33E-01 | 2.94E-01 |
| IVYU_UK20 | -1.48 | 0.97 | -3.43 | 0.46 | 1.33E-01 | 2.94E-01 |
| Hippuric acid | 3.22 | 2.13 | -1.01 | 7.46 | 1.35E-01 | 2.94E-01 |
| IVYU_UK14 | 5.49 | 3.70 | -1.85 | 12.82 | 1.41E-01 | 3.04E-01 |
| L-Threonine | -5.06 | 3.48 | -11.96 | 1.85 | 1.49E-01 | 3.18E-01 |
| N-acetylgalactosamine | 6.78 | 4.74 | -2.63 | 16.19 | 1.56E-01 | 3.28E-01 |
| Creatinine | -4.52 | 3.22 | -10.91 | 1.86 | 1.63E-01 | 3.37E-01 |
| D-Xylose | -2.89 | 2.06 | -6.97 | 1.20 | 1.65E-01 | 3.37E-01 |
| pantoic acid | -3.11 | 2.22 | -7.54 | 1.32 | 1.66E-01 | 3.37E-01 |
| VP9U_uk066 | 5.66 | 4.10 | -2.48 | 13.80 | 1.71E-01 | 3.41E-01 |
| VP9U_uk039 | 2.32 | 1.72 | -1.09 | 5.73 | 1.80E-01 | 3.57E-01 |
| IVYU_UK13 | 1.48 | 1.11 | -0.72 | 3.69 | 1.84E-01 | 3.60E-01 |
| VP9U_uk048 | -3.06 | 2.31 | -7.64 | 1.52 | 1.87E-01 | 3.62E-01 |
| Pseudo-uridine | 7.50 | 5.78 | -3.98 | 18.97 | 1.98E-01 | 3.71E-01 |
| Pyruvic acid | 4.89 | 3.80 | -2.64 | 12.42 | 2.01E-01 | 3.71E-01 |
| Diana_uk03 | -3.09 | 2.38 | -7.89 | 1.71 | 2.01E-01 | 3.71E-01 |
| d-Mannose | 1.31 | 1.02 | -0.71 | 3.32 | 2.02E-01 | 3.71E-01 |
| unknown P7478_uk10 | -3.12 | 2.44 | -7.96 | 1.71 | 2.03E-01 | 3.71E-01 |
| unknown 32006\01.08.02 uk x 20 | 1.49 | 1.18 | -0.84 | 3.83 | 2.08E-01 | 3.76E-01 |
| Vanillic acid | -1.39 | 1.10 | -3.57 | 0.80 | 2.10E-01 | 3.76E-01 |
| Mannitol | -2.44 | 1.98 | -6.37 | 1.48 | 2.20E-01 | 3.89E-01 |
| VP9U_uk087 | 1.91 | 1.62 | -1.29 | 5.12 | 2.39E-01 | 4.18E-01 |
| VP9U_uk022 | 1.56 | 1.34 | -1.10 | 4.21 | 2.48E-01 | 4.29E-01 |
| IVYU_UK18 | -1.11 | 0.96 | -3.03 | 0.80 | 2.52E-01 | 4.31E-01 |
| 2,4-Dihydroxybutanoic acid | -4.57 | 4.06 | -12.63 | 3.49 | 2.64E-01 | 4.47E-01 |
| IVYU_UK03 | -5.52 | 4.97 | -15.38 | 4.34 | 2.69E-01 | 4.49E-01 |
| IVYU_UK08 | 2.22 | 2.00 | -1.76 | 6.20 | 2.71E-01 | 4.49E-01 |
| VP9U_uk007 | 3.50 | 3.17 | -2.79 | 9.79 | 2.72E-01 | 4.49E-01 |
| L-Phenylalanine | 5.22 | 4.78 | -4.26 | 14.71 | 2.77E-01 | 4.52E-01 |
| IVYU_UK01 | 4.91 | 4.57 | -4.17 | 13.98 | 2.86E-01 | 4.60E-01 |
| VP9U_uk084 | 6.26 | 5.85 | -5.35 | 17.87 | 2.87E-01 | 4.60E-01 |
| VP9U_uk025 | -3.36 | 3.21 | -9.72 | 3.01 | 2.98E-01 | 4.69E-01 |
| Ascorbic acid | -1.06 | 1.02 | -3.08 | 0.95 | 2.99E-01 | 4.69E-01 |
| VP9U_uk032 | 2.71 | 2.64 | -2.52 | 7.95 | 3.06E-01 | 4.76E-01 |
| Glutamic acid-internal-amide | 5.29 | 5.19 | -5.01 | 15.59 | 3.11E-01 | 4.76E-01 |
| 3-Hydroxybutanoic acid | -3.35 | 3.29 | -9.88 | 3.18 | 3.12E-01 | 4.76E-01 |
| VP9U_uk053 | -3.68 | 3.83 | -11.28 | 3.91 | 3.38E-01 | 5.08E-01 |
| L-Glutamic acid | -4.58 | 4.77 | -14.04 | 4.88 | 3.39E-01 | 5.08E-01 |
| VP9U_uk026 | -2.96 | 3.25 | -9.40 | 3.48 | 3.64E-01 | 5.40E-01 |
| L-Ornithine | -1.86 | 2.06 | -5.95 | 2.22 | 3.67E-01 | 5.40E-01 |
| L-Alanine | 3.57 | 3.96 | -4.29 | 11.43 | 3.70E-01 | 5.40E-01 |
| Arabitol | 6.66 | 7.44 | -8.10 | 21.42 | 3.73E-01 | 5.40E-01 |
| Pyrophosphate | 1.54 | 1.75 | -1.94 | 5.03 | 3.80E-01 | 5.44E-01 |
| Lactose | 1.55 | 1.76 | -1.95 | 5.05 | 3.82E-01 | 5.44E-01 |
| VP9U_uk043 | 2.41 | 2.98 | -3.50 | 8.32 | 4.21E-01 | 5.94E-01 |
| N-acetylglucosamine | -4.62 | 5.88 | -16.29 | 7.06 | 4.34E-01 | 6.03E-01 |
| L-Asparagine | 3.38 | 4.30 | -5.17 | 11.92 | 4.35E-01 | 6.03E-01 |
| N-Acetylaspartic acid | -4.39 | 5.73 | -15.76 | 6.98 | 4.46E-01 | 6.10E-01 |
| Benzoic acid | -1.10 | 1.44 | -3.95 | 1.76 | 4.47E-01 | 6.10E-01 |
| unknown 32006\01.08.02 uk x 5 | 1.09 | 1.46 | -1.82 | 3.99 | 4.60E-01 | 6.23E-01 |
| Isocitric acid | 4.81 | 7.23 | -9.54 | 19.16 | 5.08E-01 | 6.82E-01 |
| Sucrose | 0.82 | 1.27 | -1.70 | 3.34 | 5.21E-01 | 6.91E-01 |
| Arabinose | -4.06 | 6.32 | -16.59 | 8.48 | 5.22E-01 | 6.91E-01 |
| VP9U_uk009 | 1.56 | 2.46 | -3.31 | 6.44 | 5.27E-01 | 6.91E-01 |
| VP9U_uk063 | -1.12 | 1.77 | -4.64 | 2.41 | 5.30E-01 | 6.91E-01 |
| 5-Hydroxyindole-3-acetic acid | -1.80 | 3.12 | -7.99 | 4.39 | 5.65E-01 | 7.27E-01 |
| fucose | -2.29 | 3.99 | -10.21 | 5.63 | 5.67E-01 | 7.27E-01 |
| meso-Erythritol | -4.41 | 8.18 | -20.65 | 11.83 | 5.92E-01 | 7.43E-01 |
| VP9U_uk036 | -1.66 | 3.13 | -7.87 | 4.55 | 5.98E-01 | 7.43E-01 |
| VP9U_uk089 | 2.47 | 4.75 | -6.96 | 11.90 | 6.04E-01 | 7.43E-01 |
| L-Tryptophan | 1.37 | 2.66 | -3.91 | 6.64 | 6.08E-01 | 7.43E-01 |
| Diana_uk04 | 0.90 | 1.76 | -2.59 | 4.40 | 6.08E-01 | 7.43E-01 |
| IVYU_UK12 | 1.85 | 3.74 | -5.58 | 9.28 | 6.23E-01 | 7.43E-01 |
| D-Ribose | -2.02 | 4.10 | -10.16 | 6.12 | 6.23E-01 | 7.43E-01 |
| Homo-vanillic acid | -2.62 | 5.34 | -13.21 | 7.97 | 6.25E-01 | 7.43E-01 |
| IVYU_UK15 | -1.76 | 3.60 | -8.92 | 5.40 | 6.26E-01 | 7.43E-01 |
| VP9U_uk077 | -1.93 | 3.94 | -9.75 | 5.90 | 6.26E-01 | 7.43E-01 |
| Erythronic acid | -4.17 | 8.59 | -21.22 | 12.87 | 6.28E-01 | 7.43E-01 |
| IVYU_UK07 | 1.70 | 3.69 | -5.61 | 9.02 | 6.45E-01 | 7.58E-01 |
| L-4-Hydroxyproline | 1.94 | 4.28 | -6.54 | 10.43 | 6.51E-01 | 7.59E-01 |
| Benzylalcohol | -0.78 | 1.77 | -4.29 | 2.72 | 6.58E-01 | 7.62E-01 |
| VP9U_UK071 | 2.49 | 5.81 | -9.04 | 14.02 | 6.69E-01 | 7.70E-01 |
| VP9U_uk018 | 1.80 | 4.28 | -6.69 | 10.29 | 6.75E-01 | 7.71E-01 |
| Aminomalonic-acid | -0.74 | 1.81 | -4.32 | 2.84 | 6.82E-01 | 7.74E-01 |
| Citric-acid | -1.53 | 3.99 | -9.44 | 6.38 | 7.02E-01 | 7.92E-01 |
| 2-Hydroxyglutaric acid | -1.55 | 4.21 | -9.90 | 6.80 | 7.14E-01 | 7.97E-01 |
| 2-Ketoglutaric acid | 0.84 | 2.31 | -3.75 | 5.43 | 7.17E-01 | 7.97E-01 |
| VP9U_uk049 | 1.91 | 5.52 | -9.04 | 12.86 | 7.30E-01 | 8.06E-01 |
| Gluconic acid | 1.42 | 4.49 | -7.49 | 10.32 | 7.53E-01 | 8.26E-01 |
| VP9U_uk038 | -1.93 | 6.74 | -15.31 | 11.46 | 7.76E-01 | 8.46E-01 |
| unknown P7478_uk15 | 1.30 | 5.16 | -8.94 | 11.53 | 8.02E-01 | 8.69E-01 |
| Beta-Alanine | 0.50 | 2.16 | -3.80 | 4.79 | 8.18E-01 | 8.78E-01 |
| IVYU_UK10 | -0.44 | 1.92 | -4.24 | 3.37 | 8.20E-01 | 8.78E-01 |
| 4-Hydroxyphenylacetic acid | -0.73 | 3.31 | -7.29 | 5.83 | 8.27E-01 | 8.79E-01 |
| Diana_uk05 | -0.51 | 2.52 | -5.51 | 4.50 | 8.41E-01 | 8.84E-01 |
| VP9U_uk093 | 0.36 | 1.79 | -3.19 | 3.91 | 8.42E-01 | 8.84E-01 |
| DL-Lactic acid | -0.59 | 3.50 | -7.54 | 6.35 | 8.66E-01 | 9.04E-01 |
| unknown 31944 uk 15 | 0.59 | 4.66 | -8.67 | 9.84 | 9.00E-01 | 9.33E-01 |
| VP9U_uk085 | 0.23 | 1.92 | -3.58 | 4.03 | 9.07E-01 | 9.34E-01 |
| unknown P7502_UK02 | 0.25 | 2.30 | -4.32 | 4.82 | 9.14E-01 | 9.36E-01 |
| 1-Methyl uric acid | 0.09 | 1.32 | -2.54 | 2.71 | 9.48E-01 | 9.66E-01 |
| VP9U_uk090 | 0.29 | 5.11 | -9.86 | 10.43 | 9.55E-01 | 9.66E-01 |
| Myo-inositol | 0.19 | 3.85 | -7.45 | 7.84 | 9.60E-01 | 9.66E-01 |
| 2-Mono-oleoylglycerol | -0.22 | 6.24 | -12.61 | 12.16 | 9.72E-01 | 9.72E-01 |
