# Supplementary material for: Candidate Proteins, Metabolites and Transcripts in the Biomarkers for Spinal Muscular Atrophy (BforSMA) Clinical Study
Source: PLoS One. 2012 Apr 27;7(4):e35462. doi: 10.1371/journal.pone.0035462 (PMC3338723; doi:10.1371/journal.pone.0035462)
Supplement: Appendix S2 — Medication history for all subjects. (DOC) [file pone.0035462.s007.doc]

Appendix I: Table of medications used by BforSMA subjects

Legend

The goal of the BforSMA study was to reduce the confounding effects of medications and nutritional supplements by asking all subjects to be medication-free within 14 days of enrollment. The goal was achieved for putative SMA treatments currently used in the clinic, however subjects and clinicians were reluctant to alter symptomatic treatment regimens. Therefore, the study protocol allowed for the continuation of some breathing and gastrointestinal treatments and nutritional supplements. Two subjects were discovered to be on excluded medications post-enrollment: one subject was taking Robinul, a common pulmonary treatment, and another was on Ritalin. Samples collected from all subjects were utilized in the study analyses
