# Supplementary material for: Candidate Proteins, Metabolites and Transcripts in the Biomarkers for Spinal Muscular Atrophy (BforSMA) Clinical Study
Source: PLoS One. 2012 Apr 27;7(4):e35462. doi: 10.1371/journal.pone.0035462 (PMC3338723; doi:10.1371/journal.pone.0035462)
Supplement: List S1 — Members of the BforSMA Trial Group. (DOC) [file pone.0035462.s008.doc]

**Pilot Study of Biomarkers for Spinal Muscular Atrophy (BforSMA) Trial Study Group**

(Subscript denotes department and institution affiliation)

**Co-Investigators:**

Acsadi, G21

Campbell, C16

Coates, A17

Connolly, AM10

Crawford, TO1

Darras, BT5

Finkel, RS2A

Iannaccone, ST7

Kaufmann, P3

Khongkhatithum, C7

Kissel, JT11

Kolb, SJ11

Kunz, NL15

Makris, C13

Mathews, KD8A

Matthews, DJ9

Schroth, MK18

Swoboda, KJ4

Vajsar, J17

Wang, CH12

Wong,BL14

**BforSMA Evaluators**

Antiel, L15

Bell, M20

Gee, R12

Glanzman, AM2B

Hartman, J1

King, W11

Krosschell, K19

Laubenthal, K8B

McGuire, M14

Montes, J3

Nelson, L7

Owen, J17

Patterson, K18

Quigley, J5

Roman, C6

Riley, S5

Scholtes, C16

Sedlacek, P13

Siener, C10

Strumpf, S9

Wood, J4

**BforSMA Study Coordinators**

Andersen, M7

Benton, M2

Binner, H15

Butler, H5

Chelnik, S11

Clayton, J9

Jamal, A6

MacMillan, L17

Mertensen, M4

Montgomery, M3

Morehart, P14

Pharo, L18

Reeves, G13

Stephan, C8B

Taranik, R16

Tsai, O1

Wedell, V12

Wulf, C10

1 Departments of Neurology and Pediatrics, Johns Hopkins University

2A Departments of Neurology and Pediatrics, The Children's Hospital of Philadelphia and University of Pennsylvania School of Medicine

2B Department of Physical Therapy, The Children's Hospital of Philadelphia and University of Pennsylvania School of Medicine

3 Department of Neurology, Columbia University Medical Center

4 Departments of Neurology and Pediatrics, University of Utah School of Medicine

5 Department of Neurology, Children's Hospital Boston and Harvard Medical School

6 Departments of Pediatrics and Neurology, Wayne State University and Children’s Hospital of Michigan

7 Department of Pediatrics, University of Texas Southwestern Medical Center

8A Departments of Pediatrics and Neurology, University of Iowa Carver College of Medicine

8B Department of Pediatrics, University of Iowa Carver College of Medicine

9 Department of Physical Medicine and Rehabilitation, University of Colorado

10 Department of Neurology, Washington University

11 Departments of Molecular & Cellular Biochemistry and Neurology, The Ohio State University

12 Department of Neurology and Neurological Sciences, Stanford University

13 Department of Pediatrics, University of Alabama at Birmingham

14 Division of Pediatric Neurology, Cincinnati Children's Hospital Medical Center

15 Department of Neurology, Mayo Clinic

16 Department of Paediatrics, Clinical Neurological Sciences and Epidemiology, University of Western Ontario, London Health Sciences Centre

17 Division of Neurology, The Hospital for Sick Children and University of Toronto

18 Department of Pediatrics, University of Wisconsin

19 Department of Physical Therapy and Human Movement Sciences, Northwestern University

20 New England Research Institutes

21 Connecticut Children's Medical Center, Hartford, CT

**Institutional Review Boards for the BforSMA Study Cohort**

Children's Hospital of Boston Institutional Review Board, Office of Clinical Investigation

The Children's Hospital of Philadelphia Institutional Review Board

Cincinnati Children's Hospital Medical Center Institutional Review Board

Colorado Multiple Institutional Review Board - University of Colorado Denver IRB

Columbia University Medical Center Institutional Review Board

Johns Hopkins Medical Institutional Review Board

Mayo Clinic Institutional Review Board

New England Research Institutes Institutional Review Board

The Ohio State University Biomedical Institutional Review Board

Research Ethics Board for The Hospital for Sick Children

Stanford University Institutional Review Board

University of Alabama at Birmingham Institutional Review Board for Human Use

University of Iowa Institutional Review Board

The University of Utah Institutional Review Board

The University of Western Ontario Research Ethics Board for Health Sciences Research

University of Wisconsin Health Sciences Institutional Review Board

The UT Southwestern Institutional Review Board

Washington University in St. Louis Institutional Review Board

Wayne State University Institutional Review Board, Human Investigation Committee
